# *Gemella morbillorum* Promotes Colorectal Carcinogenesis: LPBDCP‐Mediated Invasion Activates Ras Signaling and Destabilizes p53

**DOI:** 10.1002/advs.202517245

**Published:** 2026-04-07

**Authors:** Zhen Wang, Jia Zhang, Haojie Lu, Jingjing Ni, Shuaishuai Yang, Yuting Shi, Shuo Zhang, Pei Zhang, Li Liu

**Affiliations:** ^1^ Department of Epidemiology and Biostatistics School of Public Health Ministry of Education Key Lab of Environment and Health Tongji Medical College Huazhong University of Science and Technology Wuhan Hubei P. R. China; ^2^ Public Health Service Center, Bao'an District, Shenzhen Shenzhen Guangdong P. R. China; ^3^ Institutional Center For Shared Technologies and Facilities of Wuhan Institute of Virology Chinese Academy of Sciences Wuhan Hubei P. R. China; ^4^ Hubei Cancer Hospital Tongji Medical College Huazhong University of Science and Technology Wuhan Hubei P. R. China; ^5^ Hubei Provincial Clinical Research Center for Colorectal Cancer Wuhan Hubei P. R. China; ^6^ Wuhan Clinical Research Center For Colorectal Cancer Wuhan Hubei P. R. China

**Keywords:** Ca^2^^+^, colorectal cancer, *Gemella morbillorum*, p53, RASA4

## Abstract

Gut microbiota dysbiosis promotes colorectal cancer (CRC) tumorigenesis. A global fecal metagenomic analysis identified *Gemella morbillorum* as a key contributor to the CRC‐associated microbiota. Fluorescence in situ hybridization revealed that *Gemella morbillorum* is enriched in CRC tumor tissues compared to adjacent normal tissues. In vitro and in vivo experiments elucidated the oncogenic effects of *Gemella morbillorum* on human CRC cell lines and mouse models. Multimodal imaging shows that *Gemella morbillorum* can internalize into host cells. RNA sequencing, co‐immunoprecipitation, and mass spectrometry identified that *Gemella morbillorum* invades host cells via interaction between its LysM peptidoglycan‐binding domain protein (LPBDCP) and host cell surface transmembrane protein TMEM140. This invasion triggers Ca^2^^+^ influx, downregulates RASA4, and activates the PI3K‐AKT‐NF‐κB and RAF‐MEK‐ERK signaling pathways. Following invasion, *Gemella morbillorum* secretes NAD‐dependent protein deacetylase (NDPD), which induces p53 deacetylation and degradation. Collectively, these events accelerate cell proliferation, shorten the cell cycle, and inhibit apoptosis, thereby promoting malignant transformation. Genetic knockout of LPBDCP or TMEM140 effectively inhibits bacterial invasion and abrogates the oncogenic effects of *Gemella morbillorum*. In tumor‐bearing mice, knockout of LPBDCP or NDPD eliminates the tumor‐promoting effects of *Gemella morbillorum*. These results underscore *Gemella morbillorum*’s role in CRC and pinpoint potential intervention targets.

## Graphical abstract

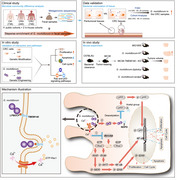

## 1 Introduction

The human microbiota, with a cellular abundance comparable to that of human cells, forms a complex ecosystem. In the gastrointestinal tract, microbial communities have evolved interdependent biochemical networks that interact with host cells [1]. Maintaining gut microbiome homeostasis is crucial for the host's health, as gut bacteria, fungi, and viruses are linked to gastrointestinal diseases [2].

Colorectal cancer (CRC) is the fourth deadliest cancer globally, causing nearly 900 000 deaths annually [3]. Research has shown that the gut microbiome plays a significant role in CRC development. Alterations in gut microbial diversity, components and structures, known as “gut dysbiosis,” are associated with colorectal carcinogenesis [4]. For example, the depletion of beneficial bacteria like *Roseburia intestinalis* and *Bifidobacterium adolescentis*, which produce tumor‐opposing metabolites such as butyrate, is linked to faster colorectal carcinogenesis [5, 6]. Conversely, pathobionts like *Fusobacterium nucleatum*, *Bacteroides fragilis*, and enterotoxigenic *Escherichia coli*, could promote tumor growth and metastasis by affecting the tumor microenvironment or inducing changes in the phenotype of somatic cells [7, 8]. An expanding fecal microbiota analysis in CRC patients has uncovered additional taxa that may influence CRC risk [9]. To achieve precision intervention for CRC, it is urgent to identify the gut bacteria associated with CRC and their mechanisms in driving cancer initiation and progression.

Our research used shotgun metagenomics to investigate the association between gut microbiota and CRC. By integrating multi‐cohort data, we identified the oral–gut commensal *Gemella morbillorum*—a facultative anaerobic, Gram‐positive coccus historically implicated in invasive endocarditis—as a potential driver of colorectal cancer [10, 11]. A recent report has also shown a link between *Gemella morbillorum* and CRC [12]. We further explored the mechanisms underlying this process and found that *Gemella morbillorum* utilizes LysM peptidoglycan‐binding domain‐containing protein (LPBDCP)—Transmembrane Protein 140 (TMEM140) binding to facilitate cellular invasion, triggering intracellular Ca^2^^+^ accumulation, and depleting RAS p21 protein activator 4 (RASA4) pools, which leads to sustained GTP‐Ras accumulation and activation of PI3K‐AKT‐NF‐κB and RAF‐MEK‐ERK pathways. Additionally, *Gemella morbillorum* secretes NAD‐dependent protein deacetylase (NDPD) effector proteins that destabilize p53 through deacetylation‐mediated proteasomal degradation. These mechanisms drive CRC cell cycle progression and confer resistance to apoptosis.

## 2 Results

### 2.1 *Gemella morbillorum* Enrichment in Fecal Samples and Tissues of CRC Patients

**FIGURE 1:**
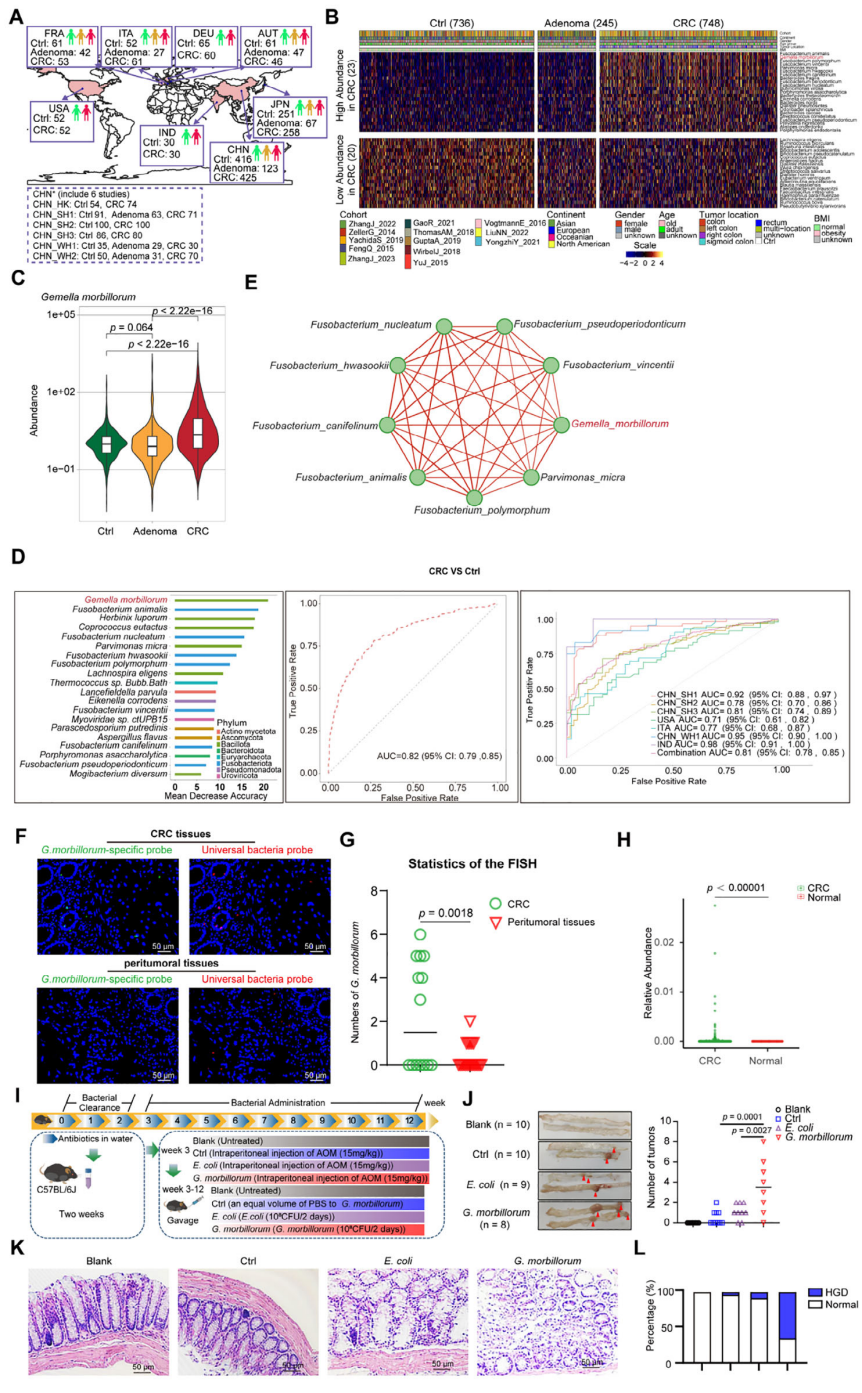
*G. morbillorum* is enriched in fecal samples and tissues of CRC patients and promotes colorectal tumorigenesis in mice. (A) Fecal metagenomic datasets from 13 cohorts (2 in‐house, 11 public) with geographical distribution and cohort sizes. (B) Heatmap of fecal microbiome alterations in CRC (*n* = 748), colorectal adenoma (CRA, *n* = 245), and control groups (Ctrl, *n* = 736). (C) Abundance of *G. morbillorum* in pooled cohorts across CRC, CRA, and control groups. (D) Multi‐Kingdom microbial model for CRC diagnosis. Left: Impact of microbial classifiers on model accuracy. Middle: ROC curve in discovery dataset. Right: ROC curves in validation datasets. (E) Interaction network of *G. morbillorum* with other species in CRC fecal samples (red lines indicate positive correlations). (F) Representative FISH showing bacterial probes (red) and *G. morbillorum*‐specific probes (green) within colorectal tumor and adjacent peritumoral tissues. Scale bar = 50 µm. (G) A comparison of *G. morbillorum* abundance between CRC tissues (*n* = 14) and peritumoral tissues (*n* = 20). (H) Relative abundance of *G. morbillorum* in 622 CRC tissues and 51 controls from TCGA (not detected in controls). (I) Schematic of *G. morbillorum* intervention in an AOM‐induced murine CRC model. AOM: Azoxymethane (J) The left panel shows the number and size of colorectal tumors in murine CRC models across various treatment groups (Blank, *n* = 10; Ctrl, *n* = 10; *E. coli* MG1655, *n* = 9; *G. morbillorum*, *n* = 8). The right panel provides a statistical analysis of differences in colorectal tumor quantification among these groups. (K) H&E‐stained colorectal tissue sections from each treatment group. Scale bar = 50 µm. (L) Proportion of high‐grade dysplasia (HGD) in colon tissue samples from various treatment groups. Data are presented as mean ± SD. Comparisons between two groups were performed using Student's *t*‐test. Multi‐group comparisons were conducted using one‐way ANOVA followed by Tukey's post‐hoc test. The significance level (α) was set at 0.05 (two‐tailed).

We collected fecal metagenomic data from 13 cohorts, including two in‐house cohorts (CHN_WH1 and CHN_WH2) and 11 publicly available cohorts (Figure 1A), which yielded 1729 high‐quality samples after quality filtering (748 CRC, 245 Colorectal adenoma (CRA), 736 healthy controls, details in Figure S1A). Specifically, bacteria such as *Fusobacterium animalis* and *G. morbillorum* were highly enriched in CRC patients compared to healthy controls (Figure 1B), with consistent elevation in CRC compared to CRA (Figure 1C and Figure S1B).

The random forest algorithm identified a multi‐kingdom diagnostic model to distinguish CRC from healthy controls, using 20 markers (16 bacteria, 2 fungi, 1 virus, and 1 archaeon). The model achieved discovery/validation AUCs of 0.82 (95% CI: 0.79‐0.85) and 0.81 (95% CI: 0.78‐0.85), respectively (Figure 1D). Among the microbial classifiers, *G. morbillorum* contributed the most to the model. Co‐occurrence networks revealed strong positive correlations between *G. morbillorum* and *Fusobacterium* species within the CRC microbiota (Figure 1E and Figure S1C).

Fluorescence in situ hybridization (FISH) analysis of 14 CRC tissues and 20 peritumoral tissues revealed significant enrichment of *G. morbillorum* in CRC tissues (Figure 1F,G). Consistently, a Pan‐cancer atlas of the tumor‐resident microbiome study found elevated *G. morbillorum* levels in 622 CRC tissues vs. 51 adjacent controls (Figure 1H) [13].

### 2.2 *Gemella morbillorum* Promotes Colorectal Tumorigenesis in Murine Model

AOM‐induced CRC mice received a 2‐week antibiotic cocktail treatment (Figure 1I). The qPCR fecal monitoring tracked the dynamics of total bacteria and *G. morbillorum* abundance. Within 2 weeks, total bacterial counts in the mouse gut decreased significantly, and *G. morbillorum* fell below the detection limit (Figure S2A). Mice then received oral gavages of 10^8^ CFU of nonpathogenic *E. coli* MG1655 or *G. morbillorum* every other day. Daily fecal analysis demonstrated the colonization dynamics of *G. morbillorum*, which peaked at week 4 and persisted throughout the experimental period (Figure S2B). At the terminal endpoint analysis at week 12, *G. morbillorum* colonized mice exhibited increased tumor burden compared to controls (Figure 1J). H&E staining of representative tumor sections revealed marked histopathological progression in *G. morbillorum* colonized mice, with significant nuclear atypia and glandular distortion compared to blank, control, and *E. coli* MG1655 treated groups (Figure 1K,L). Additionally, longitudinal monitoring showed significant body mass loss in the *G. morbillorum*‐colonized group (Figure S2C).

### 2.3 *Gemella morbillorum* Promotes Proliferation, Inhibits Apoptosis, and Accelerates Cell Cycle of Colon Cancer Cells

**FIGURE 2:**
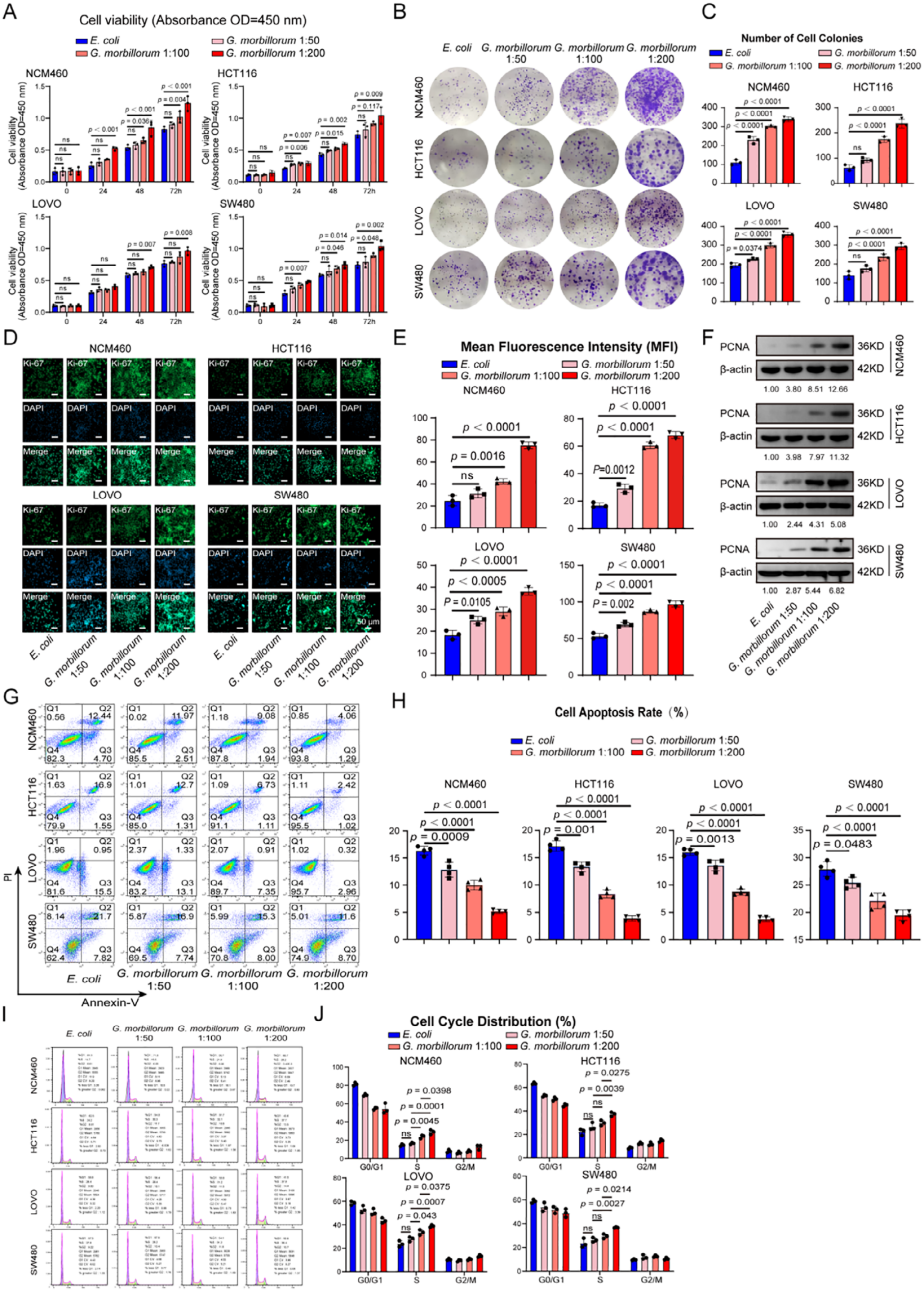
*G. morbillorum* promotes proliferation, inhibits apoptosis, and accelerates cell cycle progression. (A) Effects of *G. morbillorum* and MG1655 on cell viability of intestinal epithelial and CRC cells assessed by CCK‐8 assay. (B) Representative images of effects of *G. morbillorum* on colony formation in normal intestinal epithelial and CRC cells. Images are representative of *n* = 3 biologically independent experiments. (C) Statistical analysis of colony numbers. (D) Effects of *G. morbillorum* on Ki‐67 expression in normal intestinal epithelial and CRC cells, shown by representative images with nuclei stained in blue (DAPI) and Ki‐67 in green. Images are representative of *n* = 3 biologically independent experiments. (E) Statistical analysis of Ki‐67 mean fluorescence intensity. (F) Effects of *G. morbillorum* on PCNA expression in normal intestinal epithelial and CRC cells. (G) Apoptosis was analyzed by flow cytometry, with apoptosis rate defined as the sum of late apoptosis (Q2) and early apoptosis (Q3) percentages. Representative plots from *n* = 4 biologically independent experiments. (H) Statistical analysis of apoptosis rates in different groups. (I) Representative histograms showing cell cycle distribution (G0/G1, S, and G2/M phases) for each cell line under different treatment conditions. Representative histograms from *n* = 3 biologically independent experiments. (J) Statistical analysis of cell cycles, with bar graphs summarizing the percentage of cells in each phase (G0/G1, S, and G2/M). Data are presented as mean ± SD. Multi‐group comparisons were performed using one‐way ANOVA followed by Tukey's post‐hoc test. The significance level (α) was set at 0.05 (two‐tailed).

Normal intestinal epithelial cells (NCM460) and colon cancer cells (HCT116, LOVO, SW480) were incubated anaerobically for 0 (baseline), 1.0, 1.5, and 2 h. Cell viability assays showed no significant differences within 1.5 h (Figure S3A,B), leading to the selection of a 1.5 h co‐incubation duration. The four cell lines were then co‐incubated daily for 1.5 h over 3 days with *G. morbillorum* or *E. coli* MG1655 bacterial suspensions (OD600 = 0.5) at MOIs of 50, 100, and 200. Cell viability was monitored using the CCK‐8 assay. Results showed that *G. morbillorum* co‐culture increased cell viability in a concentration‐dependent manner, with the highest effect at MOI 200 on day 3 (Figure 2A). Colony formation assays revealed proportional increases in colony numbers with *G. morbillorum* concentrations (Figure 2B,C). This pro‐proliferation effect was confirmed by Ki‐67 staining and PCNA expression, both showing dose‐dependent proliferation (Figure 2D–F). Additionally, apoptosis decreased in a dose‐dependent manner with increasing *G. morbillorum* exposure (Figure 2G,H). Cell cycle analysis showed increased S‐phase cell proportions in a concentration‐dependent manner (Figure 2I,J), indicating the role of *G. morbillorum* in promoting G1/S phase transition during DNA replication.

### 2.4 *Gemella morbillorum’*s Tumor‐Promoting Effect Mediated by Direct Bacterial Contact, Not Secreted Factors

**FIGURE 3:**
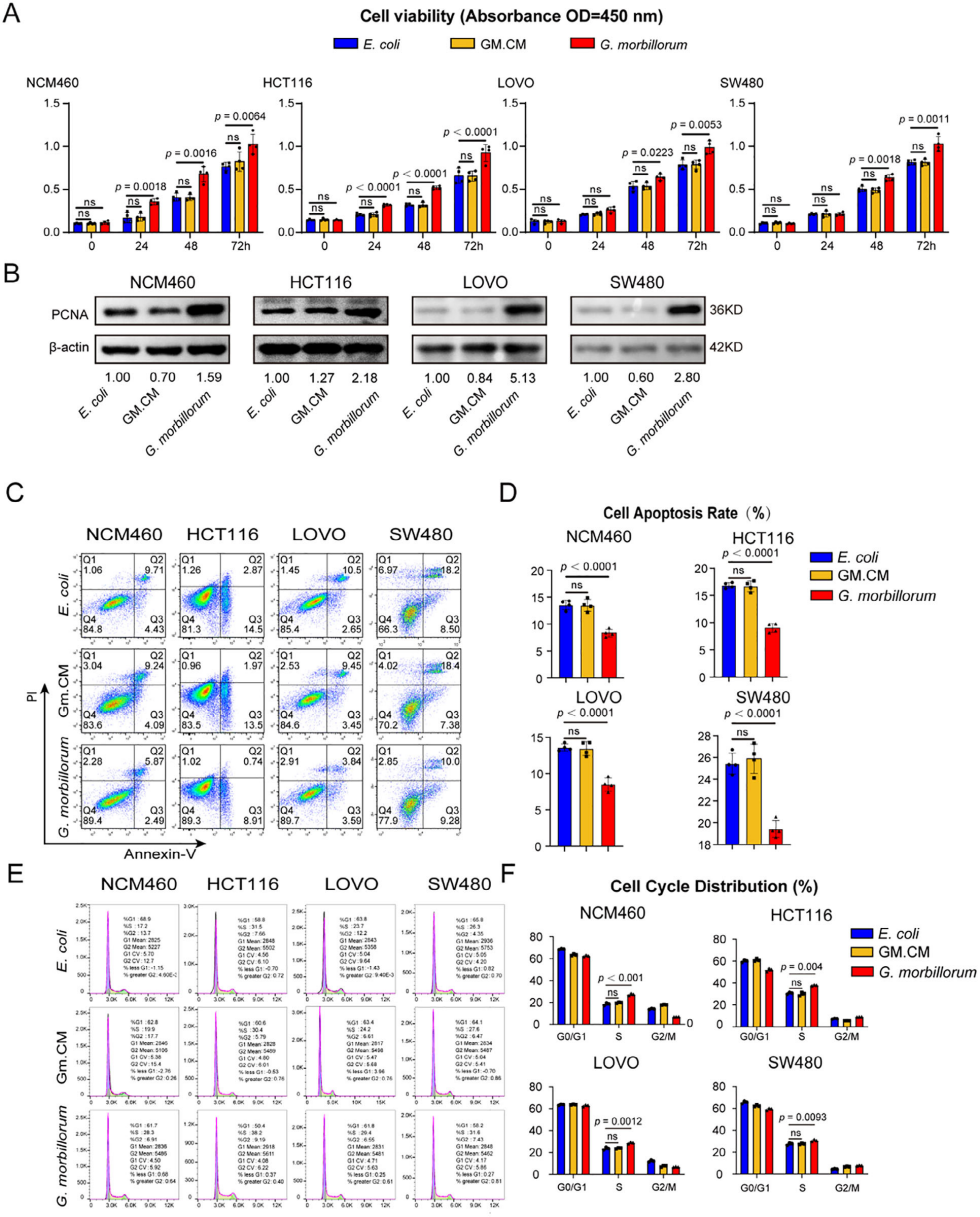
Direct contact with *G. morbillorum* modulates proliferation, apoptosis, and cell cycle. (A) The CCK‐8 assay assessed the effects of viable *G. morbillorum*, its conditioned medium, and *E. coli* MG1655 on cell viability at MOI 200 and a 1:50 medium ratio. (B) Western blot analysis of PCNA expression levels regulated by viable *G. morbillorum*, its conditioned medium, and *E. coli* MG1655. (C) Flow cytometry assessed apoptosis induced by viable *G. morbillorum*, its conditioned medium, and *E. coli* MG1655. Representative images are shown. Representative plots from *n* = 4 biologically independent experiments. (D) Statistical analysis of apoptosis rates among treatment groups, based on three replicates. (E) Cells treated with viable *G. morbillorum*, its conditioned medium, and MG1655 were PI stained and analyzed by flow cytometry for cell cycle differences. Representative images are shown. Representative histograms from *n* = 3 biologically independent experiments. (F) Statistical analysis of S‐phase cell proportions across treatment groups, based on three replicates. Data are presented as mean ± SD. Multi‐group comparisons were performed using one‐way ANOVA followed by Tukey's post‐hoc test. The significance level (α) was set at 0.05 (two‐tailed).

To investigate whether *G. morbillorum* promotes cell proliferation through direct contact or secreted factors, we separated *G. morbillorum* cultures into viable bacteria and 0.22 µm filtered conditioned medium. These fractions, along with an *E. coli* MG1655 control, were co‐cultured with four cell lines under anaerobic conditions (MOI = 200, 1.5 h/day for 3 days). CCK‐8 assays showed that viable *G. morbillorum* significantly enhanced cell proliferation compared to the *E. coli* MG1655 control, while the conditioned medium had no significant effect (Figure 3A).

Western blot analysis revealed higher PCNA protein levels in cells treated with viable *G. morbillorum* than in those treated with conditioned medium or *E. coli* MG1655 controls (Figure 3B). Flow cytometry assays further showed that viable *G. morbillorum*, but not its conditioned medium, significantly reduced apoptosis and increased S‐phase cell populations compared to *E. coli* MG1655 controls (Figure 3C–F).

### 2.5 The Interaction Between *Gemella morbillorum* and the Host is a Process of Adhesion and Internalization

**FIGURE 4:**
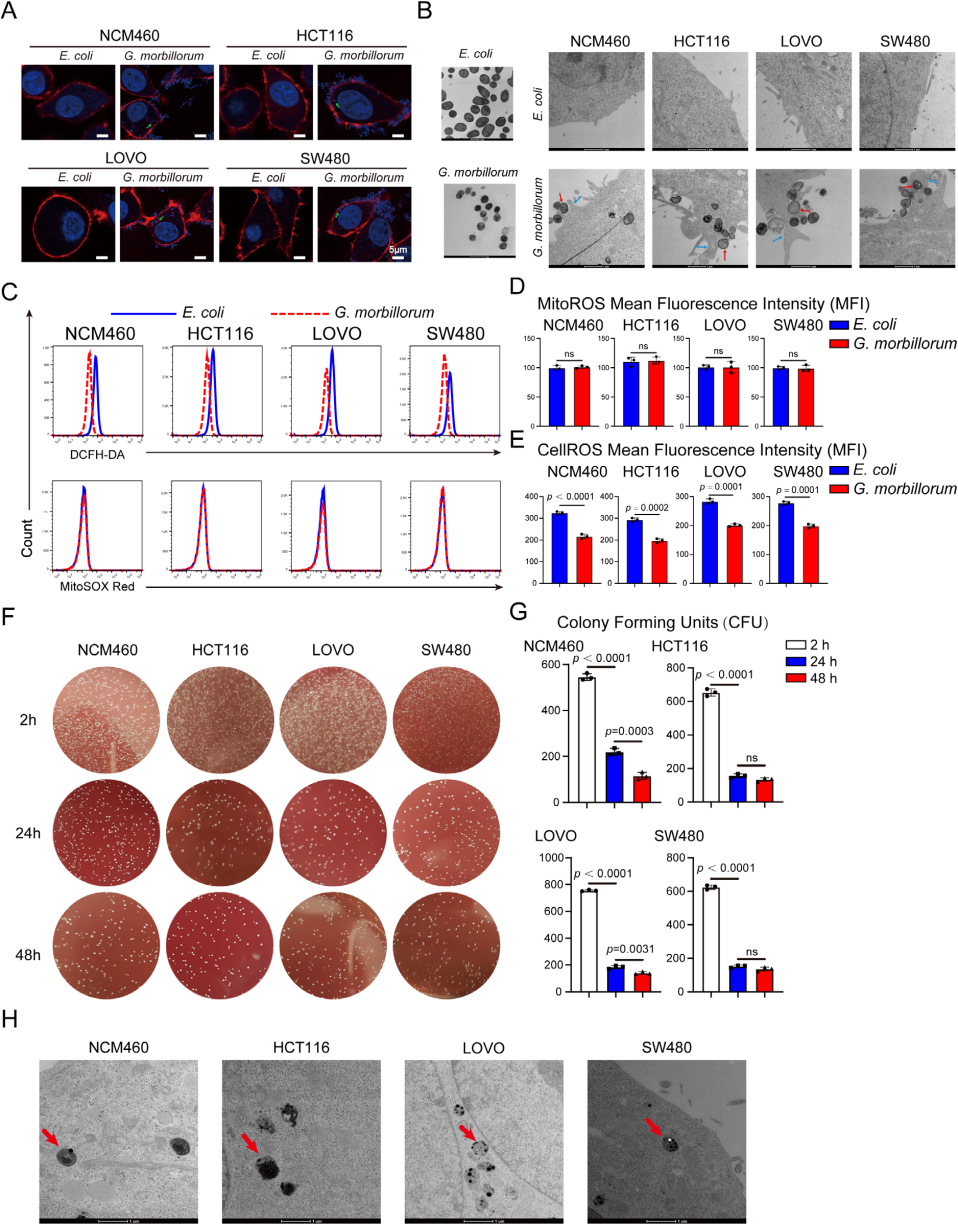
The internalization of *G. morbillorum* into cells is an active invasion process. (A) Confocal microscopy captured the interaction of *G. morbillorum* or *E. coli* MG1655 with host cells after 0.5 h of co‐culture. Representative images of the *G. morbillorum* group highlight DAPI‐stained bacteria (blue) inside the cells, with the cell membrane in red and the nucleus in blue. Green arrows point to the internalized bacteria. (B) The membrane‐proximal interaction of *G. morbillorum* or *E. coli* MG1655 with host cells assessed by electron microscopy. Blue arrows point to cell pseudopodia, while red arrows highlight bacteria that are being internalized. (C) Representative images of flow cytometry detecting MitoROS (MitoSOX Red) in cells and CellROS (DCFH‐DA). Representative plots from *n* = 3 biologically independent experiments. MitoROS, Mitochondria‐derived reactive oxygen species; CellROS, Total intracellular reactive oxygen species. (D) Statistical analysis of mitochondria‐derived ROS levels in cells treated with *G. morbillorum* or *E. coli* MG1655. (E) Statistical analysis of total cellular ROS levels in cells treated with *G. morbillorum* or *E. coli* MG1655. (F) Cells treated with *G. morbillorum* were lysed at various time points after gentamicin (200 µg/mL) treatment, and the resulting lysates were cultured on solid agar plates for 36 h to observe colony formation. Images are representative of *n* = 3 biologically independent experiments. (G) Statistical analysis of differences in colony formation numbers across groups at various time points. (H) Transmission electron microscopy observation of intracellular survival of *G. morbillorum* in cells co‐cultured for 24 h, with arrows indicating intracellular *G. morbillorum*. Data are presented as mean ± SD. Comparisons between two groups were performed using Student's *t*‐test. Multi‐group comparisons were conducted using one‐way ANOVA followed by Tukey's post‐hoc test. The significance level (α) was set at 0.05 (two‐tailed).

As shown in the previous results, *G. morbillorum* affects cellular phenotypes through its bacterial body. To verify the interaction between *G. morbillorum* and the cells, we established a polarized co‐culture model. Confocal laser scanning microscopy (CLSM) at 30 min post‐inoculation revealed robust bacterial adhesion to all four cell lines (NCM460, HCT116, LOVO, and SW480), accompanied by efficient internalization events (Figure 4A). In contrast, *E. coli* MG1655 showed no adhesion or internalization under the same conditions. Ultrastructural analysis by transmission electron microscopy (TEM) revealed orchestrated internalization of *G. morbillorum* through dual membranous remodeling events: host‐derived plasma membrane invagination coordinated with bacterial‐induced pseudopod‐like extensions, ultimately localizing within cytoplasmic compartments (Figure 4B). *E. coli* MG1655, however, displayed no colonization or intracellular localization in control groups. These results indicate that the interaction between *G. morbillorum* and the host is a process of adhesion and internalization.

### 2.6 The Internalization of *Gemella morbillorum* Into Cells is an Active Invasion Process

Upon internalization, *G. morbillorum* may face different outcomes. In one scenario, like *E. coli* engulfed by macrophages, the bacteria are transported to lysosomes for degradation via phagocyte oxidase (Phox)‐dependent respiratory burst and mitochondrial reactive oxygen species (ROS) production [14, 15]. Alternatively, *G. morbillorum* may evade like *Listeria monocytogenes*, escaping phagosomes via hemolysin O and phospholipase C, then spreading intercellularly via ActA‐driven actin motility while depleting PI3P to evade autophagy [16]. To determine if *G. morbillorum* internalization is passive or active, we analyzed mitochondrial ROS (MitoROS) and total cytosolic ROS (CellROS) in four cell lines after 3 days exposure to *G. morbillorum*/*E. coli* MG1655 (1.5 h/day). The level of mitochondrial‐derived MitoROS remained unchanged, indicating the absence of a phagocytic ROS response, whereas the CellROS level decreased, an unexpected finding (Figure 4C–E). We lysed cells treated with *G. morbillorum* at 2, 24, and 48 h, cultured lysates anaerobically, and counted colonies. Colony counts declined from 24 to 48 h without significant differences, indicating prolonged bacterial survival (Figure 4F,G). TEM of 24‐h co‐cultured cells confirmed the presence of *G. morbillorum* (Figure 4H). Collectively, these results indicate that *G. morbillorum* undergoes active invasion with sustained intracellular survival.

### 2.7 *Gemella morbillorum* Invasion of Cells is Dependent on the Cell Membrane Protein TMEM140

**FIGURE 5:**
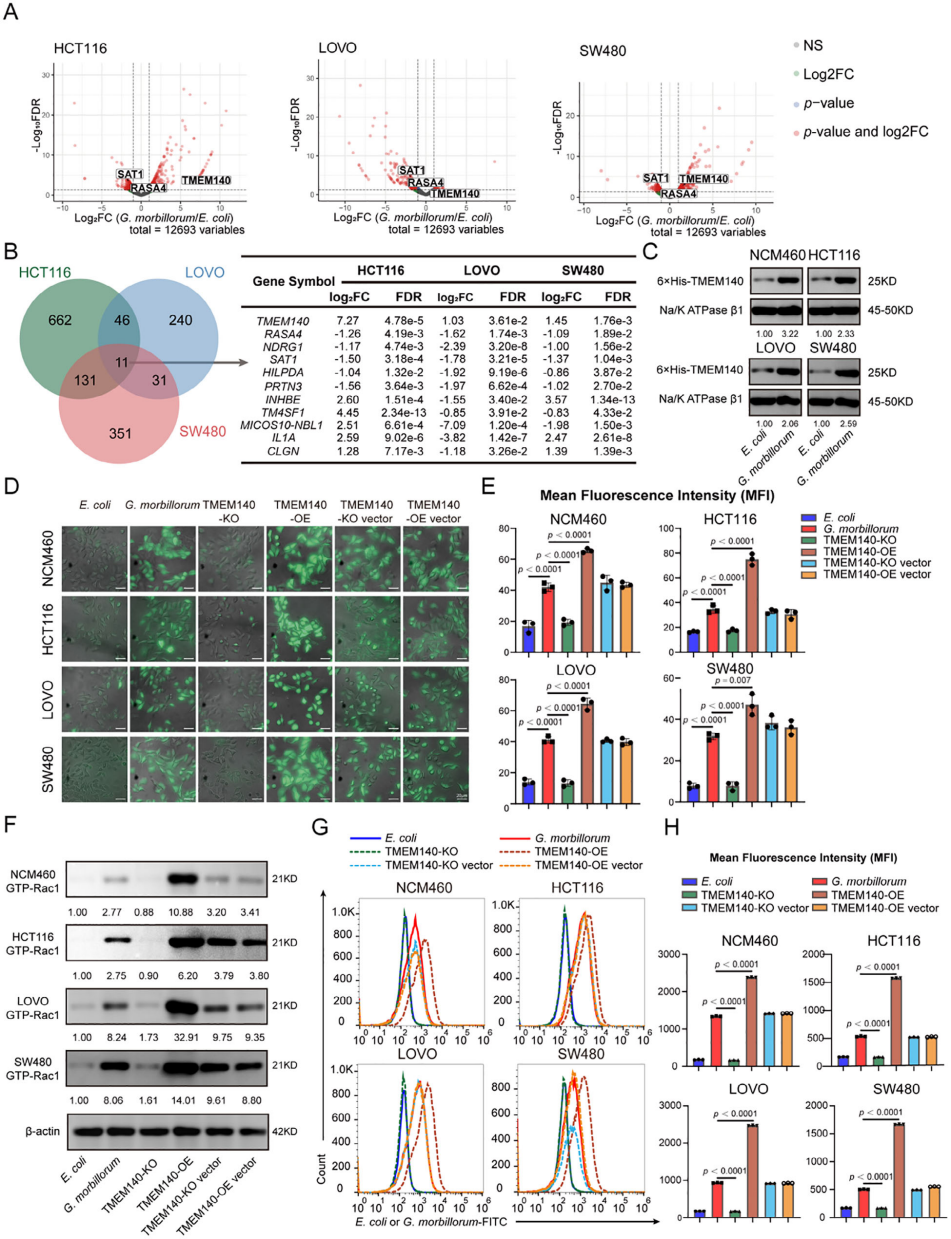
*G. morbillorum* invasion of cells is dependent on the cell membrane protein TMEM140. (A) Volcano plots of differentially expressed genes from RNA sequencing of HCT116, LOVO, and SW480 cells treated with *G. morbillorum* or *E. coli* MG1655. (B) The left panel shows the Venn diagram of differentially expressed genes across various cell lines, while the right panel displays the basic information of commonly altered genes. (C) Western blot was used to detect the effects of *G. morbillorum* or *E. coli* MG1655 treatment on the membrane localization of TMEM140. (D) Representative images showing the effects of TMEM140‐KO and TMEM140‐OE on Ca^2^^+^ (green) changes mediated by *G. morbillorum* invasion, detected by the Fluo‐4 probe. Images are representative of *n* = 3 biologically independent experiments. TMEM140‐KO: Cell‐TMEM140‐knockout; TMEM140‐OE: Cell‐TMEM140‐overexpression. (E) Differences in intracellular Ca^2^^+^ mean fluorescence intensity (MFI) were statistically analyzed. (F) Western blot was conducted to detect the effects of TMEM140‐KO and TMEM140‐OE on the intracellular GTP‐Rac1 levels during *G. morbilloru*m invasion of cells. (G) Representative images of flow cytometry analysis showing the effects of TMEM140‐KO and TMEM140‐OE on the number of cells invaded by *G. morbillorum*. *G. morbillorum* and *E. coli* MG1655 were pre‐stained with CFDA‐SE. Representative plots from *n* = 3 biologically independent experiments. (H) Statistical analysis of the differences in the invasion capacity of *G. morbillorum* due to TMEM140‐KO and TMEM140‐OE. Data are presented as mean ± SD. Multi‐group comparisons were performed using one‐way ANOVA followed by Tukey's post‐hoc test. The significance level (α) was set at 0.05 (two‐tailed).

To characterize host cell surface receptors mediating *G. morbillorum* cellular invasion, RNA sequencing was conducted on three CRC cell lines treated with *G. morbillorum* or *E. coli* MG1655. We found that *G. morbillorum* induced 662, 240, and 351 differentially expressed genes in HCT116, LOVO, and SW480, respectively, compared to *E. coli*. Eleven genes were commonly altered, with six (*TMEM140*, *RASA4*, *NDRG1*, *SAT1*, *PRTN3*, *HILPDA*) showing consistent expression trends across the three cell lines (Figure 5A,B). Among the six consistently expressed genes in three cell lines, only one transmembrane protein, TMEM140 (Transmembrane Protein 140), was upregulated in all cell lines. Previous studies have shown that TMEM140 silencing inhibits glioma cell viability, migration, and invasion [17]. Bulk tissue expression profiling revealed TMEM140 expression across the gastrointestinal tract, with relatively high levels in the colon (Figure S4A). Given the oral‐gastrointestinal tropism of *G. morbillorum* and its direct access to colonic epithelia, the enriched expression of TMEM140 in colonic tissues establishes a critical host microenvironment conducive to bacterial modulation of colorectal inflammatory or neoplastic diseases. This hypothesis is supported by the observation that *G. morbillorum* abundance is significantly elevated in inflammatory bowel disease (IBD) patients compared to healthy controls (Figure S4B). Western blot analysis showed that *G. morbillorum* treatment significantly increased cell membrane TMEM140 levels compared to *E. coli* MG1655 (Figure 5C), indicating a potential role for TMEM140 in mediating *G. morbillorum*—CRC cell contact. *Listeria monocytogenes* uses its pore‐forming toxin LLO to drive Ca^2^^+^ influx during cell invasion. Similarly, macrophage phagocytosis of *Escherichia coli* also induces calcium ion influx. This is likely because Ca^2^^+^ can activate Ca^2^^+^‐dependent protein kinase II (CaMKII) and Hippo kinases Mst1/2, leading to Rac1‐dependent actin rearrangement, which changes cell shape and boosts motility [18, 19, 20]. Subsequently, we treated cells co‐incubated with *G. morbillorum* or *E. coli* MG1655 using Fluo‐4 (Ca^2^^+^ probe) and detected intracellular Ca^2^^+^ levels via fluorescence microscopy. *G. morbillorum* significantly increased Ca^2^^+^ levels compared to *E. coli* MG1655 in 5 min. This effect was enhanced by TMEM140 overexpression but abolished by TMEM140 knockout (Figure 5D,E). Western blot analysis showed that TMEM140 overexpression elevated GTP‐Rac1 (active Rac1) levels, an effect negated by TMEM140 knockout (Figure 5F). Flow cytometry also demonstrated that TMEM140 overexpression enhanced *G. morbillorum* invasive capacity, whereas TMEM140 knockout reduced it (Figure 5G,H).

### 2.8 *Gemella morbillorum* Host Cell Invasion is Mediated by Bacterial Protein LPBDCP

**FIGURE 6:**
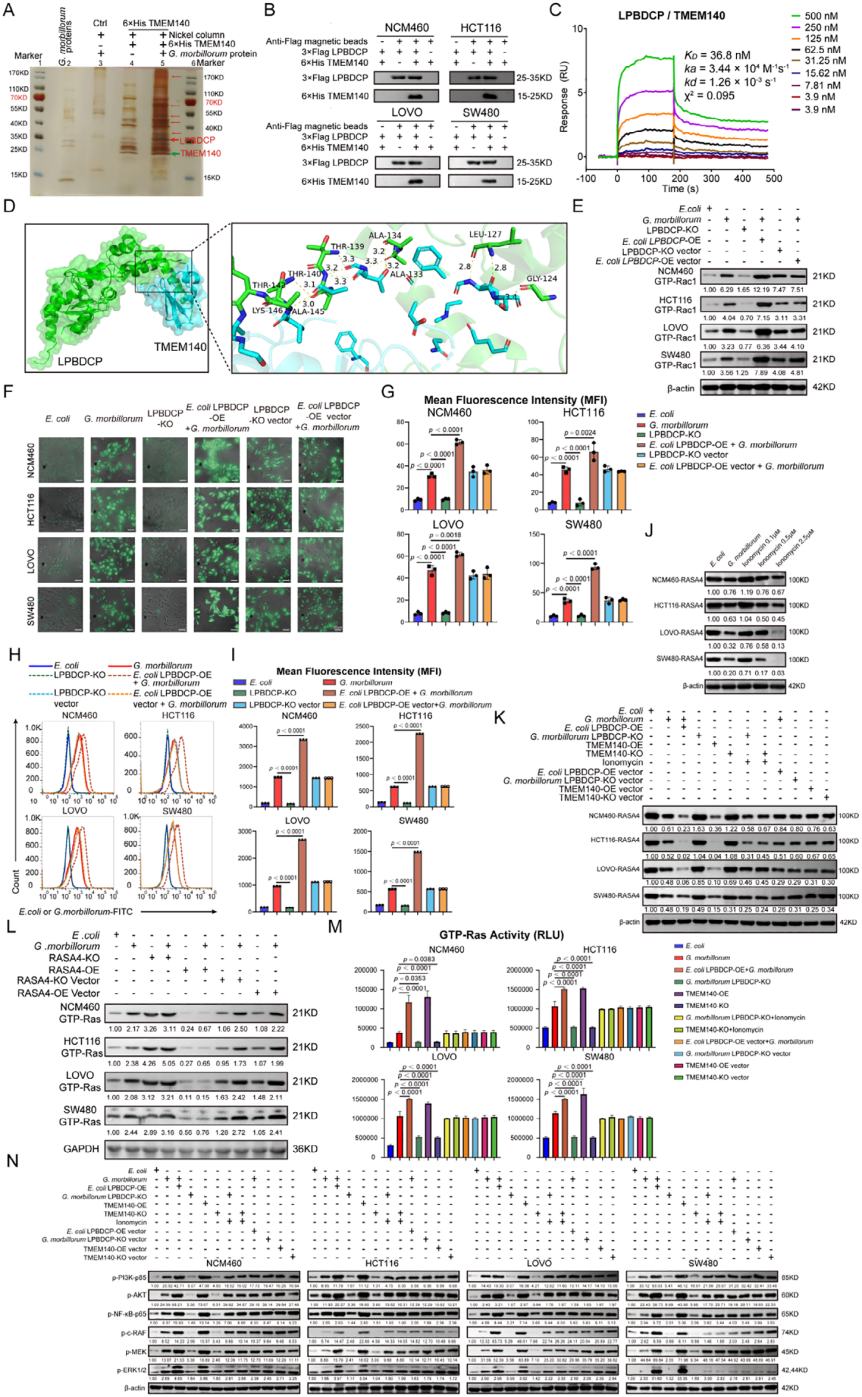
The invasion of cells by *G. morbillorum* is associated with LPBDCP and decreases RASA4 levels for activating the Ras Pathway. (A) The Co‐IP experiment was performed to detect *G. morbillorum* proteins that can bind to TMEM140. The red arrow indicates the *G. morbillorum* protein that can specifically bind to TMEM140. (B) The binding of LPBDCP to TMEM140 was detected by SDS‐PAGE. (C) Surface plasmon resonance (SPR) analysis of the direct interaction between purified His‐LPBDCP‐WT (analyte) and immobilized His‐TMEM140 (ligand). Lines represent different concentrations of LPBDCP. Kinetic parameters (*K*_D_, *Ka*, *Kd*) are indicated. (D) Representative docking pose of the LPBDCP–TMEM140 complex. LPBDCP is shown in green and TMEM140 in cyan (left). (Right) Close‐up view of the binding pocket displaying hydrogen bond interactions (yellow dashed lines) with key residues. The binding free energy is −10.8 kcal/mol. The figure was generated using PyMOL. (E) Western blot was conducted to detect the effects of LPBDCP–KO and LPBDCP–OE on the intracellular GTP‐Rac1. LPBDCP: LysM peptidoglycan‐binding domain‐containing protein; LPBDCP–KO: *G. morbillorum‐lpbdcp‐*knockout; LPBDCP–OE: *E.coli*‐LPBDCP‐overexpression. (F) Representative fluorescence images showing the effects of LPBDCP–KO and LPBDCP–OE on Ca^2^^+^ concentration changes (Fluo‐4 probe, green) mediated by *G. morbillorum* invasion. Images are representative of *n* = 3 biologically independent experiments. (G) Statistical analysis of differences in intracellular Ca^2^^+^ mean fluorescence intensity. (H) Representative images from flow cytometry analysis of the invasion capability of *G. morbillorum* or LPBDCP‐OE MG1655. Representative plots from *n* = 3 biologically independent experiments. (I) Statistical analysis of the differences in invasion capability of *G. morbillorum* or LPBDCP‐OE MG1655. (J) The effects of Ionomycin (calcium ionophore A23187) RASA4 protein levels in cells treated with different concentrations. (K) Western blot analysis was conducted to assess the effect of TMEM140 or LPBDCP on RASA4 protein levels within cells. (L) Western blot analysis of GTP‐Ras levels in RASA4 knockout or overexpressing cells. (M) The ELISA assay was used to detect the effect of TMEM140 or LPBDCP on intracellular active Ras (GTP‐Ras) levels. (N) The effects of TMEM140 and LPBDCP on key molecules mediating pathway activation associated with *G. morbillorum* invasion of cells. Data are presented as mean ± SD. Multi‐group comparisons were performed using one‐way ANOVA followed by Tukey's post‐hoc test. The significance level (α) was set at 0.05 (two‐tailed).

The 6×His‐TMEM140 protein purified via nickel affinity chromatography was subjected to co‐immunoprecipitation (Co‐IP) experiments with the whole proteins obtained from sonicated *G. morbillorum*. Co‐IP experiments identified six bacterial proteins interacting with 6×His‐TMEM140 (Figure 6A), including *G. morbillorum*’s LysM peptidoglycan‐binding domain‐containing protein (LPBDCP) (Figure S5A,B). LPBDCP, a 35–40 kDa surface protein, binds bacterial cell wall proteins. Subsequently, co‐incubation of 3×Flag‐LPBDCP with total proteins from cells overexpressing 6×His‐TMEM140 revealed an interaction between LPBDCP and TMEM140 (Figure 6B). To determine whether LPBDCP directly interacts with TMEM140, we performed surface plasmon resonance (SPR) assays using purified recombinant proteins. LPBDCP exhibited concentration‐dependent binding to immobilized TMEM140 with affinity (*K*_D_ = 36.8 nM) (Figure 6C). To predict the structural basis of this interaction, we performed molecular docking analysis. The model revealed that LPBDCP (green) binds to an extracellular loop of TMEM140 (cyan) through multiple hydrogen bonds (yellow dashed lines), with a calculated binding energy of −10.8 kcal/mol (Figure 6D), indicating a thermodynamically favorable and high‐affinity interaction. Based on molecular docking analysis, we substituted the key leucine residue at position 127 within the LysM domain with alanine (L127A). This residue forms two hydrogen bonds with TMEM140 at a minimal distance. Functional studies demonstrated that the L127A mutant exhibited a 2.6‐fold decrease in binding affinity compared to the wild‐type protein (Figure S6). This indicates that the Leu127 is critical for molecular recognition and further validates the specificity of the LPBDCP‐TMEM140 interaction. Interactions between bacteria and host cells frequently entail cytoskeletal reorganization, concomitant with calcium influx and activation of GTP‐Rac1. Western blot analysis showed that LPBDCP knockout abolished *G. morbillorum*‐induced increases in intracellular GTP‐Rac1 and Ca^2^^+^ levels, while LPBDCP overexpression in engineered *E. coli* MG1655 raised these levels (Figure 6E–G). Flow cytometry assays indicated that LPBDCP knockout in *G. morbillorum* reduced its invasion capacity, whereas LPBDCP overexpression in *E. coli* MG1655 enhanced invasiveness (Figure 6H,I). To quantify the invasive capacity of *G. morbillorum*, we performed gentamicin protection assays to determine bacterial invasion rates following co‐incubation with CRC cell lines for 0.5 h at an MOI of 10. Wild‐type *G. morbillorum* exhibited significant invasive capability across all tested cells (invasion rate: 0.5%–0.7%). However, the LPBDCP knockout strain showed significantly attenuated invasion. Complementation with the pLPBDCP plasmid restored invasion to near wild‐type levels, whereas the empty vector control failed to rescue the phenotype (Figure S7A,B). These results suggest that *G. morbillorum* modulates invasion by binding LPBDCP to TMEM140, affecting intracellular Ca^2^^+^ and GTP‐Rac1 levels.

### 2.9 *Gemella morbillorum* Cellular Invasion Decreases RASA4 Levels and Activates Ras‐RAF‐MEK‐ERK and Ras‐PI3K‐AKT‐NF‐κB Pathways

As shown in the previous results, *G. morbillorum* promotes cell proliferation, inhibits apoptosis, and accelerates the cell cycle in four cell lines. We hypothesized these effects are linked to Ca^2^^+^ elevation and cytoskeletal remodeling. Figure 5A,B highlights RASA4, which may regulate the Ras‐MAPK pathway and is associated with intracellular Ca^2^^+^ levels [21, 22]. To test this hypothesis, we conducted Western blot analyses. The results indicated that exposure to *G. morbillorum* led to decreased RASA4 expression across all four cell lines, with this reduction being significantly amplified by the calcium ionophore A23187 (Ionomycin) (Figure 6J), indicating that RASA4 levels decrease with rising Ca^2^^+^ concentrations. Western blot results confirmed that *G. morbillorum* treatment downregulated RASA4 levels, an effect abolished by TMEM140 or LPBDCP knockdown but enhanced by their overexpression (Figure 6K). RASA4, a Ras GTPase‐activating protein (GAP), converts Ras from its GTP‐bound (active) to GDP‐bound state (inactive), inhibiting Ras signaling [22]. To determine whether reduced RASA4 levels are sufficient to elevate GTP‐Ras, we performed Western blot analysis in cells treated with *G. morbillorum*, RASA4 knockout, and RASA4 overexpression. RASA4 knockout significantly increased GTP‐Ras level, whereas RASA4 overexpression markedly decreased GTP‐Ras level (Figure 6L). Activated Ras triggers the RAF‐MEK‐ERK and PI3K‐AKT‐NF‐κB pathways, which regulate cell proliferation, survival, and metabolism, and can also activate NF‐κB to promote cell survival [23, 24]. Consistently, downstream effectors p‐ERK1/2 and p‐AKT exhibited parallel trends (Figure S8A,B). *G. morbillorum* treatment increased GTP‐Ras levels, an effect enhanced by TMEM140/LPBDCP overexpression but abolished by their knockout, and ionomycin treatment restores GTP‐Ras levels in knockout cells (Figure 6M). Subsequent Western blot experiments further confirmed that *G. morbillorum* treatment upregulated p‐PI3K‐p85, p‐AKT, p‐NF‐κB‐p65, p‐c‐RAF, p‐MEK, and p‐ERK1/2 levels, and the effects were negated by TMEM140 and LPBDCP knockdown but amplified by their overexpression or Ionomycin treatment (Figure 6N). Overall, *G. morbillorum* invasion induced Ca^2^^+^ elevation, lowers RASA4 levels, and activates the Ras‐RAF‐MEK‐ERK and Ras‐PI3K‐AKT‐NF‐κB pathways, exerting tumorigenic effects.

### 2.10 *Gemella morbillorum* Affects the Stability of p53 by Regulating Its Deacetylation via NDPD Secretion

**FIGURE 7:**
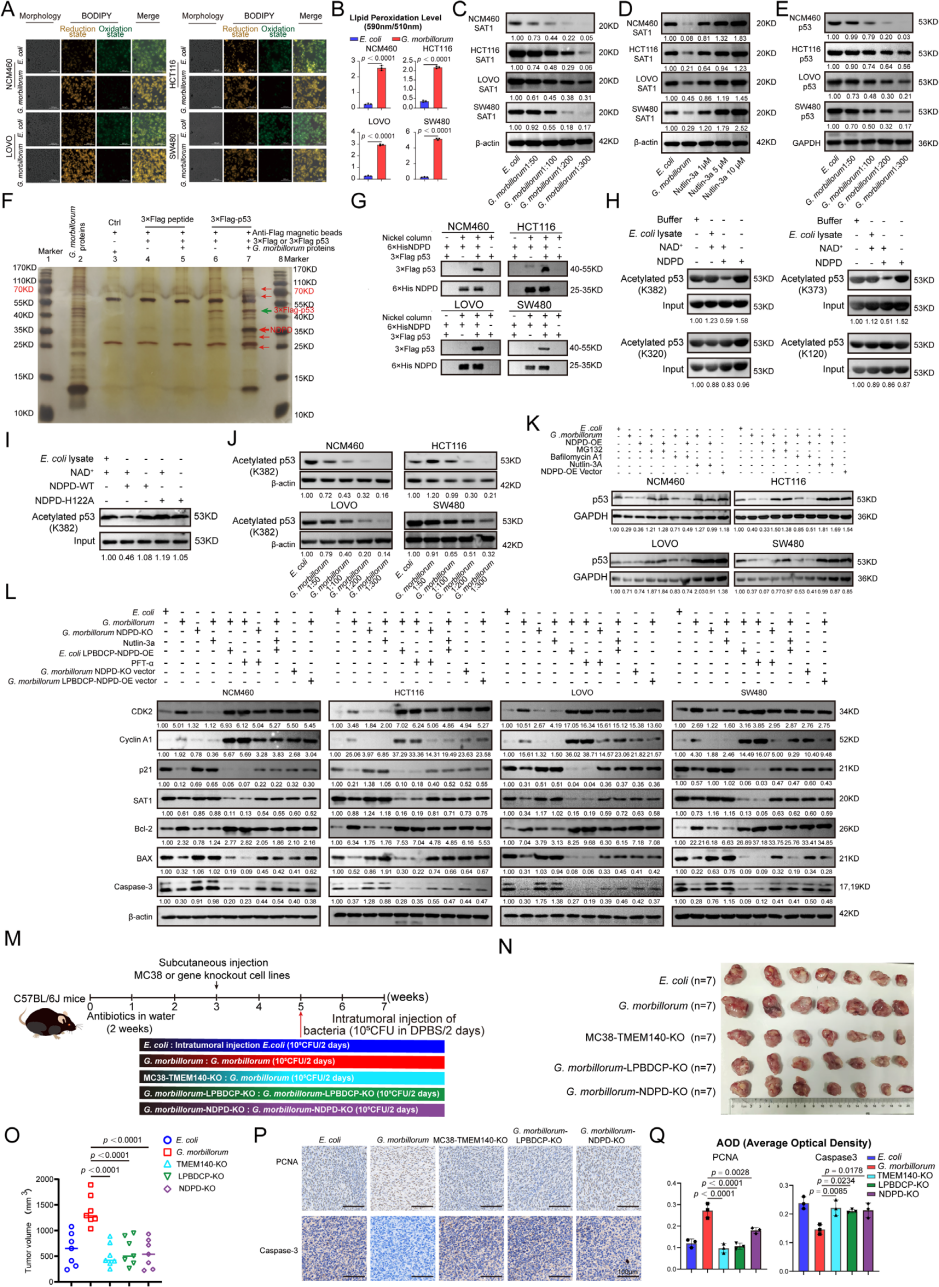
*G. morbillorum* modulates p53 stability by regulating its deacetylation via NDPD secretion. (A) Representative images showing *G. morbillorum* invasion effects on cellular lipid‐derived ROS levels, detected by C11‐BODIPY probe. Orange fluorescence: reduced probe; Green fluorescence: oxidized probe. Images are representative of *n* = 3 biologically independent experiments. (B) Statistical analysis of the ratio of average fluorescence intensity of reduced to oxidized C11‐BODIPY probe, reflecting the degree of ferroptosis in cells. (C) Western blot was used to detect the changes in intracellular SAT1 levels with varying concentrations of *G. morbillorum*. (D) Western blot was used to detect the changes in intracellular SAT1 levels with varying concentrations of the p53 activator Nutlin‐3a. (E) The intracellular p53 levels were analyzed with the concentration of *G. morbillorum*. (F) The Co‐IP experiment was performed to detect *G. morbillorum* proteins that can bind to p53. (G) The binding of NDPD to p53 was detected by SDS‐PAGE. NDPD: NAD‐dependent protein deacetylase. (H) Biochemical screening was performed to identify NDPD‐mediated p53 deacetylation sites. (I) In vitro deacetylation activity of NDPD and its catalytic mutant (H122A) on acetylated p53. (J) The level of acetylated p53 varies with the concentration of *G. morbillorum*. (K) Western blot analysis of NDPD‐mediated p53 deacetylation and its blockade by MG132 and Bafilomycin A1. (L) The impact of various treatments on downstream molecules of p53 affecting the cell cycle (cyclin A1, CDK2, p21), ferroptosis (SAT1), and apoptosis (Bcl‐2, BAX, Caspase‐3). Nutlin‐3a: a p53 agonist; PFT‐α: inhibition of p53. (M) The process of establishing a MC38 subcutaneous tumor model in C57BL/6J mice. (N) Animal experiments were conducted to investigate the impact of key molecules involved in *G. morbillorum* cellular invasion on tumor proliferation (*E. coli* MG1655*, n* = 7; *G. morbillorum*, *n* = 7; MC38‐TMEM140‐KO + *G. morbillorum*, *n* = 7; *G. morbillorum*‐LPBDCP‐KO, *n* = 7; *G. morbillorum*‐NDPD‐KO, *n* = 7). (O) Quantitative analysis chart of differences in tumor volume among different groups of mice. (P) The proliferative capacity (PCNA) and apoptosis levels (Caspase‐3) of tumor cells in different groups of mice were analyzed by IHC. (Q) Statistical analysis of PCNA and Caspase‐3 expression in IHC images of mouse tumor tissues across different treatment groups. Data are presented as mean ± SD. Comparisons between two groups were performed using Student's *t*‐test. Multi‐group comparisons were conducted using one‐way ANOVA followed by Tukey's post‐hoc test. The significance level (α) was set at 0.05 (two‐tailed).

As illustrated in Figure 4C–E, following the invasion of *G. morbillorum* into cells, mitochondrial reactive oxygen species (MitoROS) levels remain unchanged, whereas cellular reactive oxygen species (CellROS) levels decline. This finding is both notable and surprising. Given the downregulation of SAT1, a key ferroptosis mediator regulated by p53 [25], observed in our RNAseq analysis, we believe this is not a coincidental occurrence. Rather, these converging results imply that *G. morbillorum* invasion may modulate cellular ferroptosis levels [26]. Subsequent quantification of lipid peroxidation‐derived ROS (C11‐ROS) confirmed diminished ferroptotic activity in all tested cells (Figure 7A,B). Western blot analysis showed that *G. morbillorum* dose‐dependently inhibited SAT1 (Figure 7C). This regulatory cascade was validated by Nutlin‐3a (a p53 agonist)‐induced upregulation of SAT1 (Figure 7D). Similarly, *G. morbillorum* influences cellular p53 levels in a dose‐dependent fashion (Figure 7E).

We purified 3×Flag‐tagged p53 from cellular lysates using anti‐Flag magnetic beads and studied its interactions with *G. morbillorum* lysates. Co‐IP assays identified five protein bands on SDS‐PAGE (Figure 7F), one of which was NAD‐dependent protein deacetylase (NDPD), a *G. morbillorum* protein with a conserved NAD‐dependent deacetylase domain (Figure S9A,B), and p53 regulation involves NAD‐dependent protein deacetylases [27]. To verify that p53 serves as a specific substrate for NDPD, we first examined the culture supernatant of *G. morbillorum* overexpressing 6×His‐NDPD and confirmed the secretion of NDPD (Figure S9C). Co‐incubation experiments subsequently demonstrated specific binding between NDPD and p53 (Figure 7G).

Human NAD‐dependent deacetylases SIRT1‐–7 exhibit distinct subcellular localizations: SIRT1 (nuclear/cytoplasmic), SIRT2 (cytoplasmic), SIRT3–5 (mitochondrial), and SIRT6/7 (nuclear), all requiring NAD^+^ as an essential co‐substrate [28]. Regarding p53 deacetylation, SIRT1/2 primarily target K382/K373, whereas SIRT7 acts on K320/K373 within the nucleus [29, 30, 31]; no evidence currently supports the involvement of SIRT4 or SIRT5 in this process, and SIRT6 predominantly targets histones. Notably, although K120 and K164 are not classical deacetylation sites, their acetylation status within the DNA‐binding domain is critical for p53 binding to pro‐apoptotic targets (BAX and PUMA) and represents a key determinant of cell fate decisions [32]. To precisely identify the specific deacetylation sites of NDPD on p53, we selected a representative combination of Lys382, Lys373, Lys320, and Lys120, covering structural domain distribution (C‐terminal, DNA‐binding, and tetramerization domains), enzymatic specificity (targets of SIRT1/2, SIRT6, and SIRT7), and functional dimensions (protein stability, transcriptional activity, subcellular localization, and oligomerization), thereby establishing a comprehensive framework linking deacetylation modifications to the multifunctional regulation of p53.

We established an in vitro deacetylation assay utilizing recombinant purified His‐tagged NDPD and acetylation‐enriched His‐tagged p53 (overexpressed in NCM460 cells and pretreated with trichostatin A and nicotinamide). Four experimental groups were designed: buffer control, *E. coli* lysate supplemented with NAD^+^, NDPD with NAD^+^, and NDPD alone (without NAD^+^). Following reaction termination, samples were analyzed by immunoblotting using site‐specific antibodies against acetylated p53 at residues K382, K373, K320, and K120. The results revealed that NDPD significantly diminished acetylation levels at K382 and K373 in an NAD^+^‐dependent manner, while exerting no significant effect on K320 or K120 (Figure 7H). The catalytically inactive NDPD H122A mutant lost its ability to deacetylate p53. In contrast, increasing concentrations of *G. morbillorum* elicited a dose‐dependent decrease in acetylated p53 levels, further corroborating NDPD's deacetylation activity on p53 (Figure 7I,J). Moreover, unlike wild‐type NDPD, the NDPD H122A mutant failed to upregulate phosphorylated CDK2 (p‐CDK2) and downregulate p21, downstream targets of p53 (Figure S10A,B).

P53 degradation is primarily regulated by the MDM2‐mediated ubiquitin‐proteasome pathway (blockable by MG132), while a lysosomal degradation route (inhibitable by Bafilomycin A1) may also contribute [33, 34]. Treatment with MG132 and Bafilomycin A1 demonstrated that NDPD‐mediated p53 deacetylation was specifically blocked by MG132 but not by Bafilomycin A1, and this effect was reversible by Nutlin‐3a (Figure 7K). Collectively, these data establish that NDPD destabilizes p53 through deacetylation. RNA‐seq analysis of *G. morbillorum*‐treated cells revealed no alterations in p53 mRNA levels, indicating that the observed effects involve epigenetic regulation mediated via NDPD‐dependent p53 deacetylation.

Western blot analysis of p53 downstream effectors revealed that *G. morbillorum* treatment upregulated cell cycle‐associated proteins (CDK2, Cyclin A1), downregulated p21, and affected apoptosis/ferroptosis‐related molecules (SAT1, BAX, Caspase‐3, Bcl‐2) (Figure 7L). These effects were abolished by LPBDCP/NDPD knockdown and exacerbated by overexpression. Pharmacological inhibition of p53 with PFT‐α restored the effects, while the p53 agonist Nutlin‐3a neutralized the pro‐survival phenotypes driven by LPBDCP/NDPD overexpression (Figure 7L).

### 2.11 Tumor Promotion and Mechanistic Insights in MC38 Models

In MC38 tumor‐bearing mice, intratumoral bacterial administration following tumor establishment revealed distinct oncogenic effects after 5 weeks. Tumor volume analysis showed that *G. morbillorum*‐driven tumor promotion was abolished by LPBDCP or NDPD knockout in *G. morbillorum* and by TMEM140 depletion in host cells (Figure 7M–O). Immunohistochemistry (IHC) analysis of representative tumors revealed elevated PCNA levels and reduced Caspase‐3 (Figure 7P–Q), suggesting that *G. morbillorum* can promote cell proliferation and inhibit apoptosis. We analyzed the changes in protein levels related to the Ras‐RAF‐MEK‐ERK, Ras‐PI3K‐AKT‐NF‐κB, and p53 pathways in different treatment groups, and results showed that *G. morbillorum* significantly increased the levels of p‐PI3K‐p85, p‐AKT, p‐NF‐κB‐p65, p‐c‐RAF, p‐MEK, and p‐ERK1/2. However, these increases were negated by the knockdown of either TMEM140 or LPBDCP, while NDPD knockdown had nearly no significant effect on this upregulation (Figure S11A). Additionally, *G. morbillorum* markedly altered the levels of key proteins in the p53 downstream pathway, such as CDK2, Cyclin A1, p21, SAT1, Caspase‐3, and Bcl‐2. Nevertheless, these alterations were effectively reversed by the knockdown of TMEM140, LPBDCP, or NDPD (Figure S11B).

## 3 Discussion

This study systematically evaluates the role of *G. morbillorum* in CRC carcinogenesis by detecting its presence in tumor tissues and fecal samples from CRC patients and by investigating its tumorigenesis related cellular effects. We found significant enrichment of *G. morbillorum* in both fecal samples and tumor tissues from CRC patients. Comprehensive in vivo and in vitro experiments established that *G. morbillorum* actively promotes colorectal tumorigenesis by enhancing the proliferation of intestinal epithelial cells and CRC cell lines, shortening the cell cycle duration, and suppressing apoptotic.

Although multiple gut microbiota species have been implicated in oncogenesis, our work provides the first definitive evidence that *Gemella morbillorum* is a CRC‐inducing bacterium, with detailed carcinogenic mechanistic insights. Multiple studies have suggested that *G. morbillorum* may be a key bacterial species associated with CRC. In a comprehensive multi‐omic analysis encompassing 2629 samples from 16 studies, partitioned into 20 case‐control datasets covering 13 disease states, three key bacterial species were identified within the late‐stage CRC‐associated module: *Peptostreptococcus stomatis*, *Peptostreptococcus anaerobius*, and *Gemella morbillorum*. Notably, *Gemella morbillorum* exhibited significantly elevated replication rates across all CRC stages relative to controls, suggesting its role as a pivotal pathobiont engaged from early tumorigenesis and continuously driving disease progression, rather than merely a secondary colonizer in advanced stages [35]. In another meta‐analysis encompassing 3741 fecal metagenomes, *Parvimonas micra* (SMD = 0.63), *Gemella morbillorum* (SMD = 0.59), and *Peptostreptococcus stomatis* (SMD = 0.58) were established as the classical oral‐derived species most strongly associated with colorectal cancer (CRC), while *Fusobacterium nucleatum* (SGB6007, SMD = 0.54), representing newly identified subspecies diversity, ranked immediately after them. Notably, *Gemella morbillorum* exhibited significant enrichment beginning from Stage I, a characteristic that renders it a promising biomarker for early diagnosis [36]. Integrating whole‐genome sequencing data from Genomics England encompassing 8,908 cancer patients—revealing a 39% positivity rate of *Gemella* in colorectal cancer tissues—with spatial profiling studies showing this genus ranks fourth in abundance within the tumor microbiome, collectively underscores the pivotal role of *Gemella* in the colorectal cancer microenvironment. These results suggest that *Gemella morbillorum* likely functions as a key pathogen in CRC. To date, no antagonistic interactions between *G. morbillorum* and other established CRC‐associated bacteria (e.g., *Fusobacterium nucleatum*) have been reported [37, 38].

Our study reveals a multi‐step virulence mechanism employed by *G. morbillorum*: Initial adhesion triggers cytoskeletal remodeling and cellular invasion, followed by intracellular Ca^2^^+^ accumulation, which downregulates RASA4 and alters Ras pathway activity. Concurrently, NDPD secretion modulates p53 deacetylation and its downstream signaling. These findings provide novel insights into the role of *G. morbillorum* in CRC carcinogenesis, revealing unique mechanisms of bacterial invasion and tumorigenesis, which significantly advance our understanding of microbiota‐CRC interactions and highlight the intervention potential of targeting specific microbial components for CRC prevention and treatment.

The CRC‐specific tropism of the *G. morbillorum* invasion mechanism can be rationalized through GTEx data. Although TMEM140 exhibits moderate expression across multiple tissues (ranking within the upper quartile in colon, small intestine, and rectum). Moreover, analysis of IBD cohorts (315 patients vs. 8690 healthy controls) further supports this notion: *G. morbillorum* is also significantly elevated in IBD (27‐fold increase, *p* < 0.001) (Figure S4B). This finding supports its role as an inflammation‐associated pathobiont and further reinforces the specificity of this mechanism for colorectal tumorigenesis.

Research on bacterial adhesion and invasion has been extensively explored, revealing two key mechanisms. One is integrin‐mediated invasion. For example, the two important adhesins of *Fusobacterium nucleatum*, FadA and Fap2, promote invasion by binding to host cell receptors. FadA binds to E‐cadherin on the host cell surface, activating the β‐catenin signaling pathway to promote cell proliferation, and tumorigenesis; Fap2 binds to galactosyl‐galactosamine (Gal‐GalNAc) on the host cell surface to facilitate bacterial invasion [8]. Similarly, the FnBPs of *Staphylococcus aureus* bind to β1 integrin, activating downstream signaling pathways that lead to cytoskeletal rearrangement and invasion [39]. *Listeria monocytogenes* invades by binding InlB to host cell surface receptors, causing membrane wrapping around the bacteria. Likewise, The choline‐binding proteins (CBPs) of *Streptococcus pneumoniae* bind to specific receptors on the surface of host cells, promoting bacterial adhesion and invasion [40, 41]. The other mechanism involves disrupting tight junctions and adherens junctions to invade cells. For instance, *Helicobacter pylori* lipopolysaccharide activates Toll‐like receptor 2 (TLR2) on the surface of gastric mucosa, inducing Ca^2+^ influx, which mediates the proteolytic cleavage of E‐cadherin by Ca^2^^+^ dependent cysteine proteases, ultimately leading to the dissociation of adherens junctions [42]. In this study, *G. morbillorum*, a Gram‐positive *Firmicutes* bacterium similar to *Staphylococcus aureus* and *Listeria monocytogenes*, was shown to adhere to and invade cells via a mechanism distinct from macrophage phagocytosis, which relies on TLR4 signaling. *G. morbillorum* utilizes its LPBDCP to bind to TMEM140 on the host cell surface. This interaction promotes bacterial adhesion, cytoskeletal remodeling, and subsequent cell invasion. This finding represents a novel mechanism of bacterial cell invasion.

During bacterial adhesion and invasion, cytoskeletal remodeling and changes in various downstream signaling molecules often occur. Cytoskeletal remodeling is frequently accompanied by Ca^2+^ influx, which may be one mechanism driving this process. For example, *Helicobacter pylori* induces Ca^2^^+^ influx in gastric mucosa. Likewise, *Listeria monocytogenes* secretes Listeriolysin O (LLO) to form ion‐permeable pores in host cell membranes. Ca^2^^+^ influx also occurs when macrophages phagocytose bacteria [17, 42, 43]. Although Ca^2^^+^ influx is not definitively or specifically linked to bacterial invasion, Ca^2+^ are essential intracellular signaling molecules that can influence multiple signaling pathways [44]. In our study, we observed Ca^2^^+^ influx. A key finding was that the transient Ca^2^^+^ influx during bacterial invasion not only affects cytoskeletal remodeling but also leads to decreased levels of RASA4. Both RASA4 and Ca^2^^+^ can modulate the Ras downstream pathway [45, 46], with complex interplay between them. Our study confirmed that Ca^2^^+^ influx induced by bacterial invasion downregulates RASA4 and affects the Ras signaling pathway (Figure 6). The downregulation of RASA4 results in decreased levels of active Ras (GTP‐Ras), thereby activating the PI3K‐AKT‐NF‐κB and RAF‐MEK‐ERK pathways. This provides a new explanation for the tumorigenic effects caused by bacterial invasion.

P53 plays a crucial role in tumorigenesis and cancer progression, with over 50% of human cancers exhibiting p53 inactivation due to mutations. p53 and the gut microbiota can influence each other. For example, The metabolite isoamylamine (IAA) of the *Ruminococcaceae* promotes apoptosis of microglial cells by recruiting p53 to the S100A8 promoter region [47]. Conversely, p53 can shape the gut microbiota by regulating the host's innate immune system. p53 can lead to excessive apoptosis of intestinal epithelial cells (IEC), disrupt the gut barrier, and facilitate the colonization of harmful bacteria [48]. Moreover, the gut microbiota can transform mutant p53 from a tumor‐suppressive state to an oncogenic state [49]. Our study provides a new explanation for how the gut microbiota affects p53. MDM2 is a key E3 ubiquitin ligase that regulates p53 stability in the cytoplasm. MDM2 binds to p53 and promotes its polyubiquitination, leading to p53 degradation via the 26S proteasome pathway. In addition to MDM2, deubiquitinases, such as USP10, USP4, and HAUSP, as well as kinases like ATM and acetyltransferases like p300 and PCAF, can modify p53 to influence its stability. Acetylation and ubiquitination are competitive processes, with overlapping sites, and acetylation can stabilize p53 by inhibiting MDM2‐mediated ubiquitination. Moreover, acetylation and ubiquitination both influence p53's subcellular localization. Acetylated p53 tends to stay in the nucleus, enhancing its transcription factor activity. Conversely, ubiquitinated p53 is often transported to the cytoplasm, reducing its nuclear presence and transcriptional activity [49, 50, 51, 52, 53]. *G. morbillorum* can affect p53 deacetylation through NDPD, which may increase p53 phosphorylation levels and enhance MDM2 mediated ubiquitination, thereby affecting p53 stability. It is still unclear whether *G. morbillorum* secretes NDPD after escaping into the cytoplasm and continuing to survive there, similar to how *Listeria monocytogenes* secretes listeriolysin O and phospholipases to disrupt the phagosome membrane [8], or whether NDPD secretion by *G. morbillorum* is triggered by stress during cell invasion. Further mechanistic studies are needed to elucidate these possibilities.

These findings raise a mechanistic question: do Ras‐pathway activation and p53‐inactivation act independently, or synergistically? We propose the latter. A previous study established a direct link between the p53 and Ras pathways, demonstrating that p53 induces ATF3 expression, which directly binds promoters of Ras downstream genes (*CXCL1*, *IL‐1β*, *MMP3*) to repress their transcription and dampen Ras‐MAPK/PI3K‐AKT signaling output [54]. Conversely, oncogenic K‐Ras activates the PI3K‐AKT pathway, which subsequently phosphorylates MDM2 at Ser183 to enhance its stability and promote p53 degradation [55]. Finally, mechanistic studies indicate that wild‐type p53 suppresses NF‐κB activation—a critical downstream effector of Ras‐MAPK/PI3K‐AKT signaling—suggesting that p53 status governs the magnitude of Ras‐induced inflammatory signals [56, 57]. This principle extends to our model, wherein NDPD‐mediated p53 loss derepresses Ras‐driven NF‐κB activation, thereby amplifying the inflammatory microenvironment that promotes CRC progression.

We acknowledge the following limitations of this study. As an exploratory single‐center pilot study conducted at Tongji Hospital between 2020 and 2022, our clinical cohort comprised a relatively small sample size (14 CRC tumor tissues and 20 peritumoral tissues), and detailed clinical metadata were not systematically collected. Specifically, due to the retrospective nature of tissue sample collection, comprehensive clinical metadata—including age, sex, BMI, tumor stage, and medication history—were not available, precluding the generation of a detailed patient characteristics summary table. To contextualize these clinical observations, we leveraged the TCGA dataset (*n* = 622 CRC vs. *n* = 51 controls) as an independent validation cohort with well‐characterized clinical annotations. To address these limitations, we utilized an independent large‐scale dataset for external validation, combined with multi‐center fecal metagenomic data and mechanistic experiments using confocal microscopy and TEM, which collectively reinforce the robustness of our conclusions.

Additionally, while metagenomic analysis in antibiotic‐pretreated SPF mice confirmed *G. morbillorum* colonization, we acknowledge that antibiotic depletion cannot completely eliminate residual microbiota or exclude potential contributions from microbiome alterations. Future studies utilizing germ‐free (GF) mouse models will be essential to conclusively demonstrate microbiota‐independent effects.

While the present study primarily elucidates epithelial cell‐intrinsic mechanisms, our findings suggest that *G. morbillorum*‐induced PI3K‐AKT‐NF‐κB activation (Figure 6) may extend beyond epithelial transformation to modulate the tumor immune microenvironment. PI3K‐AKT signaling is well‐established to maintain regulatory T cell (Treg) proliferation and function, and to drive macrophage polarization toward the immunosuppressive M2 phenotype via the mTOR axis. Thus, *G. morbillorum*‐mediated pathway activation likely facilitates immune evasion and colorectal tumorigenesis by establishing an immunosuppressive niche. Future investigations into the bacterium's role in immune microenvironment remodeling will complement the mechanistic foundation established herein [58, 59].

Building upon our identification of *G. morbillorum* as a novel pathogen driving CRC progression through the LPBDCP‐TMEM140‐p53/Ras axis, future research should prioritize developing targeted therapeutics and diagnostic tools for early detection and personalized intervention, validating these mechanisms in advanced preclinical models, such as genetically engineered mice or patient‐derived xenografts, and ultimately conducting clinical trials investigating antimicrobial therapies targeting this bacterium or its downstream signaling pathways. These efforts may extend beyond CRC to other microbiota‐driven malignancies, highlighting the broad role of tissue‐invasive bacteria in cancer biology and paving the way for clinical translation.

## 4 Conclusion

In conclusion, our study shows that *G. morbillorum* promotes colorectal tumorigenesis by enhancing cells proliferation, inhibiting apoptosis and ferroptosis, and accelerating cells cycle progression. Mechanistically, *G. morbillorum* employs a multi‐step pathogenic strategy involving bacterial invasion via LPBDCP‐TMEM140 interaction, intracellular Ca^2^^+^ influx, RASA4 downregulation, activation of the Ras pathway, and p53 destabilization through NDPD‐mediated deacetylation. These findings highlight *G. morbillorum* as a novel CRC associated pathogen and underscore its potential as a therapeutic target for CRC prevention and treatment.

## 5 Experimental Section

### 5.1 In‐house Participant Recruitment and Sample Collection

Two in‐house cohorts (CHN_WH1 and CHN_WH2) including 60 patients with colorectal adenoma (CRA), 100 patients with CRC, and 85 healthy individuals were recruited from 2019 to 2022 for fecal metagenomic sequencing (Table S1). Detailed inclusion/exclusion criteria and biospecimen protocols appear in the Section 5.3. Our work was approved for research ethics by the Ethics Committee of Tongji Medical College, Huazhong University of Science and Technology (approval code: 2021 IEC‐A189), and written informed consent was obtained from all participants/patients.

### 5.2 Public Metagenomic Datasets of Patients With CRA or CRC and Controls

PubMed‐retrieved metagenomic datasets (2014–2023) encompassed 11 studies (246 CRA, 885 CRC, 903 controls; Table S2). Raw sequencing data were acquired from the European Nucleotide Archive (ENA)/the DNA Data Bank of Japan (DDBJ) via accession numbers (Table S2). Metadata integration combined supporting materials from original publications, the R package “curatedMetagnomicData,” and public databases (e.g., NCBI biosample, ENA, and National Omics Data Encyclopedia (NODE)).

### 5.3 Sample Collection and Storage Protocol

We recruited patients with CRA or CRC, and healthy individuals undergoing physical examination as the study participants from 2019 to 2022. The study included patients aged 18–80 with a pathologically confirmed initial diagnosis of CRA or CRC. The control group comprised patients of the same age range who were confirmed to have neither CRA nor CRC at the same hospital. The exclusion criteria were: (1) non‐primary tumor; (2) with histories of tumor or inflammatory bowel disease (IBD); (3) had used antibiotics or probiotic preparations within the past 3 months; (4) had undergone gastrointestinal surgery within the past 3 months; (5) had undergone radiotherapy or chemotherapy; (6) had received colonoscopy within 1 month [60]. Participants were instructed to self‐collect about 5 gm of fresh stool using a sterile stool specimen cup. Participants were also prompted to avoid blood and urine contamination during stool collection and to collect samples from the middle and posterior segments as close to the interior of the stool as possible. Stool samples were collected from the participants before colonoscopy and frozen in a −80°C refrigerator within 4 h for subsequent metagenomic sequencing.

### 5.4 DNA Extraction, Processing, and Sequencing

Total DNA was extracted from fecal samples by Magnetic Soil and Stool DNA Kit (TIANGEN, DP712, Beijing) according to the manufacturer's manual. DNA samples with a total amount of DNA ≥ 1 µg, a concentration greater than 30 ng/µL, and an OD260/OD280 range between 1.8 and 2.0 were considered qualified samples. Subsequently, qualified DNA samples were processed through the steps of DNA ultrasonic fragmentation, DNA end repair and adenylation, adapter ligation, magnetic bead purification, and PCR amplification. Processed DNA samples were used to construct metagenomic libraries using NEBNext Ultra II FS DNA Library Prep Kit for Illumina (New England Biolabs, NEB #E7805), and the library was quantified using KAPA Library Quant (Illumina) DNA Standards&Primer Premix (Kit Code KK4824). After library quality control steps, such as dilution, qPCR reaction, and standard assay, qPCR concentration ≥ 1.5 nM was defined as a qualified library. The qualified libraries were sequenced using the Illumina NovaSeq 6000 sequencing platform.

### 5.5 Metagenomic Sequencing Data Processing

Fastp (version 0.23.2) software was used to remove low‐quality data for the raw sequencing reads (including primer sequences, adapter sequences, reads with average quality of less than 20, reads with a base quality of one window less than 15 based on a 4‐base sliding window algorithm, reads with more than 5 N bases or reads with lengths less than 50 bp) so that high quality reads were got. Nine mammalian genomes (hg38, bosTau9, canFam6, felCat9, galGal6, mm39, rn6, susScr11, rheMac10, accessed on Feb 2023, National Center for Biotechnology Information (NCBI) genomes), 48 913 bacterial plasmids (NCBI RefSeq database accessed on Feb 2023), 10 389 complete plastids (NCBI RefSeq database accessed on Feb 2023), 14 381 mitochondrion genomes (NCBI RefSeq database accessed on Feb 2023), and 6093 UniVec sequences (NCBI RefSeq database accessed on Feb 2023), which are potential habitat/laboratory‐associated or extra‐chromosomal sequence contaminants [61, 62, 63], were constructed as an indexed database using bowtie2‐build function of Bowtie2 software (version 2.4.5) [64, 65]. Then high‐quality reads that could be mapped to the indexed database were discarded to get clean reads using bowtie2 with “very‐sensitive” default settings. In‐house cohort fecal samples underwent metagenomic library preparation followed by shotgun sequencing on Illumina NovaSeq 6000 (12 Gb depth/sample, paired‐end). Raw data processing generated quality‐controlled reads for microbial annotation.

### 5.6 Microbial Taxonomic Annotation and Analysis

The raw sequencing reads were processed using Fastp (version 0.23.2; RRID:SCR_016962) to remove low quality data. Subsequently, Bowtie2 software (version 2.4.5) was employed to discard high quality reads that mapped to indexed databases containing potential contaminants, yielding clean reads. A custom database encompassing 11 294 bacterial, 24 117 viral, and 1069 archaeal RefSeq genomes sourced from NCBI (May 2023), alongside 5264 fungal genomes from NCBI (RefSeq and GenBank), FungiDB (May 2023), and Ensemble (May 2023), was constructed using Kraken2‐build (version 2.1.2) with default settings. Kraken2 was then utilized to generate taxonomic profiles for all metagenomic sequencing data based on these clean reads.

### 5.7 Screening out of Low‐Abundance Species

The species abundance information obtained during the species annotation stage was processed for further species difference analysis. Specifically, the low‐abundance species of each microbial kingdom were removed. The low‐abundance species were defined as those with an average relative abundance of ≤ 0.001% across all samples. A more stringent criterion was used for bacteria, i.e., unclassified bacteria were excluded from subsequent analyses. Unclassified bacteria refer to species with “sp.” in their taxonomic names, such as Clostridium sp. JN‐9, which is classified as unclassified Clostridium in the phylogenetic lineage of the NCBI taxonomy database (https://www.ncbi.nlm.nih.gov/taxonomy).

### 5.8 Analysis of Microbial Diversity and Identification of Differential Microbial Species

Before conducting inter‐group species difference analysis, the low‐abundance species of each microbial kingdom (bacteria, fungi, viruses, and archaea) were first removed (details in the Supporting Methods). Then, based on the absolute abundance of the screened species, the relative abundance of each microbial kingdom was calculated. We referred to the method by Lin et al. [66]. to reduce the impact of batch effects of different metagenomic datasets. The relative abundance of each species was normalized by dividing by the median of the control group. Based on the standardized species abundance table, the Wilcoxon rank‐sum test was used for intergroup comparison, and the Benjamini–Hochberg (BH) method was used to adjust the *p*‐value of Wilcoxon rank‐sum test. Additionally, the multiple median fold change (MultMedFC, abbreviated as “mFC”) for each species was calculated to identify differential species and the direction of change for these differential species. BH‐adjusted P (Padj) values < 0.05 were considered intergroup difference. An mFC value greater than 1 indicates the enrichment in the group with higher malignancy.

### 5.9 Construction and Validation of Microbial Diagnostic Models

The 1729 metagenomic samples that passed the sample screening rule were divided into discovery and validation (Table S3) datasets according to the different source regions of the datasets to which they belonged, according to the principle of including as many different regions as possible in both discovery and validation sets. This study constructed multiple kingdom microbial diagnostic models for CRC. Microbial diagnostic markers were selected as microbial classifiers based on the random forest algorithm. The average value of the error curves of 100 cross‐validations (10×10 folds) was taken, and the minimum error value plus one standard deviation was used as the cutoff value. The set of species with the minimum number of errors on the error curve less than the cutoff value was used as the optimal feature set for constructing the model. Then, the microbial feature set with the least number of features was selected as the optimal feature set according to the cutoff value [60]. The mean decrease accuracy (MDA) of gut microbial markers in the models was calculated to evaluate the importance of different microorganisms in the optimal feature set to the model. The analysis was completed using the R package random Forest. The area under the receiver operating characteristic curve (AUC‐ROC curve, hereinafter referred to as “AUC”) were used to evaluate the performance of the diagnostic models using the R package pROC. The diagnostic models of gut microbial markers constructed by the discovery set were further validated in the validation datasets.

### 5.10 Co‐Occurrence Analysis of Multi‐Kingdom Microorganisms

FastSpar (Version 1.0.0) software [67] was used to construct microbial interaction networks based on the Sparse Correlations for Compositional data (SparCC) algorithm [68] to study the associations among multi‐kingdom microbial species. The software was run with 100 interactions, and 1000 bootstrap abundance matrices were generated to infer the correlation of each bootstrap count and calculate *p* values based on these correlations. *P* values were adjusted using the BH method. All microbial diagnostic markers included in the final diagnostic models were selected for co‐abundance analysis, and the correlations with a BH‐adjusted *p* value <0.05 were displayed using Cytoscape software (v3.9.1; RRID:SCR_003032).

### 5.11 Procurement and Cultivation of Bacterial Strains

*Gemella morbillorum* (*G. morbillorum*, ATCC 27824, DSM 20572) and *Escherichia coli* MG1655 (ATCC 700926, CGMCC 1.15307) were obtained from the German Collection of Microorganisms and Cell Cultures (DSMZ) and the China General Microbiological Culture Collection Center (CGMCC), respectively. Both strains were anaerobically cultured in modified PYG medium at 37°C. Bacterial growth was monitored at OD_600_ every 6 h. Mid‐log phase cultures (OD_600_ = 0.5) were centrifuged (3500 × g, 10 min) to obtain cell pellets, which were resuspended in DPBS to achieve 10^8^ CFU/mL suspensions. The clarified supernatant was sterile‐filtered through a 0.22 µm filter and added to the cell culture medium at a 1:500 (v/v) ratio for downstream applications.

### 5.12 Procurement and Cultivation of Cell Lines

Human normal colorectal epithelial cells (NCM460, CC‐Y1550) were purchased from Shanghai EK‐Bioscience Biotechnology and three CRC cell lines (HCT116, RRID: CVCL_0291; LOVO, CCL‐229; SW480, RRID: CVCL_0546) were obtained from ATCC. Cells were cultured in DMEM with 10% FBS and 1% penicillin‐streptomycin at 37°C in 5% CO_2_. At 70%–90% confluence, cells were harvested and seeded in 6 well plates at 1.2×10^5^ cells per well. Three experimental conditions were applied: (1) bacterial challenge (MOI: 50, 100, 200); (2) bacterial supernatant addition (1:20, 1:40, 1:50, v/v); (3) anaerobic co‐culture for 1.5 h per intervention, followed by medium replacement. The protocol was repeated daily for three days before endpoint analyses.

Cell line authentication: The NCM460, HCT116, LOVO, and SW480 cell lines used in this study were all authenticated by short tandem repeat (STR) profiling, with certificates provided in the Supporting Information to ensure accurate cell identity. All cell lines were verified to be free of mycoplasma contamination prior to experimentation to ensure the reliability of our results (Figure S12).

### 5.13 Mouse Models

#### 5.13.1 AOM‐Induced Mouse Model of Colorectal Cancer

Six‐week‐old male C57BL/6J (RRID: IMSR_JAX:000664) mice were acclimated in SPF conditions for 14 days and then randomly assigned to four groups (*n* = 10/group): blank, control, *E. coli* MG1655 challenged, and *G. morbillorum* exposed. Experimental groups (control, *E. coli* MG1655 group, and *G. morbillorum* group) received a broad‐spectrum antibiotic cocktail in drinking water, and the blank group was administered an equal volume of PBS for 14 days, with fecal sampling every 48 h. After confirming pathogen clearance by fecal qPCR, all mice of experimental groups (control, *E. coli* MG1655 group, and *G. morbillorum* group) were administered Azoxymethane (AOM, 15 mg/kg, i.p.). Two weeks later, *E. coli* MG1655 group and *G. morbillorum* group received bi‐daily oral gavage with *E. coli* MG1655 or *G. morbillorum* (10^8^ CFU), and the control group received bi‐daily oral gavage sterile medium for 12 weeks, with continued fecal sampling. At week 12, mice were sacrificed to assess colorectal tumor burden.

In our study, we utilized a double‐blind approach to reduce potential bias. Animal allocation to treatment and control groups was managed by a researcher not involved in outcome evaluation, ensuring group assignments were kept confidential through coding. A separate technician, ignorant of the group assignments, handled treatment administration, and routine care. Observations and data collection were performed by a third party, also blind to the groupings. Data analysis was conducted blindly, with group identities disclosed solely after finalizing the primary outcome analysis.

#### 5.13.2 Tumor‐Bearing Mouse Model in C57BL/6J Mice

In the C57BL/6J (RRID: IMSR_JAX:000664) tumor‐bearing mouse model, 6‐week‐old male mice had their gut bacteria cleared by the aforementioned antibiotic cocktail for 2 weeks. Subsequently, wild‐type or target gene knockout MC38 (RRID:CVCL_B288) cell lines were subcutaneously inoculated. Two weeks later, tumors were intratumorally injected with *G. morbillorum* or *E. coli* MG1655. Fresh bacterial suspensions with an OD_600_ of 0.5 were centrifuged and resuspended in DPBS to achieve a final concentration of 10^8^ CFU/mL. Bacteria were injected at three equidistant points in the center and periphery of the tumor, with a total bacterial dose of 10^5^ CFU. Injections were administered every two days for a total duration of 2 weeks, after which the experiment was terminated.

### 5.14 Construction of Overexpression Cell Lines

Total RNA was extracted from each target cell line and used for cDNA synthesis, following the protocol provided by the manufacturer (Thermo Scientific, Cat. No. K1622). Primers were designed based on the mRNA sequence of the target gene, with the upstream primer containing a Kozak sequence (GCCACC) and the start codon ATG to ensure proper translation initiation (see Table S4 for primers information). The target gene cDNA was amplified by PCR, with BamHI and XhoI restriction sites introduced at the ends. The PCR product was separated by 1% agarose gel electrophoresis and the desired fragment was purified and recovered. The purified fragment was then digested with BamHI and XhoI, followed by ligation with the pEGFP‐N1 (RRID:Addgene_172284) vector using T4 ligase. The ligation product was transformed into competent *E. coli* DH5α cells. The target plasmid was extracted from the bacterial culture and sequenced using vector‐specific primers to confirm the introduction of the target gene. The plasmid was linearized by ScaI digestion. The linearized plasmid was then transfected into the CRC cell line using Lipofectamine 2000 (Thermo Scientific, Cat. No. 11668030). The transfected cells were cultured for 48 h, then selected in medium containing G418 (concentration pre‐optimized for the specific cell line, typically 400–800 µg/mL). Clones were picked under an inverted microscope and transferred to a well of a 24‐well plate, then expanded to 6‐well plates for further verification. Western blot analysis was performed to detect whether the target protein was successfully overexpressed, with empty vector pEGFP‐N1 transfected cells serving as negative controls. See Figure S13 for details.

### 5.15 Construction of Knockout Cell Lines for TMEM140

CRISPR‐Cas9 target sequences and sgRNAs were designed using the Synthego tool. Oligonucleotides with 5′‐CACCG and 5′‐AAAC overhangs were synthesized (see Table S 4 for primers information), annealed, and cloned into a BsmBI‐linearized LentiCRISPRv2 vector, which was then transformed into Stbl3 cells for cloning the recombinant plasmids. HEK293T (RRID:CVCL_0063) cells were transfected with the LentiCRISPRv2, psPAX2, and pMD2.G plasmids using Lipofectamine 2000. Viral supernatants were collected 48 and 72 h post‐transfection, filtered through a 0.45 µm membrane, and used to transduce target cells in the presence of 8 µg/mL polybrene. Transduced cells were selected with puromycin (2 µg/mL) for 7–10 days and validated by PCR amplification of the genomic target region followed by Sanger sequencing. See Figure S14 for details.

### 5.16 Construction of Gene Knockout Bacterial Strains

*G. morbillorum* strains in log phase (OD_600_ = 0.5) were electroporated with the pMADΔ*lpbdcp* plasmid (2.0 kV, 25 µF, 200 Ω). Immediately after electroporation, 1 mL pre‐warmed liquid medium was added, and cells were recovered at 30°C for 2 h with gentle shaking (180 rpm). The recovered cells were then plated on agar plates containing erythromycin (5 µg/mL) and X‐Gal (50 µg/mL), followed by incubation at 30°C (permissive temperature) for 24–48 h. Blue colonies, indicating successful plasmid uptake, were picked and re‐streaked onto fresh agar plates with erythromycin and X‐Gal, then cultured at 39°C (non‐permissive temperature) for 12–16 h to select for single‐crossover integrants (plasmid integrated into the chromosome).

To promote the second recombination event and plasmid excision, integrant colonies were diluted in sterile PBS and plated on agar plates containing X‐Gal (50 µg/mL) but without erythromycin, followed by incubation at 30°C for 48 h. White colonies (indicating loss of the temperature‐sensitive plasmid) were picked as candidate knockout strains, while blue colonies represented either wild‐type revertants or unstable integrants. Candidate white colonies were expanded in liquid medium, and genomic DNA was extracted for PCR verification using primers flanking the target region. Sanger sequencing of the PCR products confirmed the gene deletion and sequence integrity. Confirmed knockout strains were preserved in liquid medium containing 20% glycerol at −80°C. The process is shown in Figure S15.

### 5.17 Inducible Expression of Target Proteins in *E. coli* MG1655

The pBAD24 vector was used for cloning. NcoI and XhoI restriction sites were introduced at both ends of the target gene by PCR. The amplicon was verified by 1% agarose gel electrophoresis and purified by gel extraction. The PCR product and vector were digested with NcoI and XhoI, ligated at a 3:1 molar ratio (insert:vector), and transformed into *E. coli* DH5α competent cells. Transformants were plated on LB agar containing ampicillin (100 µg/mL). Positive colonies were verified by colony PCR and sequencing, and the recombinant plasmid was subsequently transformed into *E. coli* MG1655. Preparation of MG1655 chemically competent cells: Cells were grown to log phase (OD_600_ = 0.4–0.6) and prepared using the CaCl_2_ method. Aliquots (100 µL) were stored at 4°C for 12–24 h. For transformation, 100 µL of competent cells were mixed with recombinant plasmid, subjected to heat shock, and plated on LB agar containing ampicillin (100 µg/mL), followed by incubation at 37°C for 12–16 h. Single colonies were inoculated into LB medium containing ampicillin (100 µg/mL) and 0.2% glucose (to suppress basal expression) and cultured overnight at 37°C. The culture was diluted 1:100 into fresh medium and grown to OD_600_ = 0.4–0.6. Protein expression was induced by adding L‐arabinose (0.02%–0.2%) and incubating at 37°C for 4–6 h. Cells were harvested, washed with PBS, and resuspended in lysis buffer (50 mM NaH_2_PO_4_, 300 mM NaCl, 10 mM imidazole, pH 8.0). Cell lysis was performed by sonication (300 W, 5 s on/10 s off, total 7 min) on ice. The lysate was centrifuged to separate the supernatant (soluble protein) from the pellet (inclusion bodies). Total cell lysate, supernatant, and pellet fractions were analyzed by SDS‐PAGE (12% gel) and Western blotting using an anti‐His tag antibody. The materials used are shown in Figure S16.

### 5.18 Fluorescence In Situ Hybridization

The sections were deparaffinized in xylene for 15 min (repeated once) and dehydrated through graded ethanol solutions (95%, 80%, 70%). The *G. morbillorum*‐specific probe (AAGTTTTTCTGGTGCTTGCACTAG) was labeled with FAM (green), and the universal bacterial probe (GCTGCCTCCCGTAGGAGT) was labeled with TAMRA (red) as previously described. After PBS washing and Triton X‐100 permeabilization, sections were hybridized overnight in a pre‐warmed solution containing 200 ng of the respective probe. The hybridization solution consisted of 40 mmol/L Tris HCl, 1.8 mmol/L NaCl, and 0.5% SDS. Post‐hybridization, sections were washed in SDS‐free hybridization solution and rinsed three times with PBS before mounting.

### 5.19 Cell Viability Assessed by CCK‐8 Assay

In a 96 well plate, 100 mL of cell suspension containing 2000 cells per well were added. Subsequently, *G. morbillorum* bacterial suspension at a concentration of 10^8^ CFU/mL was added at a multiplicity of infection (MOI) of 200. The cells and bacteria were co‐incubated under anaerobic conditions for 1.5 h, and this intervention was repeated for three consecutive days. After the bacterial intervention, 10 mL of CCK‐8 solution (Beyotime, Cat. No. C0037) was added to each well. The plate was then incubated in a cell culture incubator for an additional 2 h. Finally, the absorbance was measured at 450 nm.

### 5.20 Colony Formation Assay

Cell suspensions were plated into 6 well plates at a density of 1000 cells per well, with three replicates for each group. After cell adhesion, either *G. morbillorum* or *E. coli* MG1655 was introduced at a MOI of 200 and co‐cultured under anaerobic conditions for 1.5 h. Gentamicin was then applied to eliminate the bacteria, and the cells were cultured at 37°C with 5% CO_2_. The intervention was performed every 4 days, for a total of three times. The plates were subsequently incubated in a 37°C, 5% CO_2_ cell culture incubator for an additional 15–20 days, with the culture medium being changed every 2–3 days until visible colonies formed. The cells were gently washed with PBS, fixed with 4% paraformaldehyde at room temperature for 30 min, and the fixative was discarded. Cells were stained with an appropriate amount of crystal violet solution at room temperature for 15 min. After PBS washing to remove unbound dye, colonies in each well were observed and counted using a microscope.

### 5.21 Immunofluorescence Detection of Ki‐67

*G. morbillorum* or *E. coli* MG1655 was co‐cultured with cells anaerobically for 1.5 h daily over 3 consecutive days. After each co‐culture, bacteria were removed, and cells were cultured at 37°C with 5% CO_2_. Cells were then trypsinized, centrifuged at 1000×g for 5 min, washed with PBS, and fixed with 4% paraformaldehyde for 15 min. After blocking with 3% BSA for 30 min, washed three times with TBS‐T, and incubated overnight at 4°C with rabbit anti‐human Ki67 (1:100). Following 3–5‐min washes in TBS+0.1% Tween‐20, cells were incubated with Alexa Fluor‐488‐conjugated goat anti‐rabbit (1:500) for 30 min at room temperature, counterstained with DAPI, and observed under a fluorescence microscope.

### 5.22 Cell Apoptosis Assessed by Flow Cytometry

Following cell seeding and adherence, either *G. morbillorum* or *E. coli* MG1655 was introduced at a MOI of 200 and co‐cultured anaerobically for 1.5 h. Gentamicin was then applied to eradicate the bacteria, followed by further incubation of the cells at 37°C with 5% CO_2_ for 3 consecutive days. Cells were then digested with EDTA‐free trypsin; gentle handling was applied and the digestion was promptly terminated to avoid cell damage or over‐digestion. The cells were centrifuged at 1000×g for 5 min, and the supernatant was discarded. The cell pellet was washed once with PBS, centrifuged again, and subsequently resuspended in 100 µL of 1× Binding Buffer (10 mM HEPES, 140 mM NaCl, 2.5 mM CaCl_2_, pH 7.4). Next, 5 µL of Annexin V‐FITC and 10 µL of PI staining solution were added, and the mixture was gently vortexed. The cells were incubated at room temperature in the dark for 20 min. Following incubation, the cells were diluted with 300 µL Binding Buffer (total volume 400 µL) and analyzed using flow cytometry. The overall cell apoptosis was assessed by combining the analysis of early and late apoptotic cells.

### 5.23 Flow Cytometry Analysis of Cell Cycle Distribution

*G. morbillorum* or *E. coli* MG1655 treated cells were subjected to trypsinization, collected, and washed with PBS. Subsequently, the cells were centrifuged to remove the supernatant. The cells were then resuspended in pre‐cooled 70% ethanol, gently mixed, and fixed at −20°C for 2 h. After fixation, the cells were washed twice with PBS and centrifuged to remove the supernatant. The cells were resuspended in PBS and stained with an appropriate amount of PI staining solution (containing RNase A), achieving a cell concentration of approximately 1 × 10^6^/mL. The stained cell samples were incubated in the dark at room temperature for 30 min. Following incubation, the stained cell samples were transferred to the sample tubes of the flow cytometer for detection. The cell cycle distribution was analyzed using the flow cytometer to determine the percentages of cells in the G0/G1 phase, S phase, and G2/M phase.

### 5.24 Confocal Microscopy

Cells were seeded in glass‐bottom culture dishes. After adherence, cells were co‐incubated with *G. morbillorum* or *E. coli* under anaerobic conditions for 0.5 h. Subsequently, the cells were washed twice with PBS and then fixed with 4% paraformaldehyde at room temperature for 30 min. After fixation, the cells were washed three times with PBS, permeabilized with 0.1% Triton X‐100 in PBS for 10 min, and washed again three times with PBS. For staining, CellMask Actin Tracking Stains (Invitrogen, Cat. No. A57243) were employed. The CellMask Actin Tracking Stains stock solution was diluted to 1× in PBS to prepare a 1×staining solution. DAPI was added to the staining solution at a 1×concentration. Sufficient 1× staining solution was added to cover the cells and incubated for 15 min. The sample was then washed three times with PBS. Following the adjustment of parameters on the confocal microscope, images were captured.

### 5.25 Transmission Electron Microscopy

Monolayer cells were co‐incubated with bacteria under anaerobic conditions for 0.5 h. Subsequently, the cells were washed once with PBS. 2.5% Glutaraldehyde was then applied to cover the cells, which were incubated at 4°C for 30 min. Afterward, the cells were washed three times with PBS. Osmium tetroxide was applied to cover the monolayer cells, which were incubated at room temperature for 10 min. The cells were then washed twice with PBS, each time for 2 min. The cells were next subjected to a graded ethanol series, being immersed twice in each of the following concentrations: 30%, 50%, 70%, 75%, 80%, 85%, 90%, and 95%, with each immersion lasting 3 min, followed by two changes of 100% ethanol (3 min each). Embedding medium was added, and the cells were incubated at 37°C for 24 h. The embedding medium was then replaced, and the sample was placed at 60°C until the medium hardened. The sample was then sectioned and placed under a transmission electron microscope for observation at a magnification of 12 000 times.

### 5.26 RNA Sequencing

Cells were co‐incubated with bacteria anaerobically for 1.5 h daily over 3 days. After removing the medium, cells were lysed with TRIzol, mixed with an equal volume of chloroform, and incubated at room temperature for 10 min. The mixture was centrifuged at 12 000×g for 10 min, the aqueous phase transferred to a new tube, and an equal volume of isopropanol added. After mixing and a 5‐min room‐temperature incubation, the solution was centrifuged at 12 000×g for 10 min. The RNA pellet was washed with 70% ethanol and dissolved in RNase‐free water. RNA integrity and concentration were assessed using agarose gel electrophoresis, a bioanalyzer, and a NanoDrop spectrophotometer. RNA was fragmented and size‐selected to obtain sequencing‐compatible fragments, which were reverse‐transcribed into cDNA for amplification and sequencing. Sequencing adapters were ligated to the cDNA fragments for platform recognition, and the library was loaded onto the sequencer for high‐throughput sequencing. Sequencing data quality was checked using FastQC (RRID:SCR_014583) to identify and address issues, such as low‐quality bases and overrepresented adapters. Short reads were aligned to the reference genome using a sequence alignment tool to determine genomic locations, and gene expression levels were estimated by counting reads mapped to each gene.

### 5.27 Western Blot

Cells were co‐cultured with bacteria anaerobically for 1.5 h and maintained for 3 days. Cells were then lysed with RIPA buffer (containing protease/phosphatase inhibitors), and the supernatant was collected after centrifugation. Samples were mixed with Loading Buffer (containing SDS and β‐mercaptoethanol) and denatured at 100°C for 5 min. Protein samples (10–20 µL) were loaded onto an SDS‐PAGE gel selected based on the target protein's molecular weight and electrophoresed at 150 V for 1 h until the bromophenol blue reached the gel bottom. The PVDF membrane was soaked in cold (4°C) transfer buffer for 30 s, rinsed with deionized water, and transferred without bubbles. The membrane was blocked with 5% skim milk in TBST for 2 h at room temperature or overnight at 4°C, then washed three times with TBST (7 min each). Primary antibodies diluted in 5% skim milk TBST were incubated overnight at 4°C, followed by three 7‐min TBST washes. HRP‐conjugated secondary antibodies were incubated for 2 h at room temperature, then detected via chemiluminescence after a final TBST wash. Protein expression was quantified using imaging software. Uncropped blots are available in Figure S17.

### 5.28 Co‐Immunoprecipitation (Co‐IP)

Cells were transfected with a plasmid encoding TMEM140 containing a C‐terminal 6×His tag. Successful overexpression was confirmed by Western Blot. Transfected cells were lysed in RIPA buffer and incubated with *G. morbillorum* whole‐cell lysate at room temperature for 2 h with gentle agitation. The lysate was processed using a nickel column to capture His‐tagged TMEM140. After denaturation in WB loading buffer (5 min, 100°C), proteins were resolved by SDS‐PAGE and visualized by silver staining using the BeyoSilver Silver Stain Kit (Beyotime, Cat. No. P0925M). Protein bands that showed differential binding were excised for mass spectrometric identification of interacting partners.

### 5.29 Assessment of Bacterial Invasion Capacity Into Cells

*G. morbillorum* and *E. coli* MG1655 were labeled with CellTrace CFSE (Thermo Fisher, Cat No. C34554). The dye was prepared as a 10 mM stock solution in DMSO and stored at −20°C protected from light. For bacterial labeling, strains were resuspended in 1 mL of 1×CFDA‐SE Buffer (containing 5 µM CFSE working concentration) in a 15 mL centrifuge tube to a density of 1–5×10^6^ CFU/mL. After incubation in the dark under anaerobic conditions for 30 min, the mixture was centrifuged at 3500×g and washed twice with PBS to remove unbound dye. The bacterial pellet was then resuspended in bacterial culture medium and incubated for an additional 2 h in the dark under anaerobic conditions. The labeled bacteria were subsequently co‐incubated with cells in the dark under anaerobic conditions for 1.5 h. Following cell digestion, the cellular pellet was collected via centrifugation and resuspended in PBS. The average fluorescence intensity of FITC in the cells was measured using a flow cytometer to evaluate the bacterial invasion capacity.

### 5.30 IHC

Paraffin sections were dewaxed in fresh xylene (10 min × 3 times), then rehydrated through graded ethanol (100% × 2 times, 95%, 90%, 80%, 5 min each), and washed in sterile water (1 min × 3 times). Antigen retrieval was performed using 1× citrate buffer (pH 6.0) in a microwave: high power until boiling, then medium‐high power for 10 min, followed by medium‐low power for 3 min. After cooling to room temperature, sections were washed with PBS (2 min × 3 times). Tissue was circled with immunohistochemistry pen (≈2 mm from the tissue), and endogenous peroxidase was blocked with 50 µL H_2_O_2_ at 37°C for 15 min in a humidified chamber. Sections were blocked with 5% skim milk in TBST for 2 h at room temperature or overnight at 4°C, incubated with primary antibody (diluted in 5% skim milk TBST) overnight at 4°C, and washed with TBST (7 min × 3 times). HRP‐conjugated secondary antibody was applied for 2 h at room temperature. Sections were developed with DAB chromogenic substrate, imaged, and protein expression was quantified using imaging software.

### 5.31 SPR Analysis

A CM5 sensor chip was installed on a Biacore T200 instrument according to standard operating procedures. Pre‐concentration assays were performed to determine optimal immobilization conditions: TMEM140 ligand was diluted in 10 mM sodium acetate (NaAc) buffers at varying pH (4.0, 4.5, 5.0, and 5.5) and injected over the chip surface. Maximum pre‐concentration was observed at pH 4.0. Therefore, 10 mM NaAc (pH 4.0) was selected as the immobilization buffer. Target immobilization was calculated as 200 RU. The chip surface was activated with EDC/NHS (1:1) solution, followed by injection of TMEM140 (20 µg/mL) diluted in 10 mM NaAc (pH 4.0) using targeted immobilization mode. The surface was subsequently deactivated with ethanolamine, achieving a final immobilization level of 429.9 RU. For regeneration scouting, the analyte LPBDCP was diluted to 1 µM in 0.05% Tween‐PBS (pH 7.4) and tested against various regeneration buffers (10 mM glycine‐HCl at pH 1.5, 2.0, 2.5, and 3.0). Based on optimal binding capacity and baseline stability, 10 mM glycine‐HCl (pH 1.5) was selected as the regeneration solution. Kinetic analysis was performed using a two‐fold serial dilution series of LPBDCP‐mutant in 0.05% Tween‐PBS (pH 7.4) at concentrations of 2000, 1000, 500, 250, 125, 62.5, 31.25, 15.625, 7.8125, 3.90 625, and 0 nM. Samples were injected at a flow rate of 30 µL/min with an association phase of 180 s and a dissociation phase of 300 s. Data were analyzed using Biacore T200 Evaluation Software (version 3.2.1) with a kinetic analysis model.

### 5.32 Detection of GTP‐Ras

After co‐culture with *G. morbillorum* for 2 h, cells were washed with ice‐cold TBS and lysed in Mg^2^^+^‐containing lysis buffer (25 mM HEPES pH 7.5, 150 mM NaCl, 1% NP‐40, 0.25% sodium deoxycholate, 10 mM MgCl_2_, 10% glycerol, supplemented with protease and phosphatase inhibitors) on ice for 20 min. Lysates were centrifuged at 14 000 × g for 5 min at 4°C, and supernatants were subjected to protein quantification. Equal amounts of protein (200 µg) were incubated with pre‐washed Raf1‐RBD agarose (Beyotime Active Ras Pull‐Down Assay Kit, P2433S) at 4°C for 1–3 h with rotation. After washing three times with wash buffer, bound proteins were eluted by boiling in 1× SDS sample loading buffer for 5 min. Pull‐down products and Input (normalized to GAPDH) were resolved by SDS‐PAGE, transferred to PVDF membranes, and probed with anti‐K‐Ras antibody (Abcam ab180772, 1:1000). Immunoreactive bands were visualized by ECL chemiluminescence.

### 5.33 Gentamicin Protection Assay

CRC cell lines were seeded in 10 cm cell culture dishes and cultured at 37°C with 5% CO_2_ until reaching 80%–90% confluency. *G. morbillorum* was grown to mid‐log phase, and bacterial density was determined by measuring OD600. The bacteria were collected by centrifugation, washed once with pre‐warmed PBS, and resuspended in antibiotic‐free DMEM. Cells were infected with *G. morbillorum* at a multiplicity of infection (MOI) of 10 under anaerobic conditions for 0.5 h. Following infection, the culture medium was removed, cells were washed with PBS, and DMEM containing 200 µg/mL gentamicin was added to kill extracellular bacteria. After 2.0 h of incubation at 37°C with 5% CO_2_, the gentamicin‐containing medium was removed, and cells were washed three times with PBS. Subsequently, cells were lysed with 0.1% Triton X‐100 for 10 min at room temperature with gentle agitation to ensure complete lysis. The lysates were serially diluted 10‐fold with PBS, and 200 µL of each dilution was plated onto Columbia blood agar plates, followed by anaerobic incubation at 37°C for 48 h. The bacterial invasion efficiency was calculated as follows

(1) $$\begin{array}{ccc} \mathrm{Invasion}\;\mathrm{efficiency}\;\left(\%\right) & = & \mathrm{CFU}\left(\mathrm{intracellular}\right)/\mathrm{CFU}\left(\mathrm{inoculum}\right)\\ & & \times \;100 \end{array}$$

### 5.34 Biochemical Analysis of NDPD‐Mediated p53 Deacetylation

In vitro deacetylation assays were performed using recombinant His‐tagged NDPD protein (expressed in *E. coli* BL21(DE3) and purified via nickel‐affinity chromatography) and acetylation‐enriched Flag‐tagged p53 (overexpressed in NCM460 cells, treated with trichostatin A and nicotinamide, and isolated using anti‐Flag immunomagnetic beads). Reactions were conducted in buffer containing 50 mM Tris‐HCl (pH 8.0), 150 mM NaCl, 1 mM MgCl_2_, and 0.5 mM DTT at 37°C for 2 h. Four experimental groups were established: buffer control, *E. coli* lysate supplemented with NAD^+^, NDPD alone (without NAD^+^), and NDPD with NAD^+^. Following reaction termination, samples were resolved by SDS‐PAGE and analyzed by immunoblotting using site‐specific antibodies against acetylated p53.

### 5.35 Determination of Animal Sample Size

A power analysis using G*Power 3.1 (effect size *d* = 0.8–1.2, *α* = 0.05, power ≥ 0.8), which indicated a minimum requirement of 5–6 mice per group. Considering an anticipated 20% attrition rate due to humane endpoints in the AOM/DSS model, we finalized the sample size at 6–8 mice per group. This aligns with the Reduction principle of the 3Rs while ensuring adequate statistical power. The actual experimental results revealed significant between‐group differences (*p* < 0.01), confirming that this sample size was sufficient to support our conclusions.

### 5.36 Statistical Analysis

Statistical analyses for metagenomic data (including batch effect correction, differential abundance testing, and diagnostic model construction) are detailed in Sections 6.8 and 6.9.

For Western blot densitometry, raw band intensities were normalized to loading controls (β‐actin, GAPDH, or Na/K ATPase β1) and expressed as relative ratios to correct for protein loading variations. No outlier exclusion or arbitrary mathematical transformation was applied to these normalized values. For transcriptomic analysis (Figure 5A), raw count data were normalized using the median of ratios method as implemented in DESeq2 to account for library size differences. Differential expression analysis was performed using DESeq2. For visualization in volcano plots, fold changes were log_2_‐transformed (log_2_FC) and false discovery rates (FDR) were negative log10‐transformed (‐log10FDR). Genes with |log_2_FC| > 1 and FDR < 0.05 were considered statistically significant.

All quantitative data are presented as mean ± SD. Comparisons between two groups used unpaired Student's *t*‐tests. Multi‐group comparisons employed one‐way ANOVA with Tukey's post‐hoc test. The significance level (α) was set at 0.05 (two‐tailed). Exact sample sizes (n) are specified in the corresponding figure legends (n denotes biologically independent replicates or animals). Analyses were conducted using R 4.2.0/4.3.0 (randomForest, pROC, vegan, DESeq2 packages), GraphPad Prism 9.0, and SPSS 26.0.

## Author Contributions

Z.W. and J.Z. contributed equally to this work. L.L., Z.W., and J.Z. were responsible for the overall study design, data interpretation, and drafting of the manuscript. Z.W., S.Y., and Y.S. contributed to the experimental design and the laboratory work. J.Z., H.L., J.N., and S.Z. collected and managed the data, software operation, conducted the initial data analysis and advanced statistical analysis, and interpreted the results. L.L. critically revised the manuscript for important intellectual content. All authors read and approved the final manuscript.

## Funding

This work was supported by the National Natural Science Foundation of China (Nos. 82473713 and 82173602).

## Ethical Statement

The collection of fecal samples from patients was approved for research ethics by the Ethics Committee of Tongji Medical College, Huazhong University of Science and Technology (approval code: 2021 IEC‐A189), and the animal experiments were approved by Institutional Animal Care and Use Committee, Huazhong University of Science and Technology ([2024] IACUC Number: 4638).

## Conflicts of Interest

The authors declare no conflicts of interest.

## Supporting information




**Supporting File 1**: advs75090‐sup‐0001‐SuppMat.docx.

**Supporting File 2**: advs75090‐sup‐0002‐TableS1‐S5.xlsx.

## Data Availability

Data generated or analyzed during this study are included data generated or analyzed during this study are included in this published article and its supplementary information file. The raw metagenomic sequencing data has been uploaded to the SRA database (BioProject: PRJNA1195392 at https://dataview.ncbi.nlm.nih.gov/object/PRJNA1195392?reviewer=b2ffo0sv7o786ecffsljiantsk).
