# Supplementary material for: Gemella morbillorum Promotes Colorectal Carcinogenesis: LPBDCP‐Mediated Invasion Activates Ras Signaling and Destabilizes p53
Source: Adv Sci (Weinh). 2026 Apr 7;13(34):e17245. doi: 10.1002/advs.202517245 (PMC13285141; doi:10.1002/advs.202517245)
Supplement: Supplementary file 1 — Supporting File 1: advs75090‐sup‐0001‐SuppMat.docx. [file ADVS-13-e17245-s001.docx]

**Supplementary Figures**


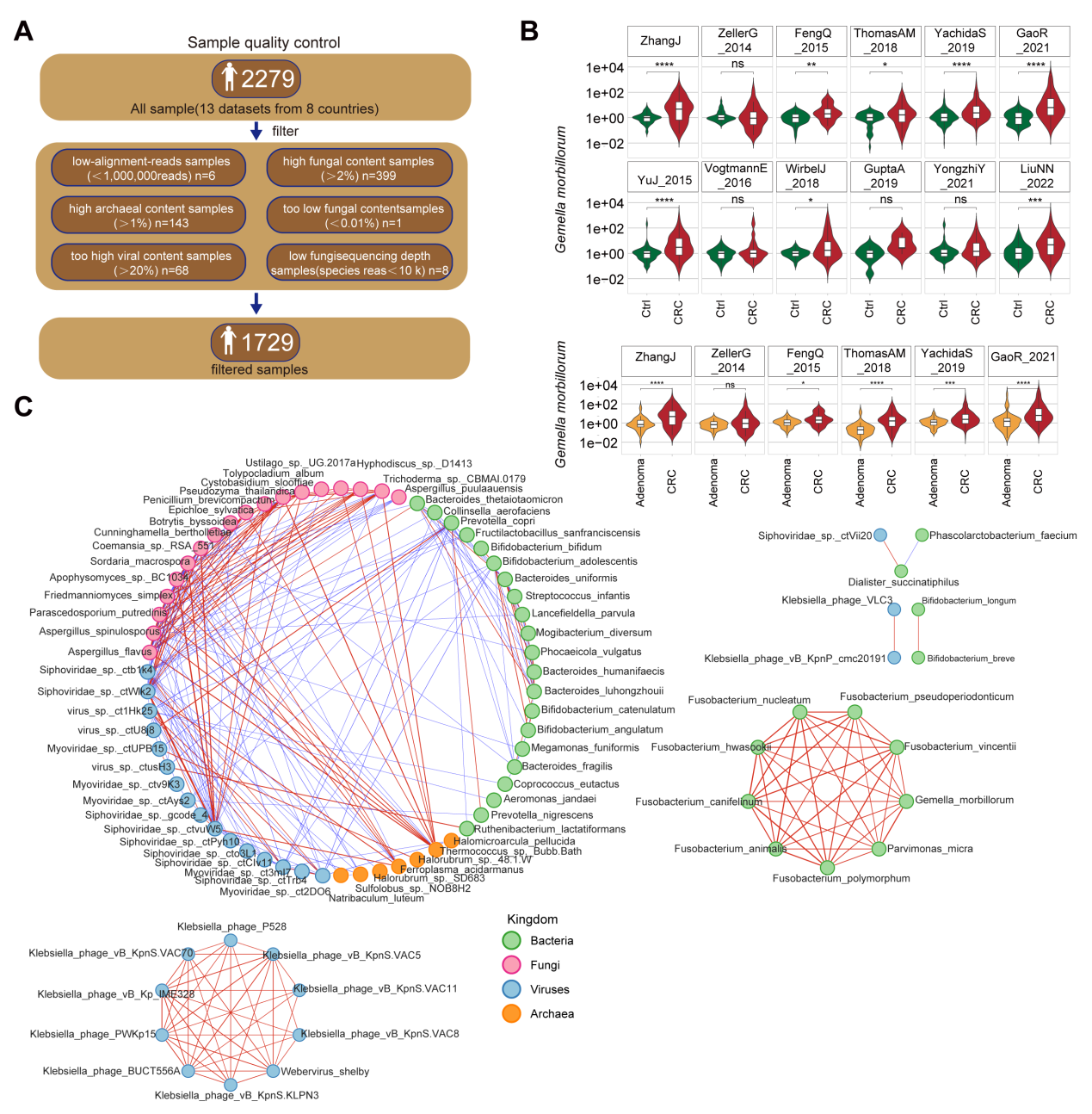


**Supplementary Figure 1.** *G. morbillorum* exhibits elevated abundance in the stool of colorectal cancer patients.

1. Data filtering process flowchart from 13 cohorts.
2. Variations in *G. morbillorum* levels in individual studies across CRC, CRA, and control groups. **p* < 0.05, ***p* < 0.01, ****p* < 0.001, *****p* < 0.0001.
3. Co-occurrence analysis of multi-kingdom microorganisms in CRC fecal samples.


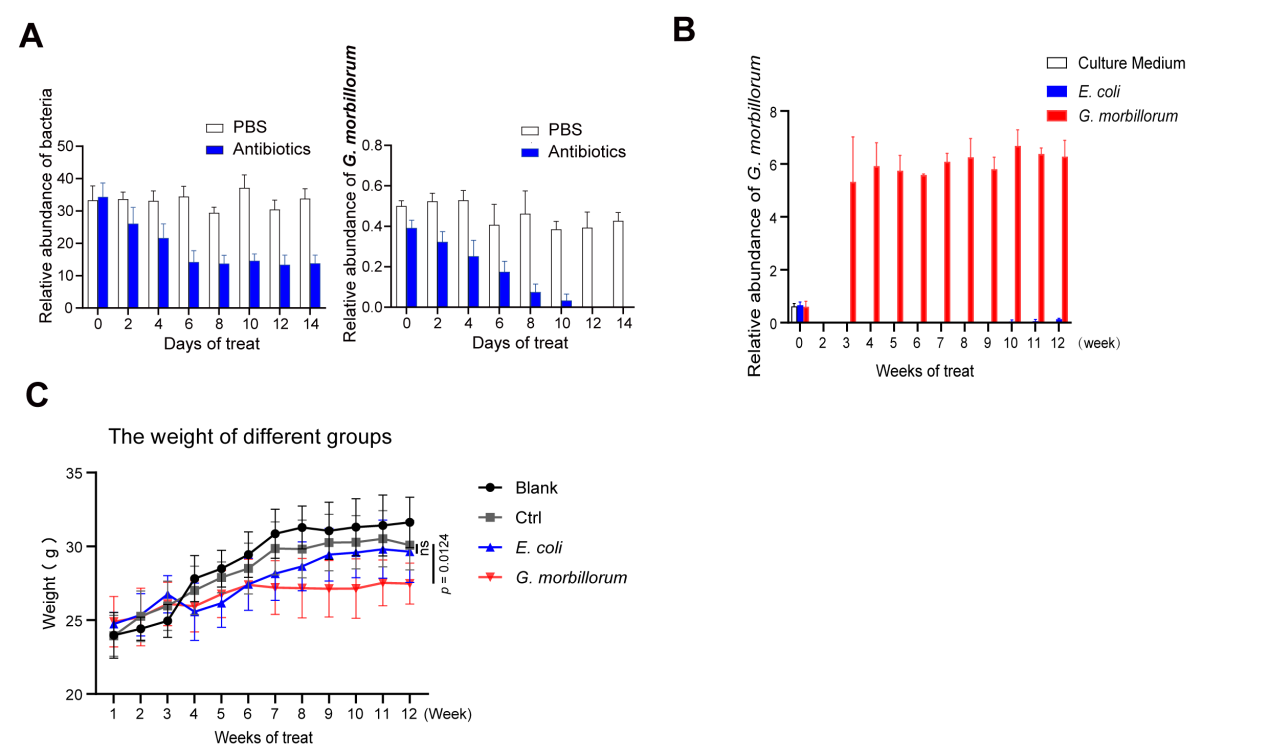


**Supplementary Figure 2.** *G. morbillorum* promotes murine colorectal carcinogenesis.

(A) The left panel illustrates the temporal changes in fecal total bacterial abundance in different groups of mice following antibiotic cocktail treatment, while the right panel depicts the changes in *G. morbillorum* abundance over time. Bacterial abundance was measured by qPCR after DNA extraction from fecal samples.

(B) Mice were orally administered *G. morbillorum* or *E. coli* MG1655, and the abundance of *G. morbillorum* in the gut was monitored over time via qPCR after depleting gut microbiota.

(C) Body weight changes of mice in each treatment group over time. Ctrl: control

Data are presented as mean ± SD. Multi-group comparisons were performed using one-way ANOVA followed by Tukey's post-hoc test. The significance level (α) was set at 0.05 (two-tailed).


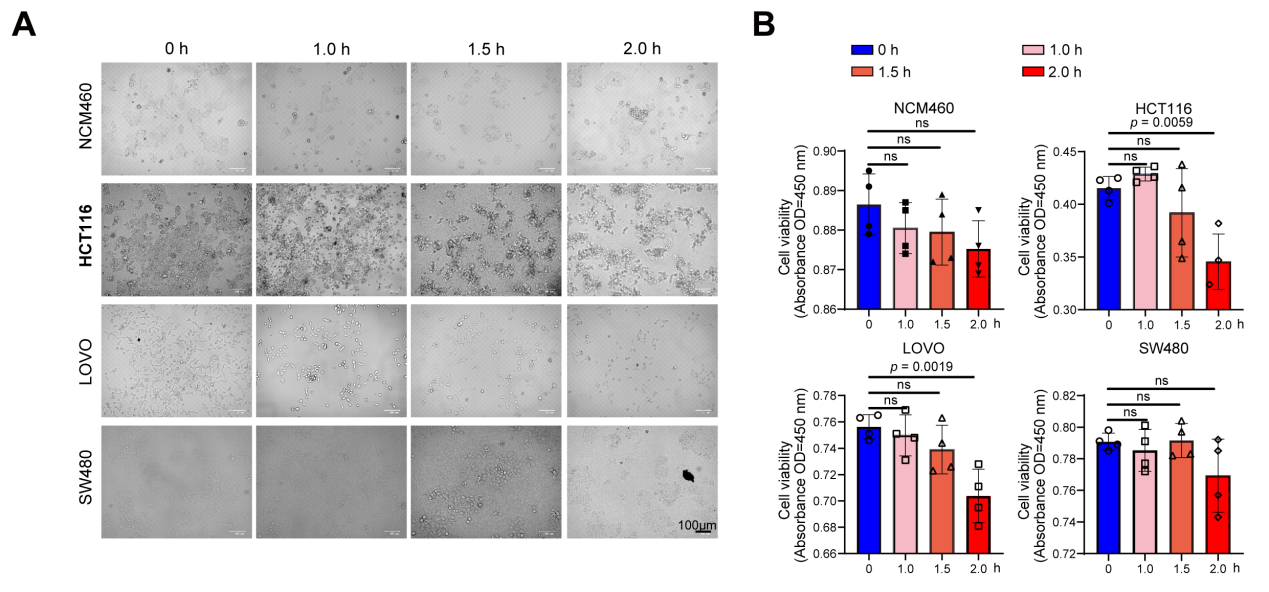


**Supplementary Figure 3.** Hypoxia tolerance assay of NCM460 and CRC lines.

(A) Morphological changes in various cell lines with increasing hypoxia duration.

(B) CCK-8 assay measuring the changes in cell viability of various cell lines as hypoxia duration increases.

Data are presented as mean ± SD. Multi-group comparisons were performed using one-way ANOVA followed by Tukey's post-hoc test. The significance level (α) was set at 0.05 (two-tailed).


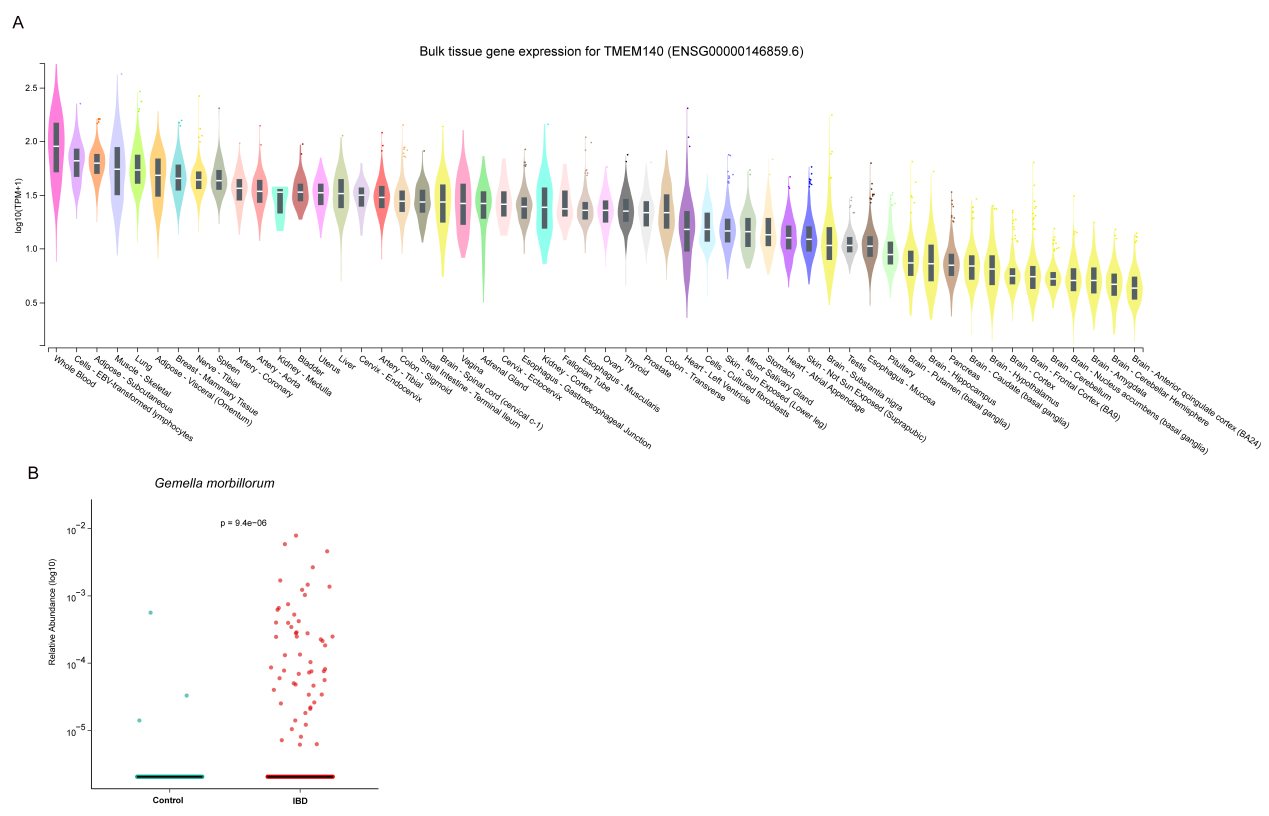


**Supplementary Figure 4.** Tissue distribution of TMEM140 and *G. morbillorum* enrichment in IBD patients.

(A) Tissue distribution of TMEM140 expression in normal tissues and organs, ranked from high to low. Data were derived from GTEx.

(B) Comparative analysis revealed that *G. morbillorum* abundance was significantly elevated in IBD patients (n=315) compared to healthy controls (n=8,690), with a 27.0-fold higher median abundance (*p* < 0.001). Data derived from the curatedMetagenomicData package.


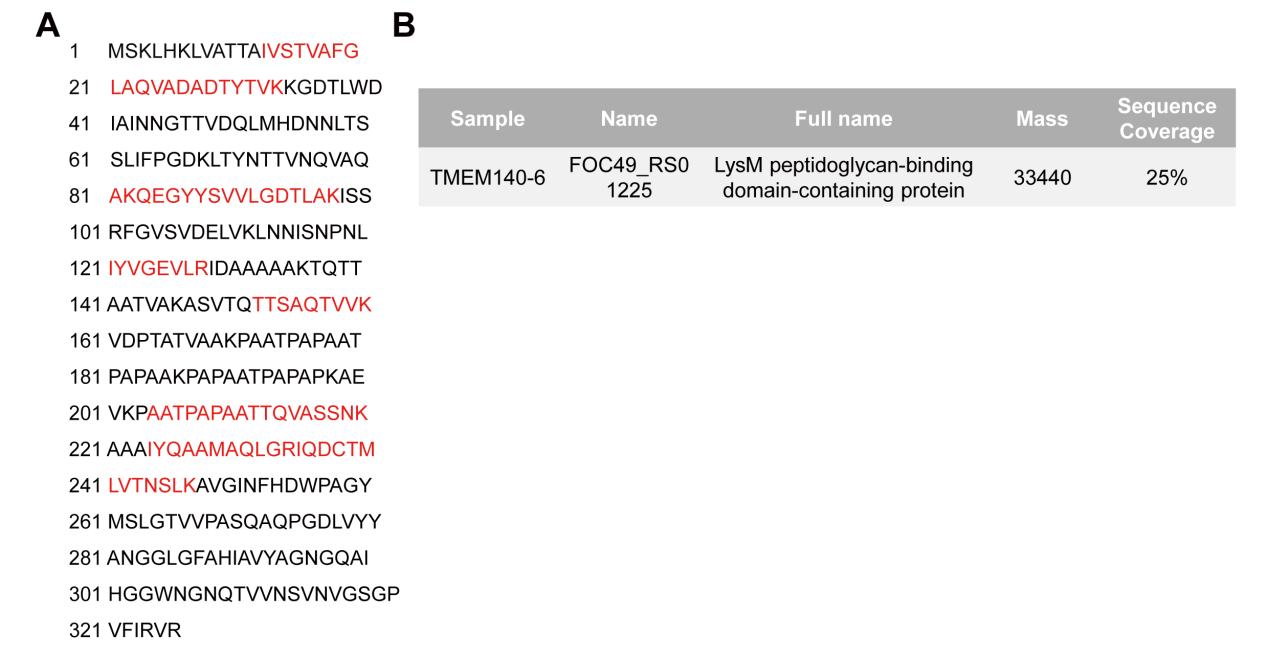


**Supplementary Figure 5**. Mass spectrometry identified LPBDCP as one of the proteins that bind to TMEM140.


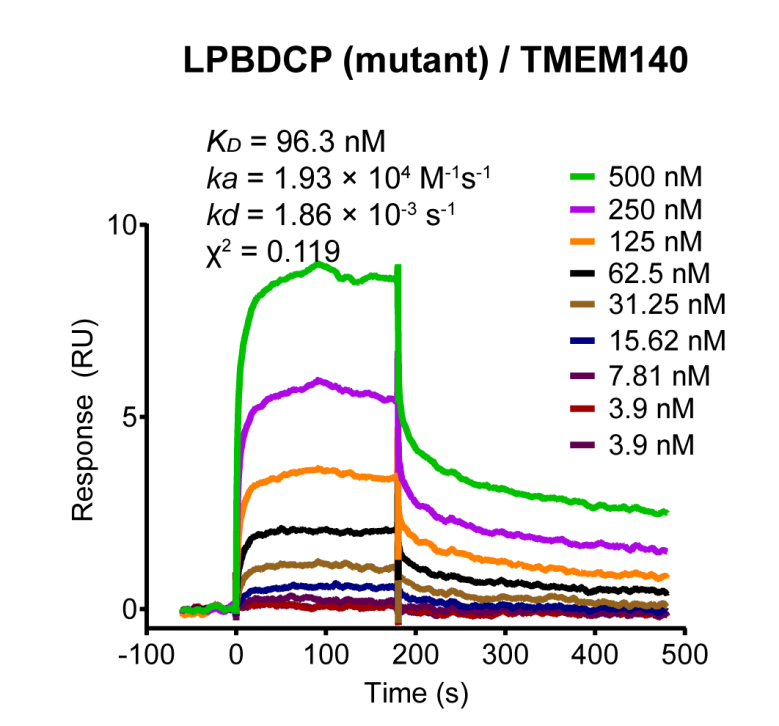


**Supplementary Figure 6**. Surface plasmon resonance analysis of LPBDCP (L127A mutant) binding to TMEM140.


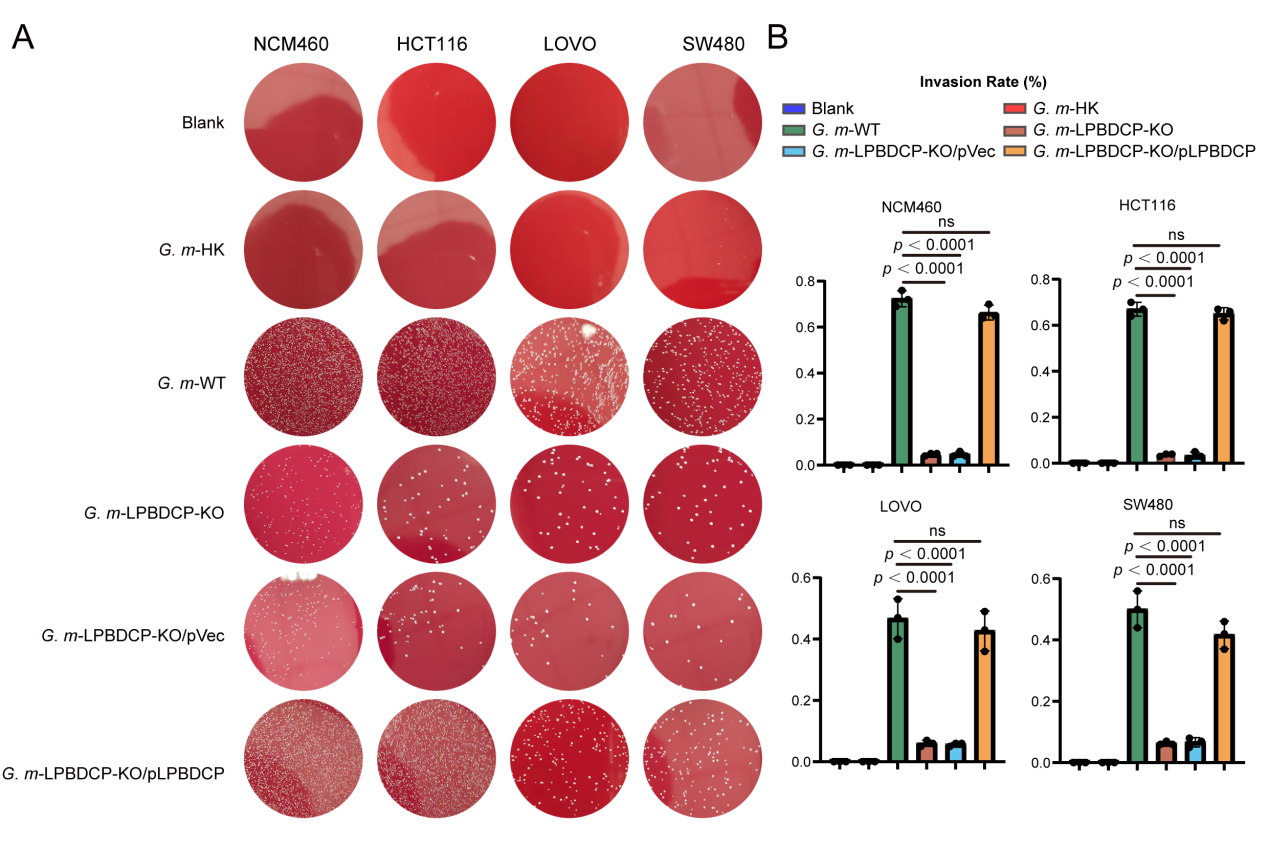


**Supplementary Figure 7**. Gentamicin protection assay for *G. morbillorum* invasion in CRC cells.

1. Representative plate showing intracellular *G. morbillorum* recovered following infection (MOI 10, 0.5 h), gentamicin killing (200 μg/mL), and cell lysis (0.1% Triton X-100) prior to plating and 48 h growth.
2. Statistical analysis of *G. morbillorum* invasion rates. Invasion rate (%) = (CFU intracellular / CFU total inoculum) × 100. *G. m*: *Gemella morbillorum*; KO:knockout; pVec: empty plasmid; pLPBDCP: LPBDCP overexpression plasmid

Data are presented as mean ± SD. Multi-group comparisons were performed using one-way ANOVA followed by Tukey's post-hoc test. The significance level (α) was set at 0.05 (two-tailed).


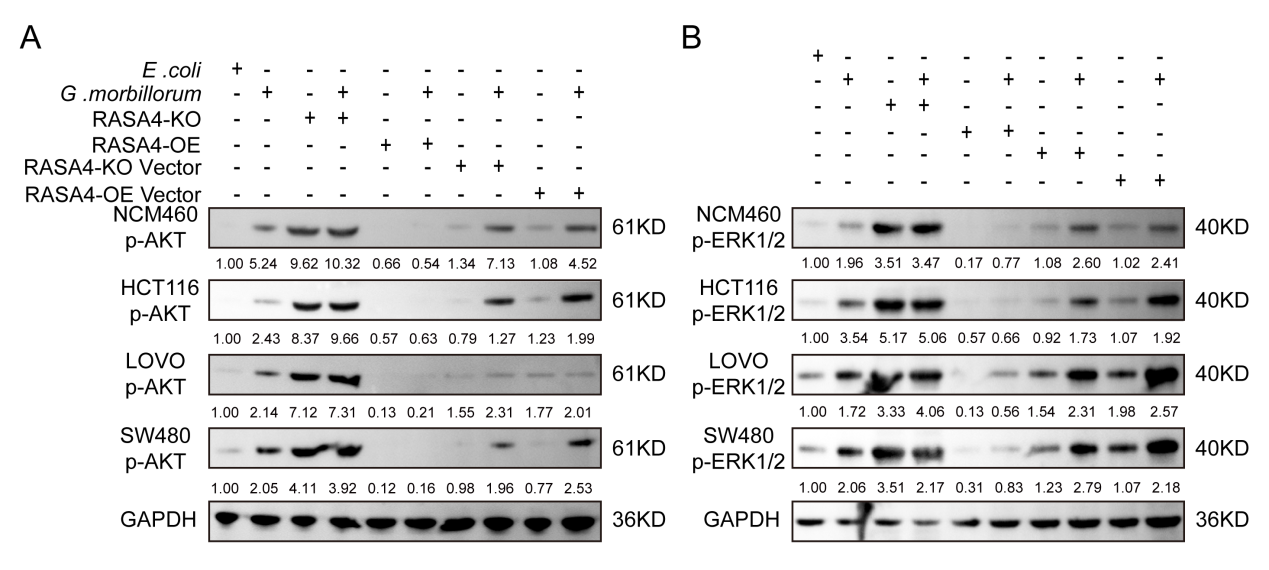


**Supplementary Figure 8**. Western blot analysis of RASA4 knockout or overexpression on p

-AKT and p-ERK1/2.

(A) Western blot analysis of RASA4 knockout or overexpression effect on p-AKT.

(B) Analysis of p-ERK1/2 upon RASA4 knockout or overexpression.

KO: knockout; OE: overexpression.


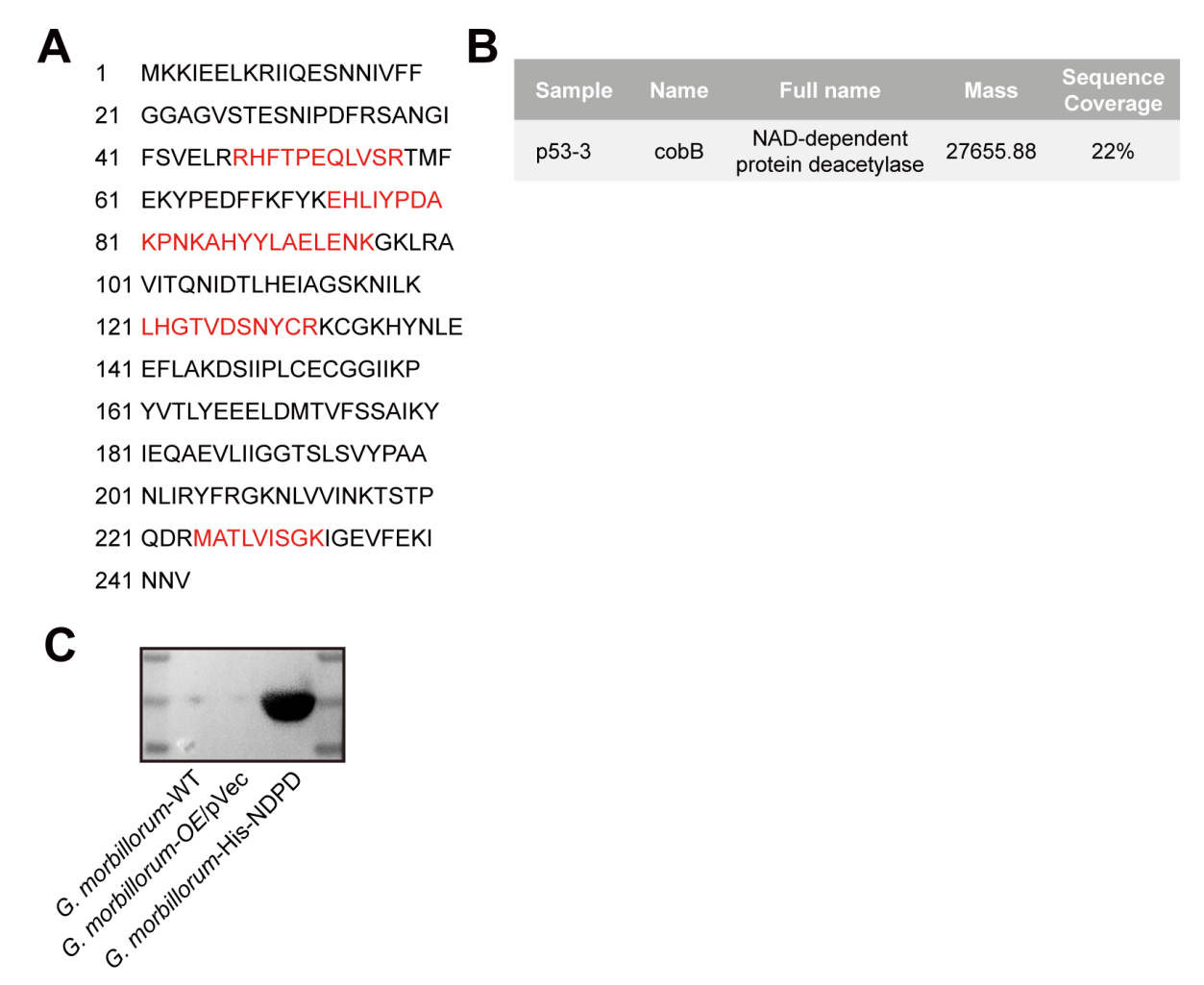


**Supplementary Figure 9**. NDPD interacts with p53.

(A) Mass spectrometry identified NDPD as one of the proteins that bind to p53.

(B) Mass spectrometry identification of NDPD.

(C) Western blot detection of His-NDPD secretion in the culture supernatant from *G. morbillorum* overexpressing His-NDPD.


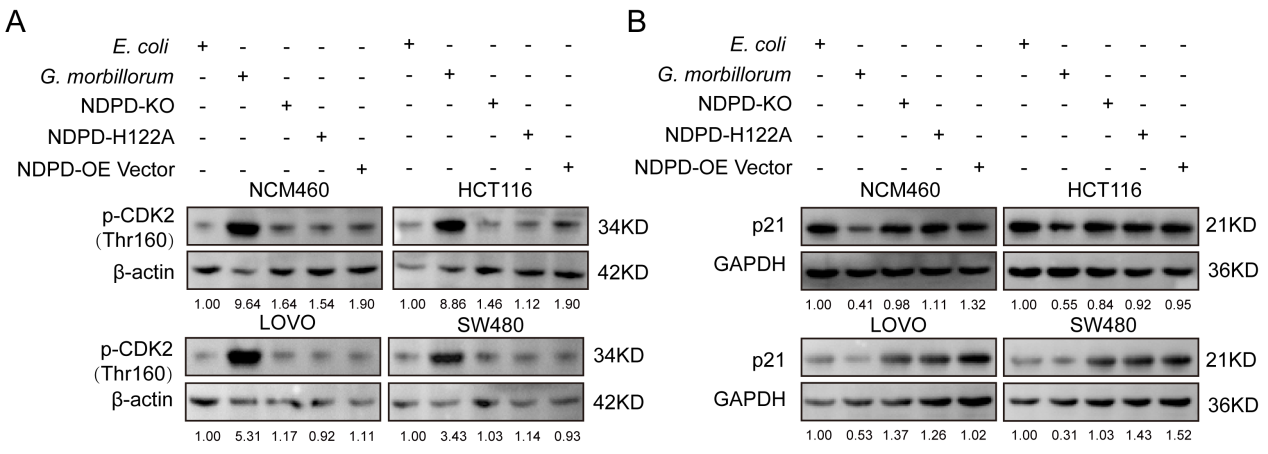


**Supplementary Figure 10**. Effects of NDPD-H122A and wild-type NDPD on downstream p53 pathways.

(A) Impact of NDPD-H122A and wild-type NDPD on CDK2 phosphorylation downstream of p53.

(B) Differential modulation of p53-downstream P21 by NDPD-H122A relative to wild-type NDPD.


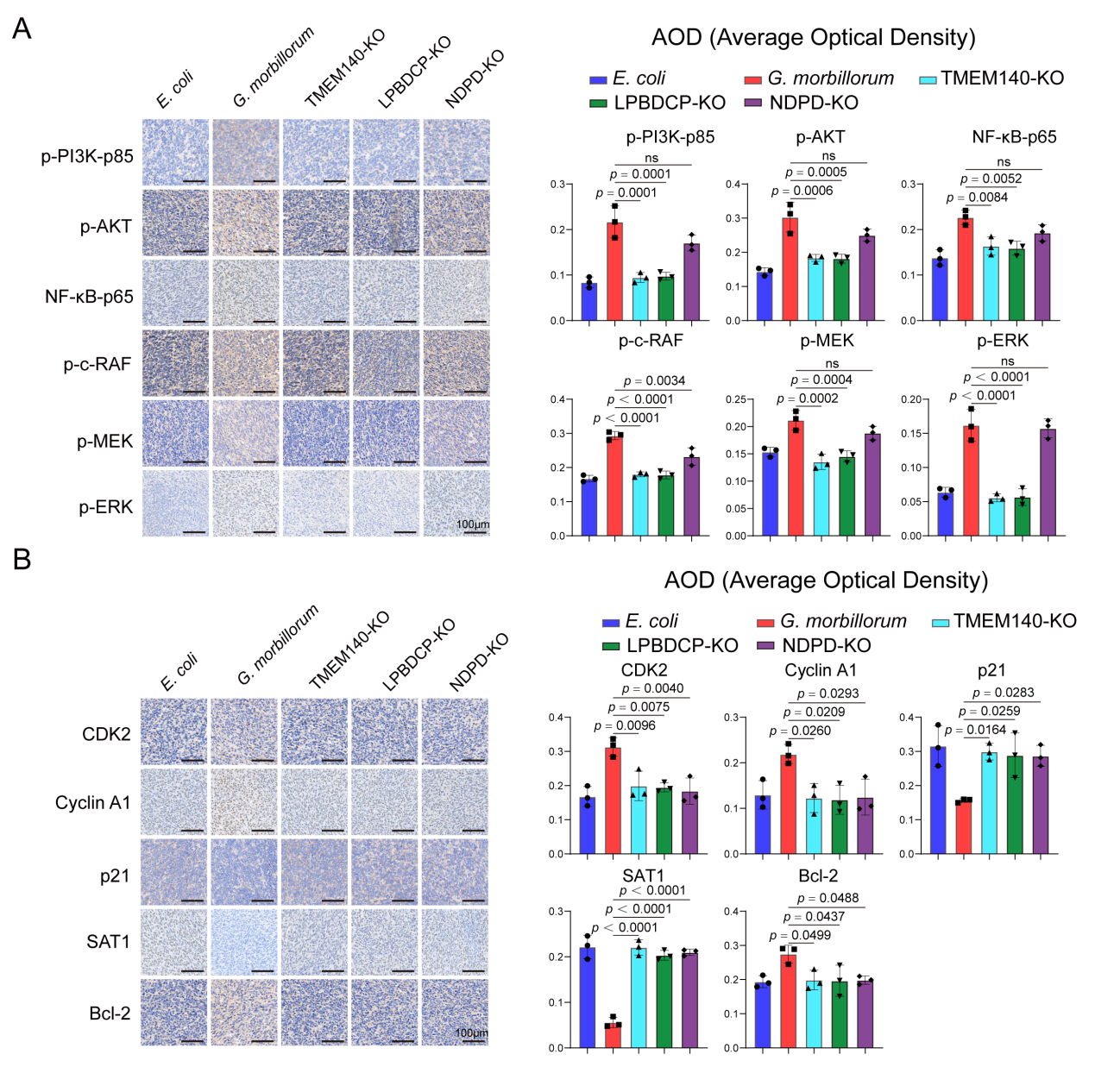


**Supplementary Figure 11**. Representative immunohistochemistry (IHC) images (left panels) and Average Optical Density (AOD) quantification (right panels) of key signaling molecules in tumor tissues.

(A) The left panel shows representative IHC images of changes in the levels of key molecules downstream of the Ras-RAF-MEK-ERK and Ras-PI3K-AKT-NF-κB pathways in tumor cells from different groups of mice, and the right panel presents the statistical analysis of differences in the Average Optical Density (AOD) of these molecules.

(B) The left panel displays representative IHC images of key molecules downstream of the p53 pathway, and the right panel shows the statistical analysis of differences.

Data are presented as mean ± SD. Multi-group comparisons were performed using one-way ANOVA followed by Tukey's post-hoc test. The significance level (α) was set at 0.05 (two-tailed).


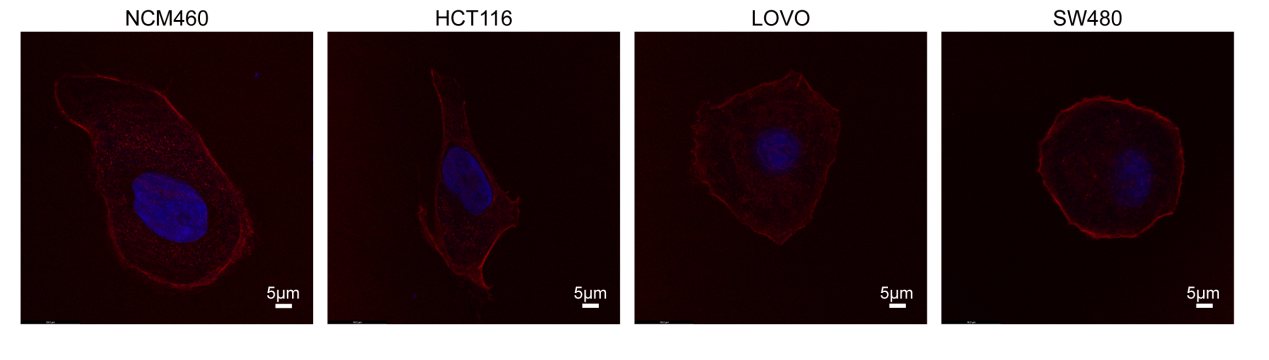


**Supplementary Figure 12**. Mycoplasma contamination was assessed by confocal microscopy following fixation and staining of cells with DAPI (blue, DNA stain) and a red plasma membrane dye (Invitrogen, Cat# A57243).


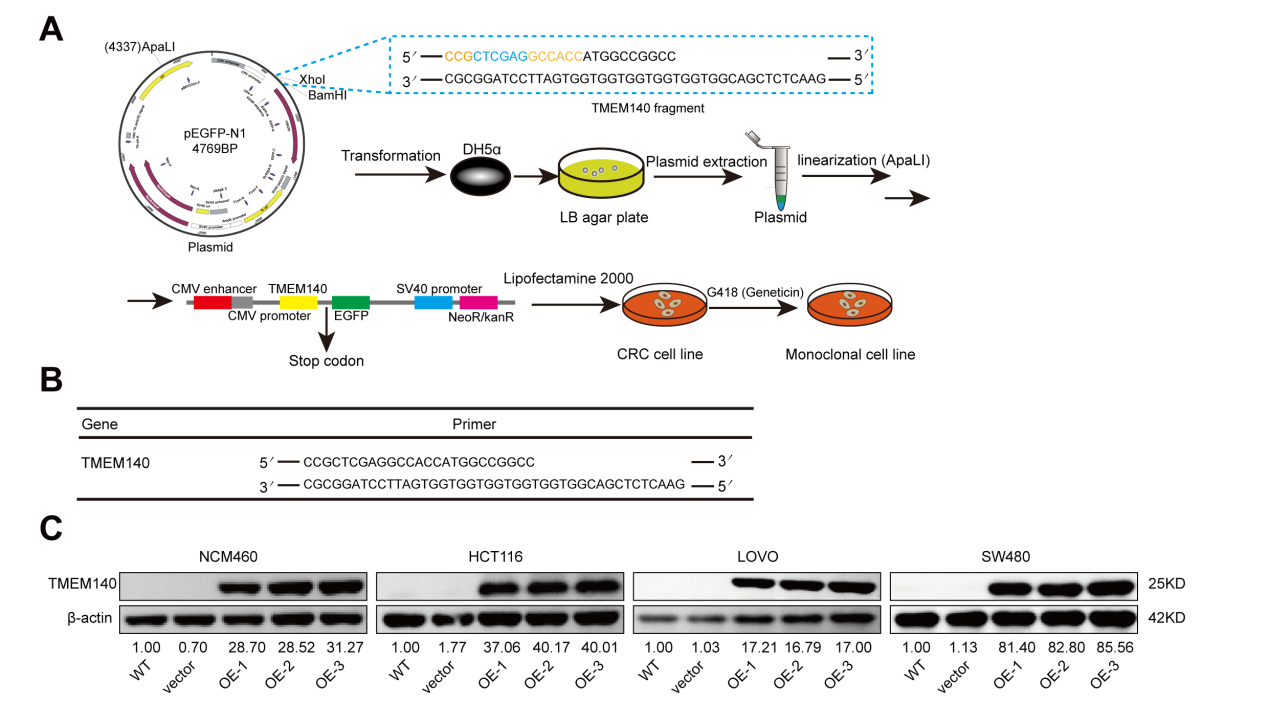


**Supplementary Figure 13.** Construction of a TMEM140 overexpression cell lines.

(A) Schematic diagram of the process for constructing an overexpression cell line of TMEM140. (B) Primers for the cDNA sequence of TMEM140.

(C) Comparison of TMEM140 expression levels between TMEM140-overexpressing cell lines and control cell lines. OE: overexpress

**
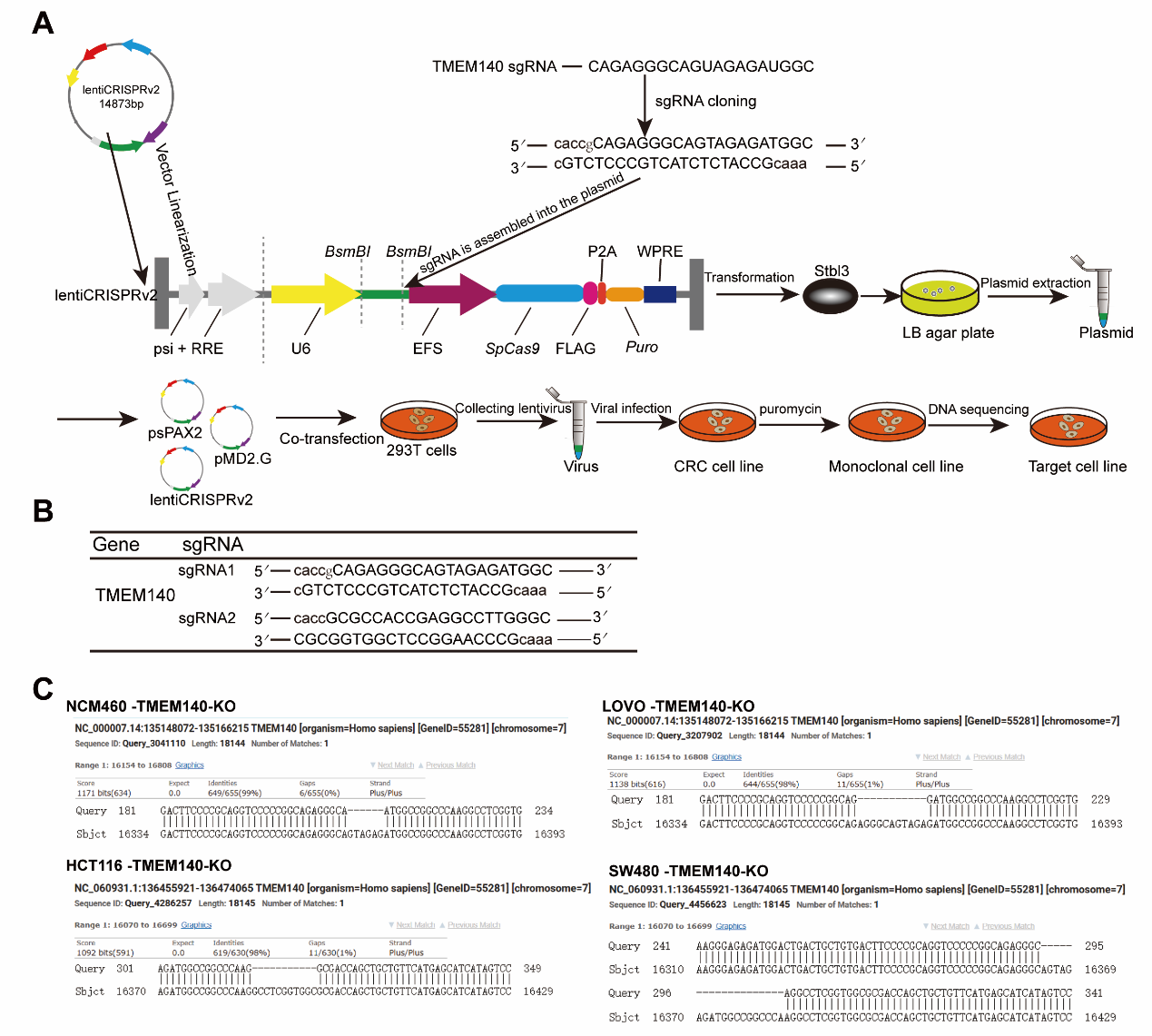
Supplementary Figure 14.** Construction of knockout cell lines for TMEM140.

(A) Schematic diagram of the process for constructing a monoclonal cell line with TMEM140 knockout.

(B) Primers for the double-stranded sgRNA of TMEM140.

(C) Align the DNA sequences of TMEM140 knockout cell lines to identify differences. KO: knockout


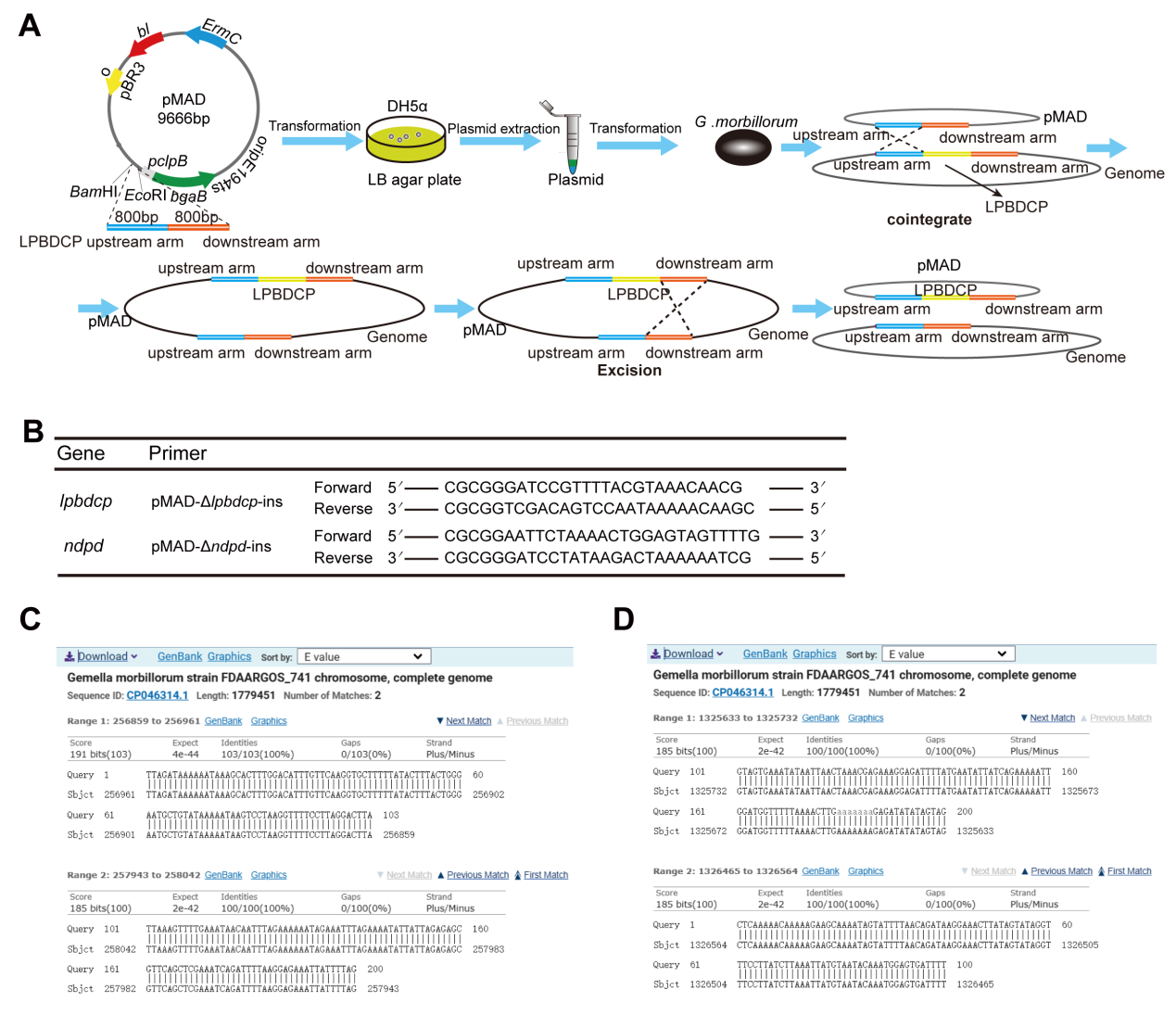


**Supplementary Figure 15**. Construction of gene knockout bacterial strains.

(A) Schematic diagram of the process for constructing a monoclonal bacteria line with the target gene knockout.

(B) The primer sequences of the upstream and downstream homologous arms of LysM peptidoglycan-binding domain-containing protein (LPBDCP) and NAD-dependent protein deacetylase (NDPD). pMAD: Homologous recombination plasmid;Δ*lpbdcp*: LPBDCP-knockout;

Δ*ndpd*: NDPD-knockout; UHA: The upstream homologous arm: DHA: The downstream homologous arm.

(C) Align the DNA sequences of LPBDCP in wild type bacteria and LPBDCP knockout bacteria to identify differences.

(D) Identify differences by aligning the DNA sequences of NDPD in wild-type bacteria and NDPD knockout bacteria.


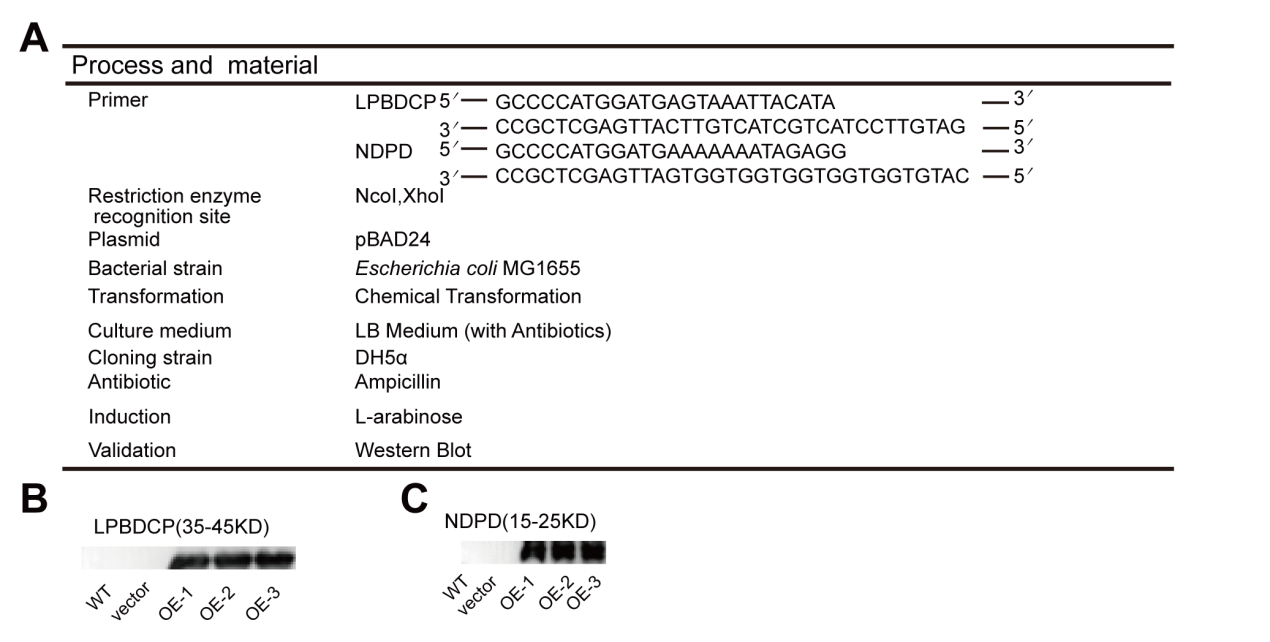


**Supplementary Figure 16**. Construction of bacterial strains for overexpression of the target proteins.

(A) Materials and key informations required for the construction of bacterial strains overexpressing the target proteins.

(B) Western blotting was performed to detect the protein expression levels in the LPBDCP-overexpressing bacterial strains.

(C) The difference in NDPD protein expression levels between NDPD-overexpressing bacterial strains and control strain.

OE: overexpress

**Supplementary Figure 17**. **Uncropped Western blot images.**


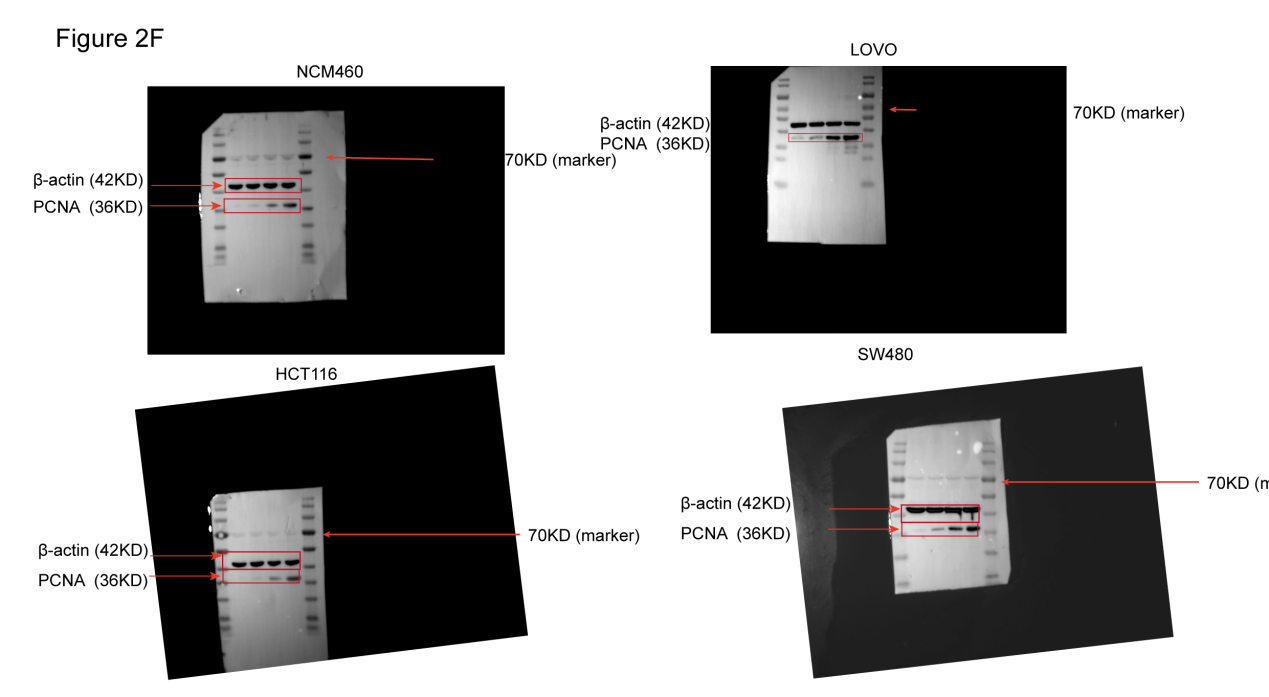


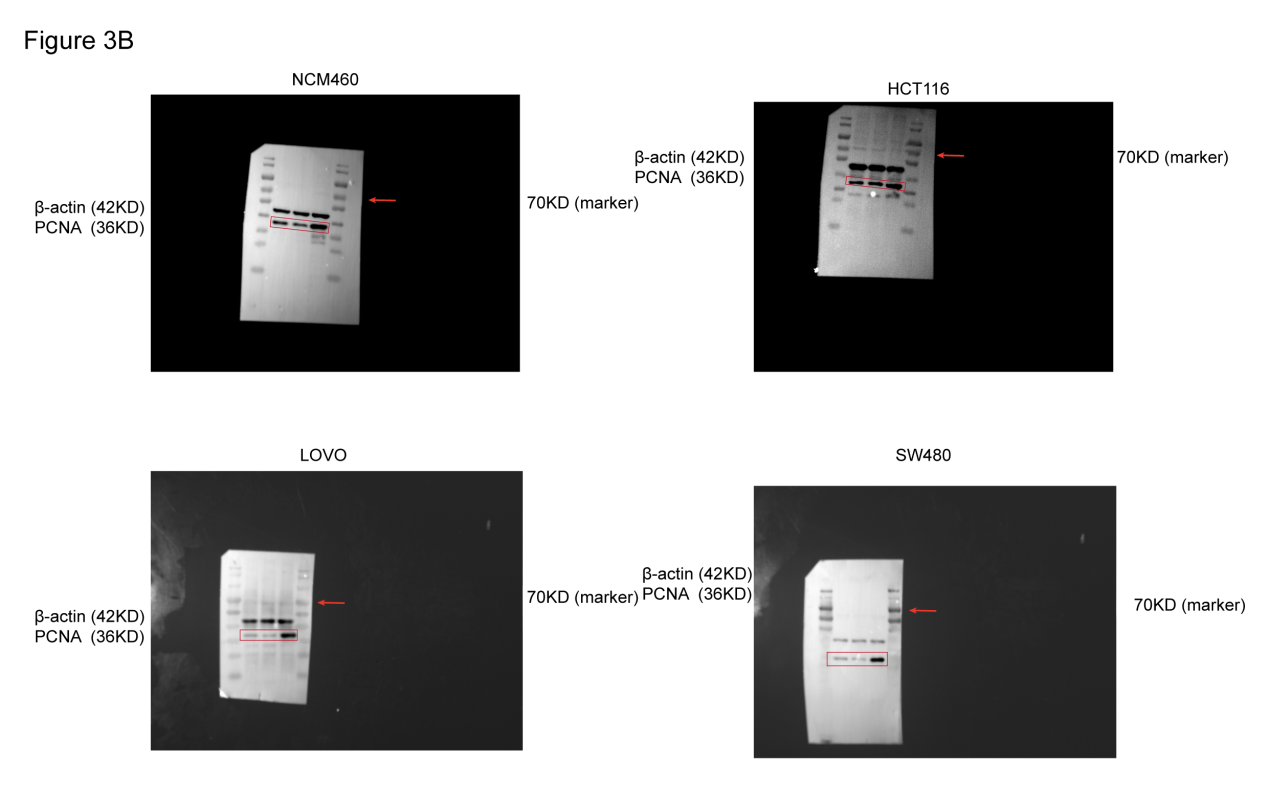

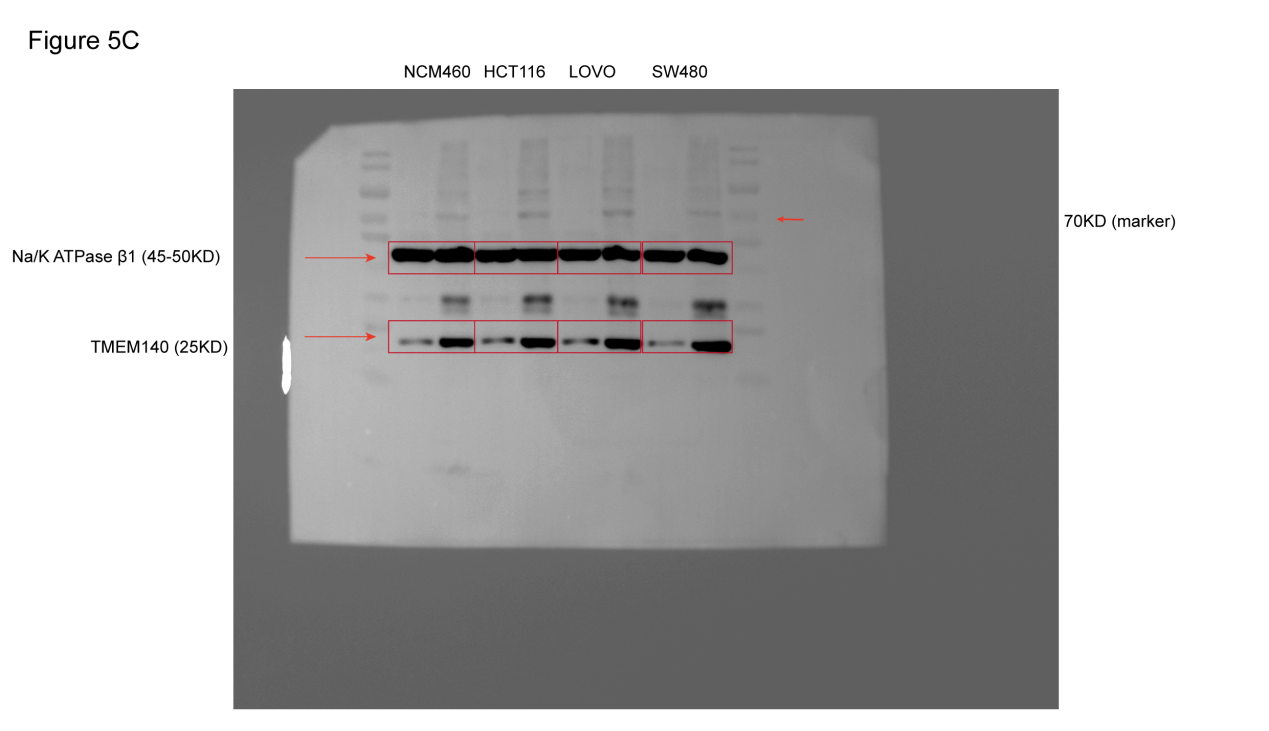

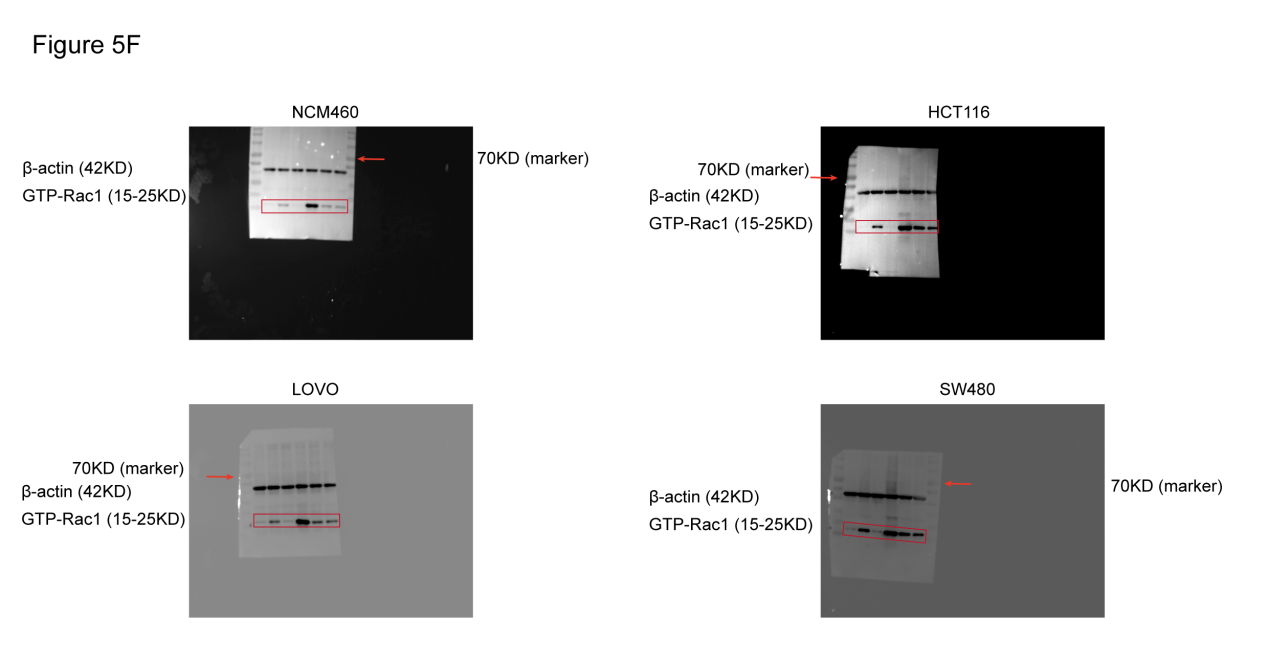

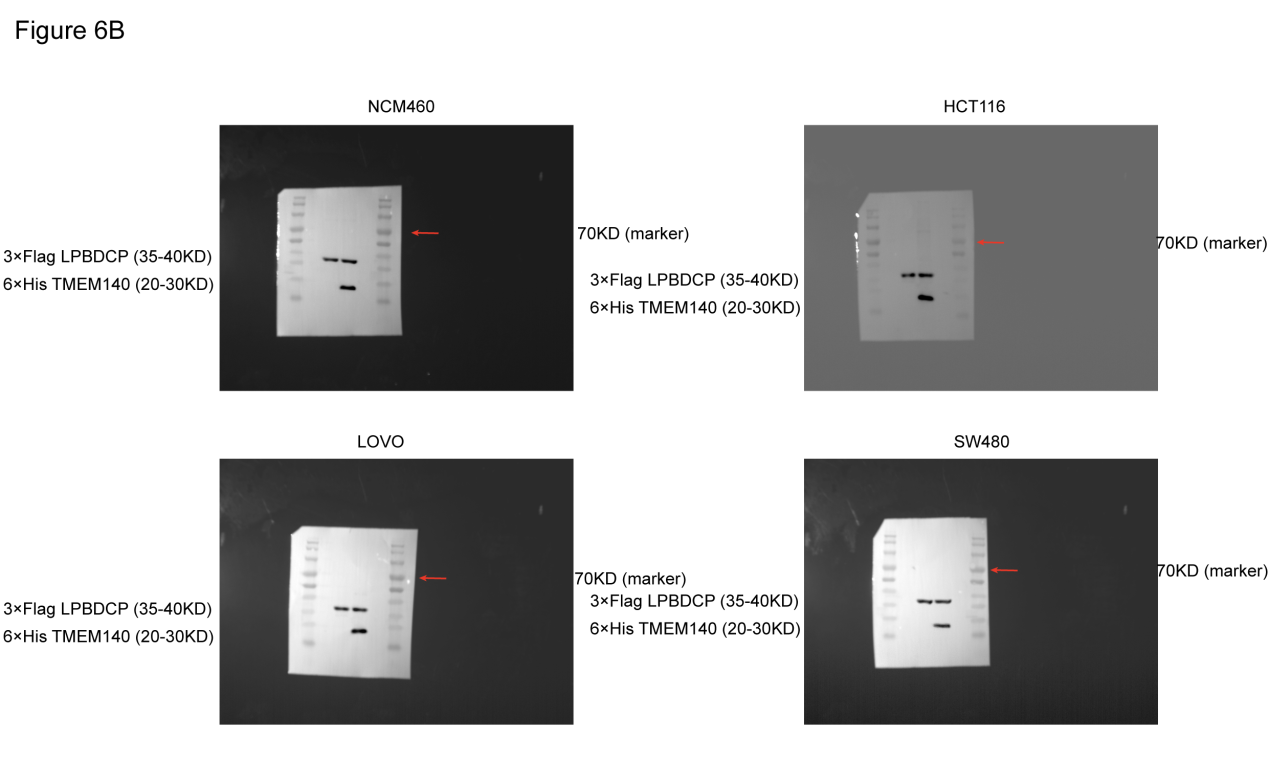

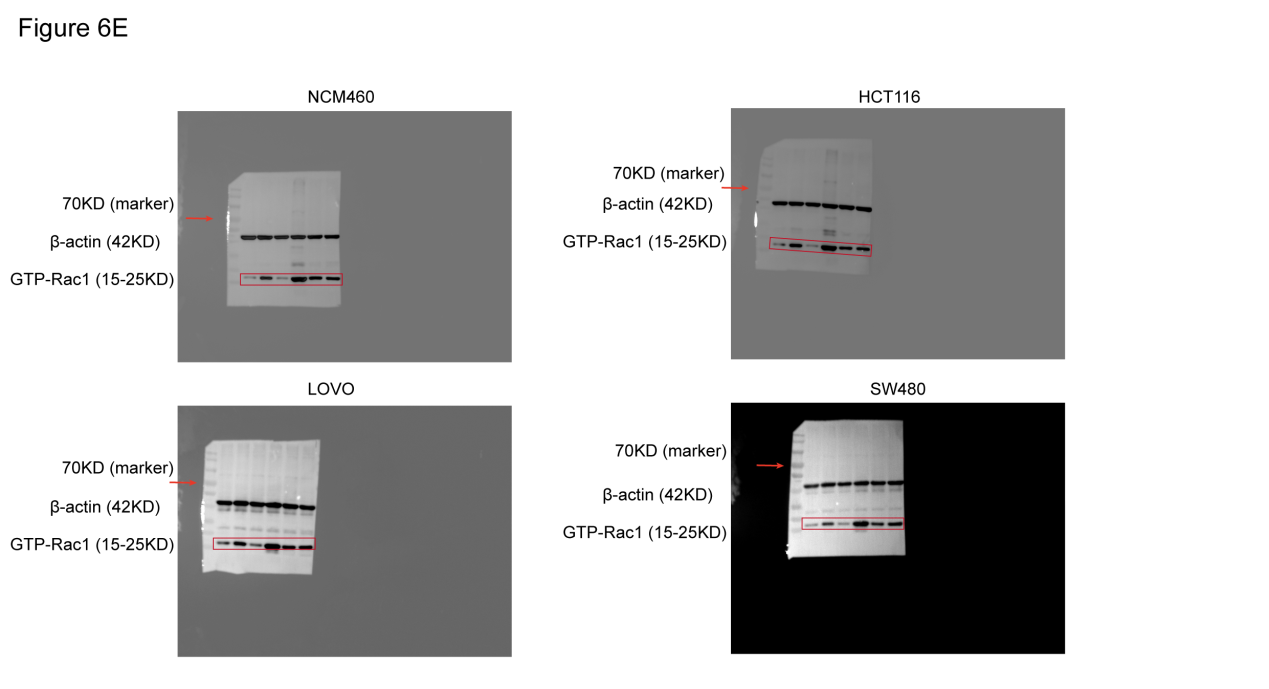


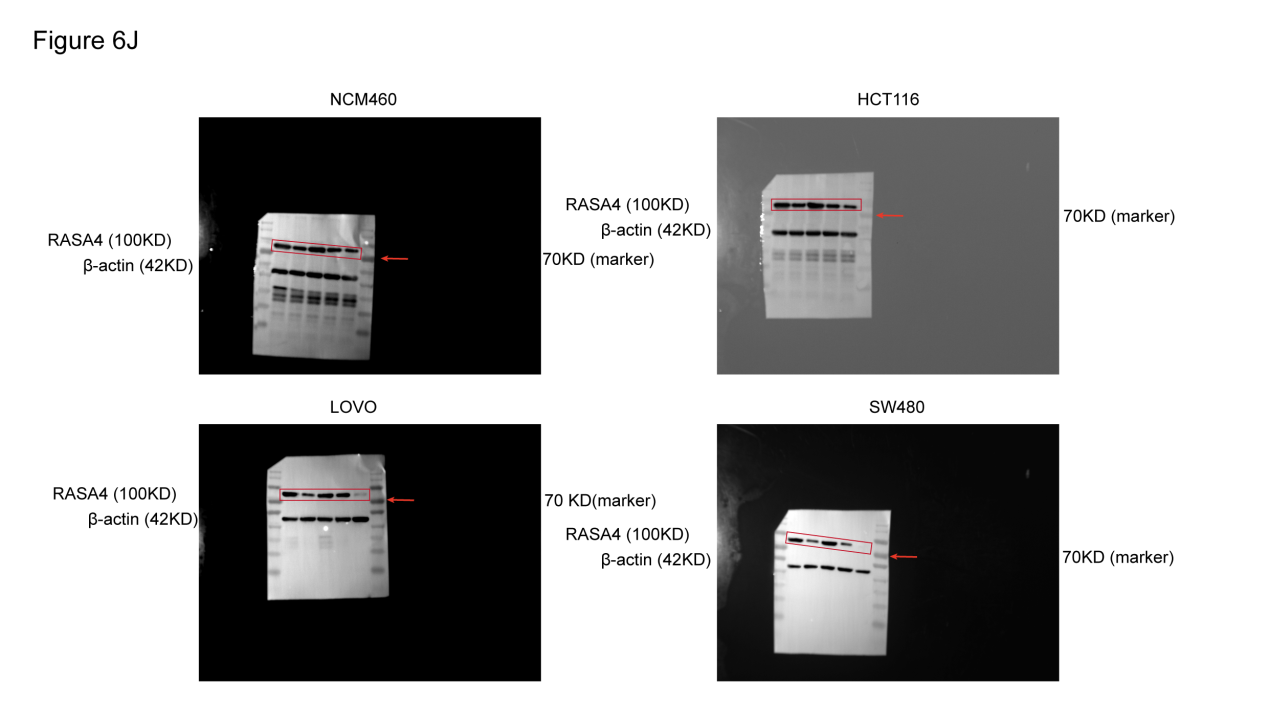

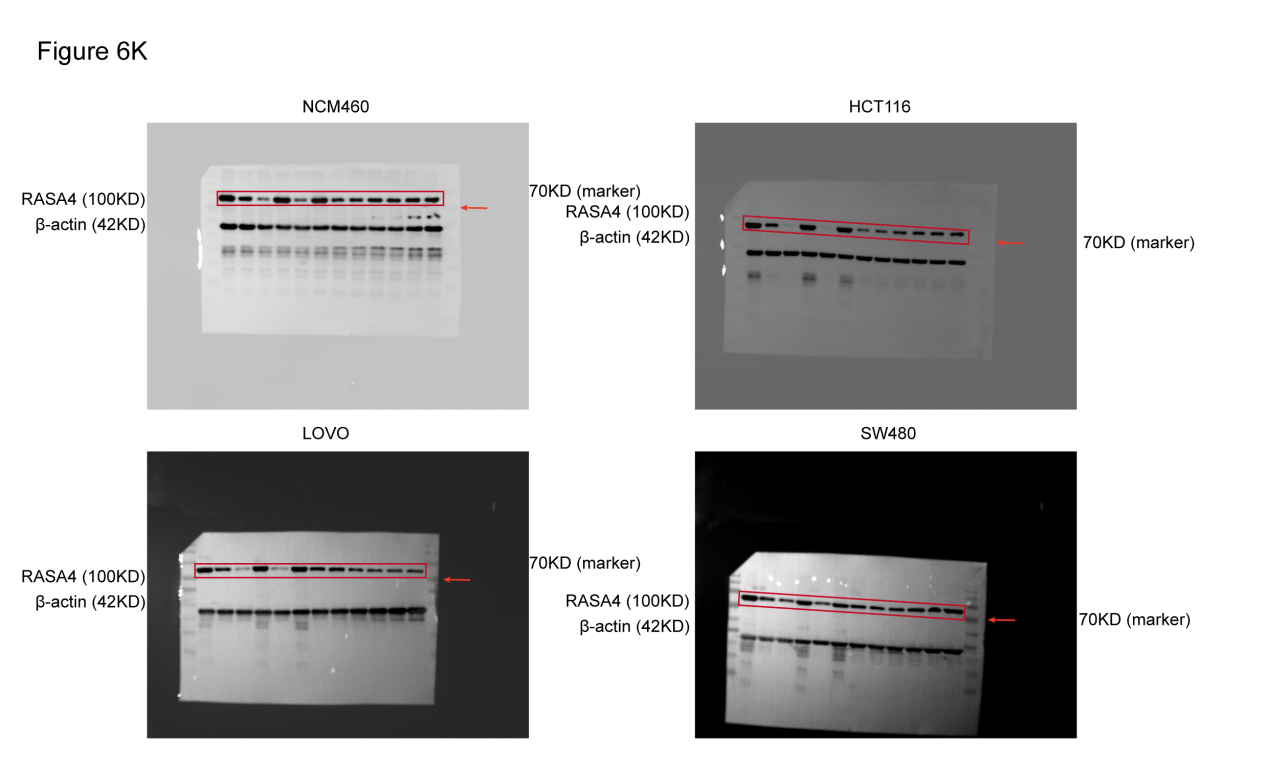

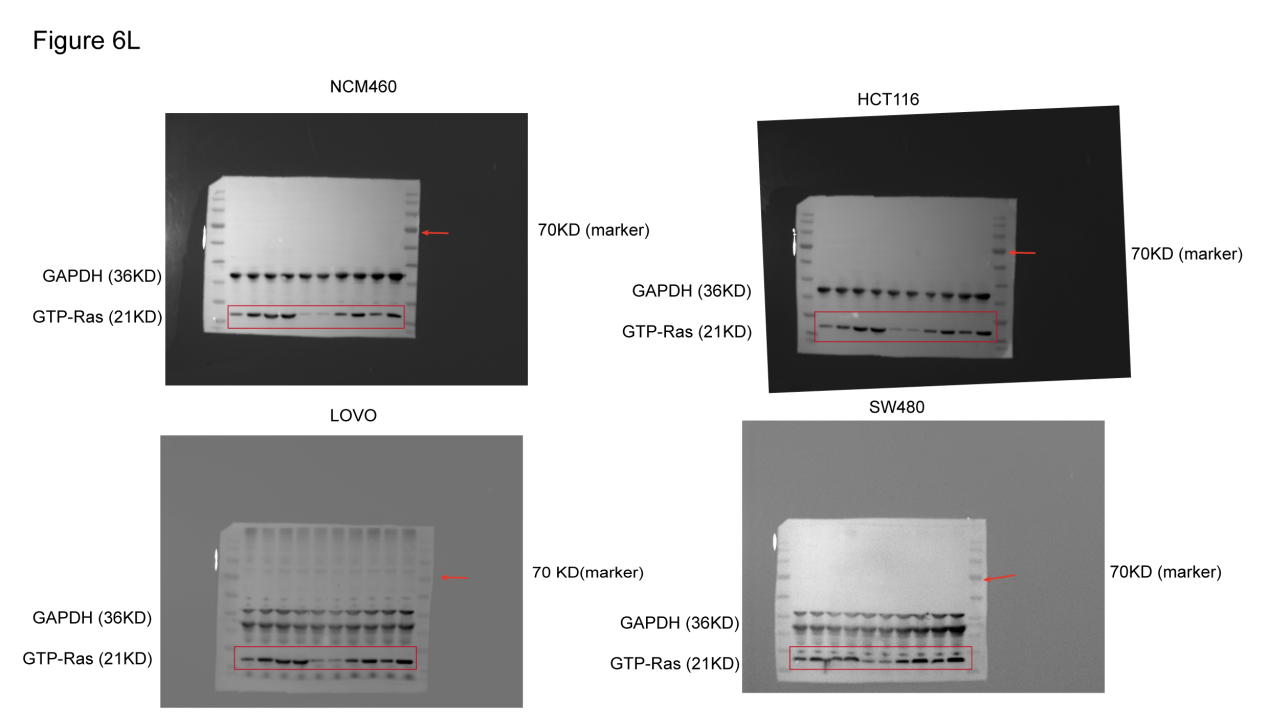

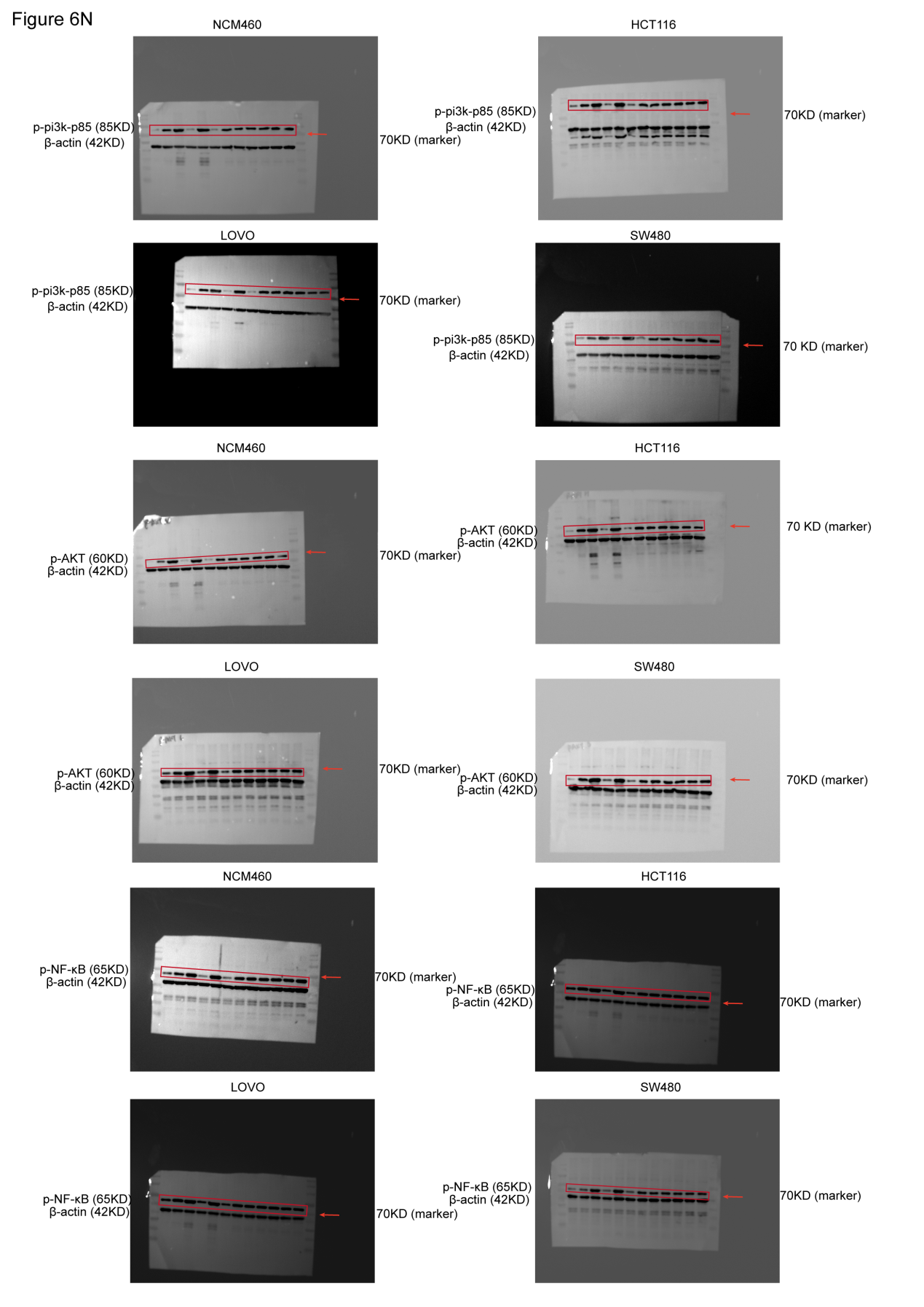

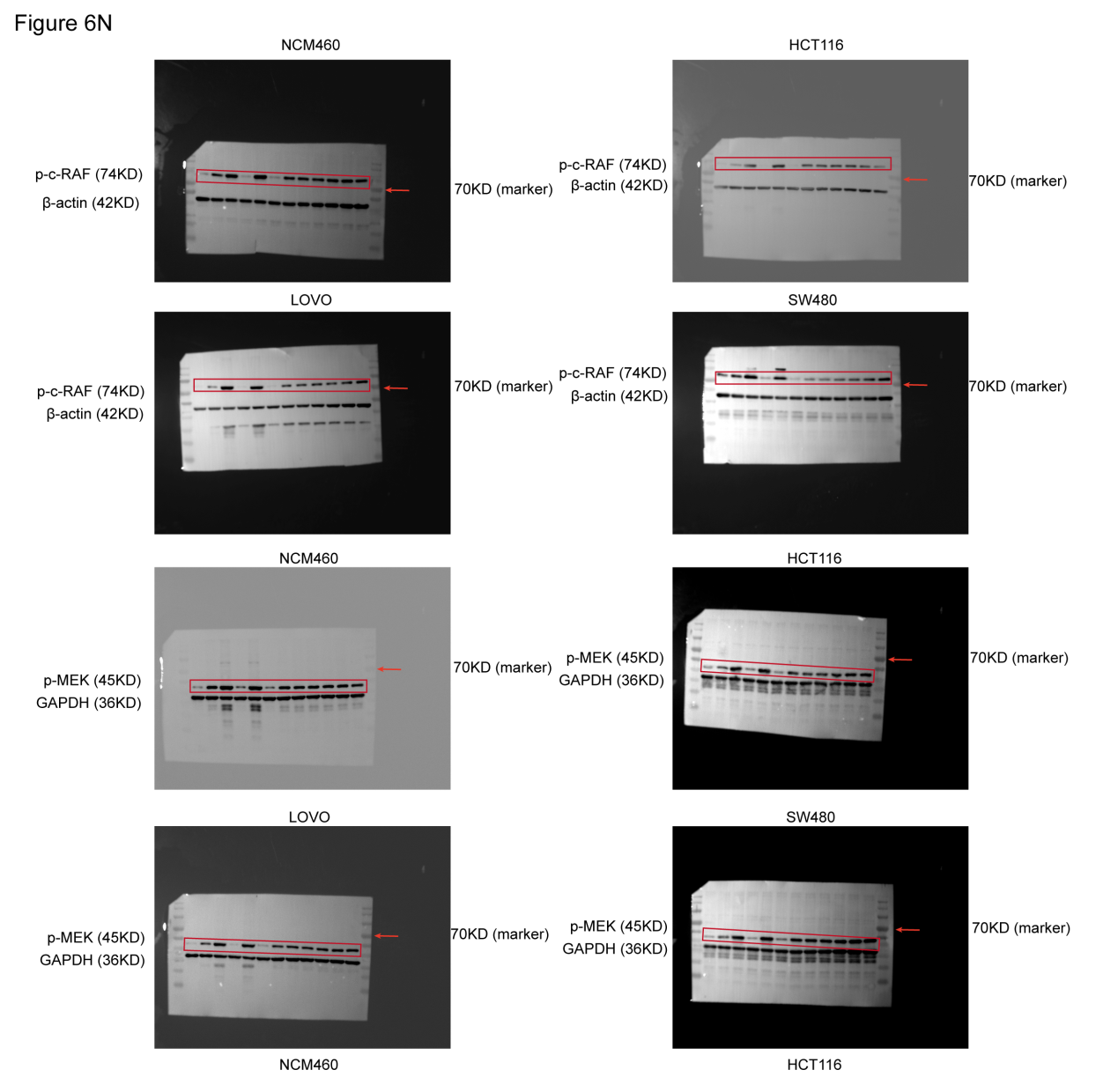

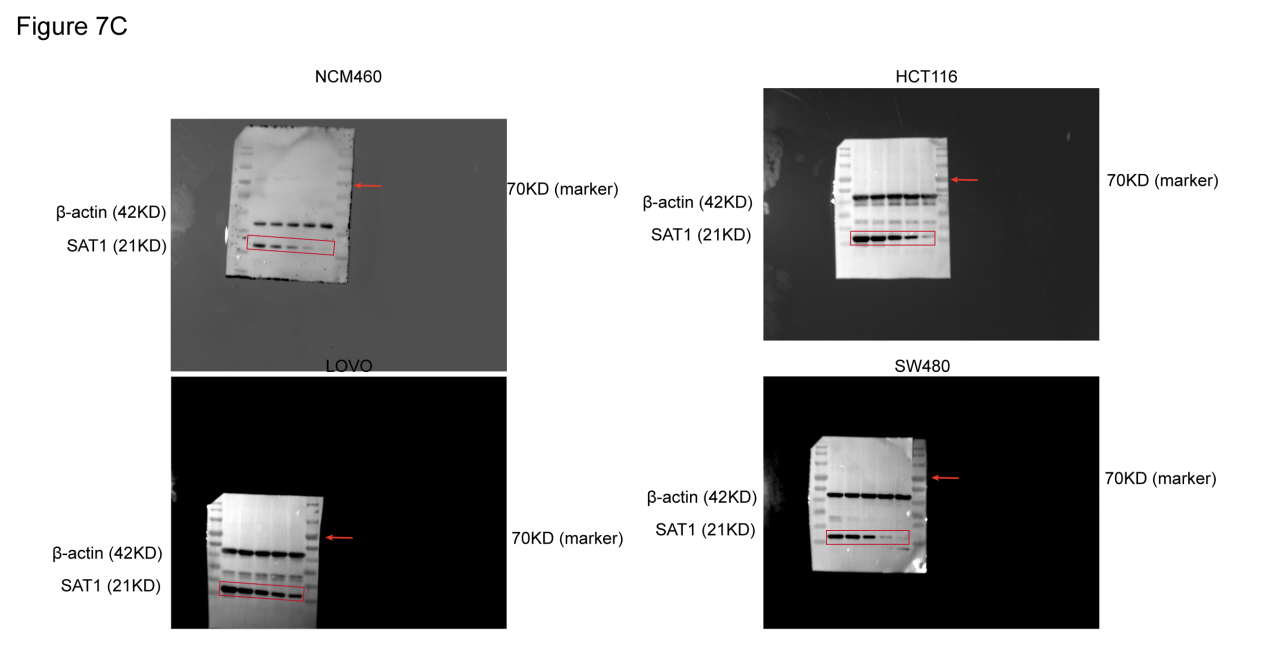

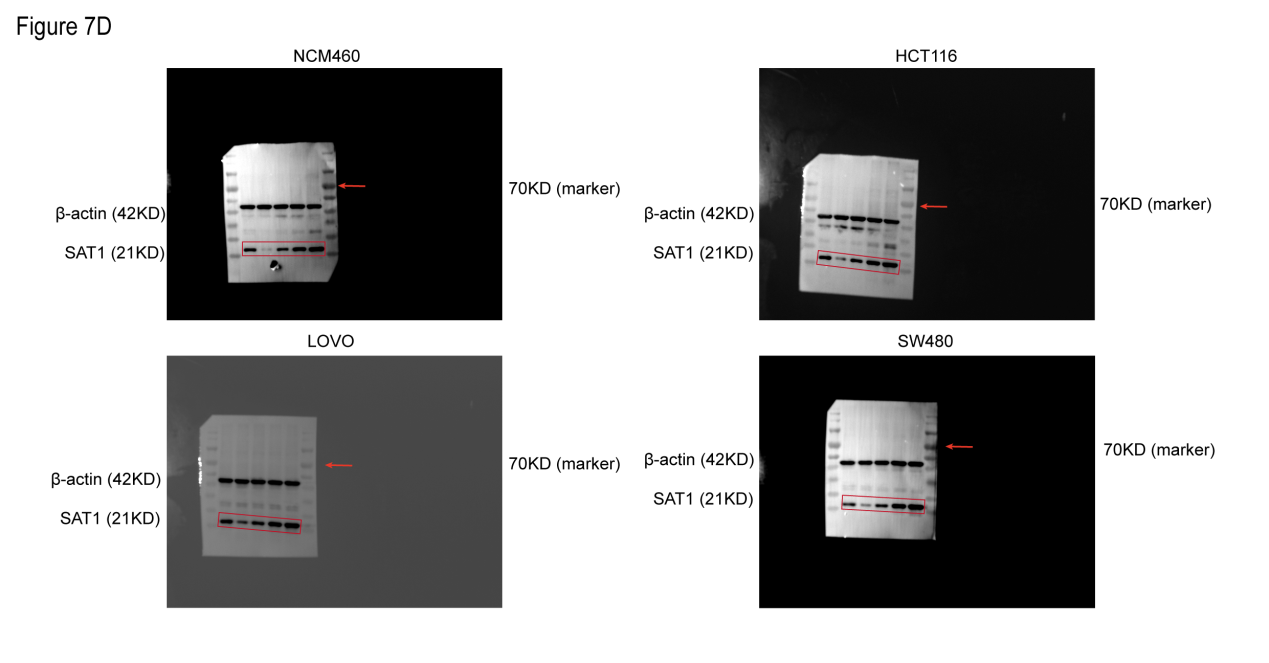

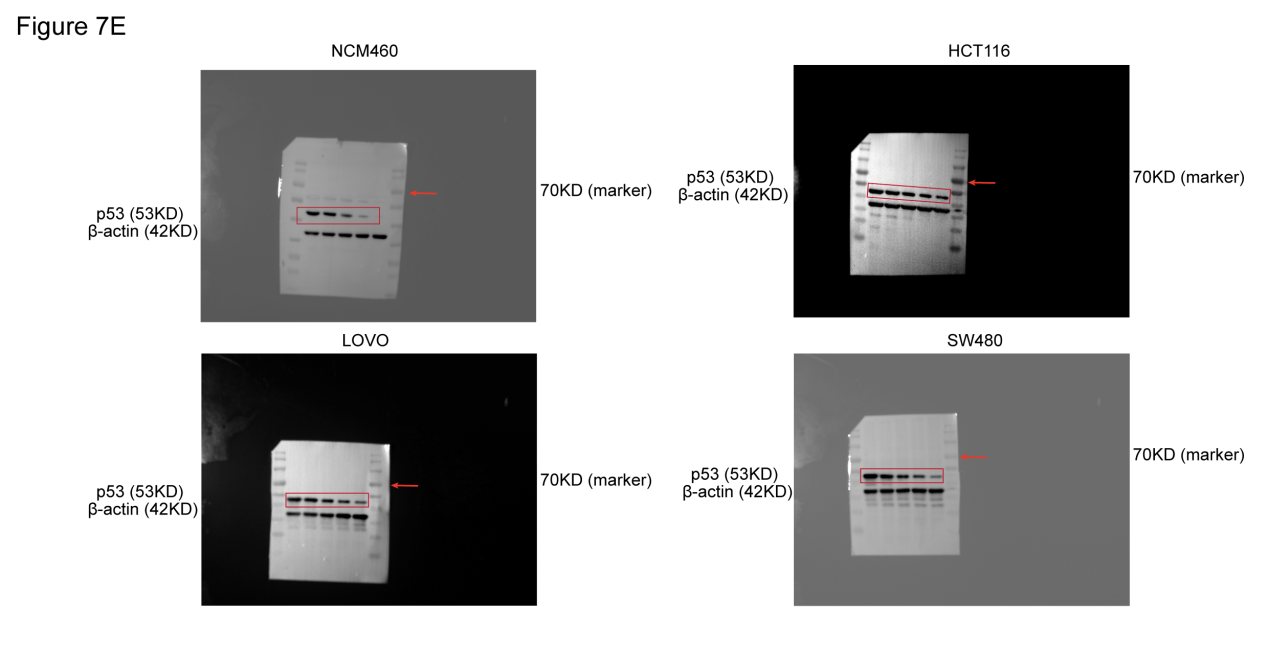

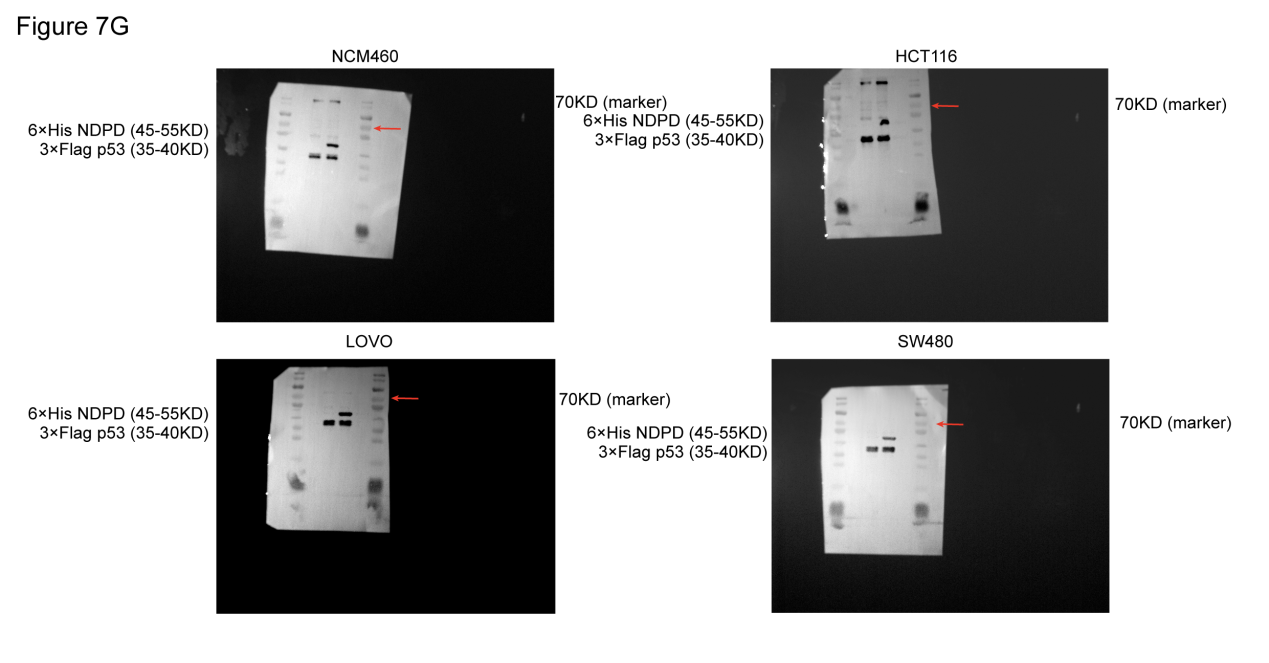


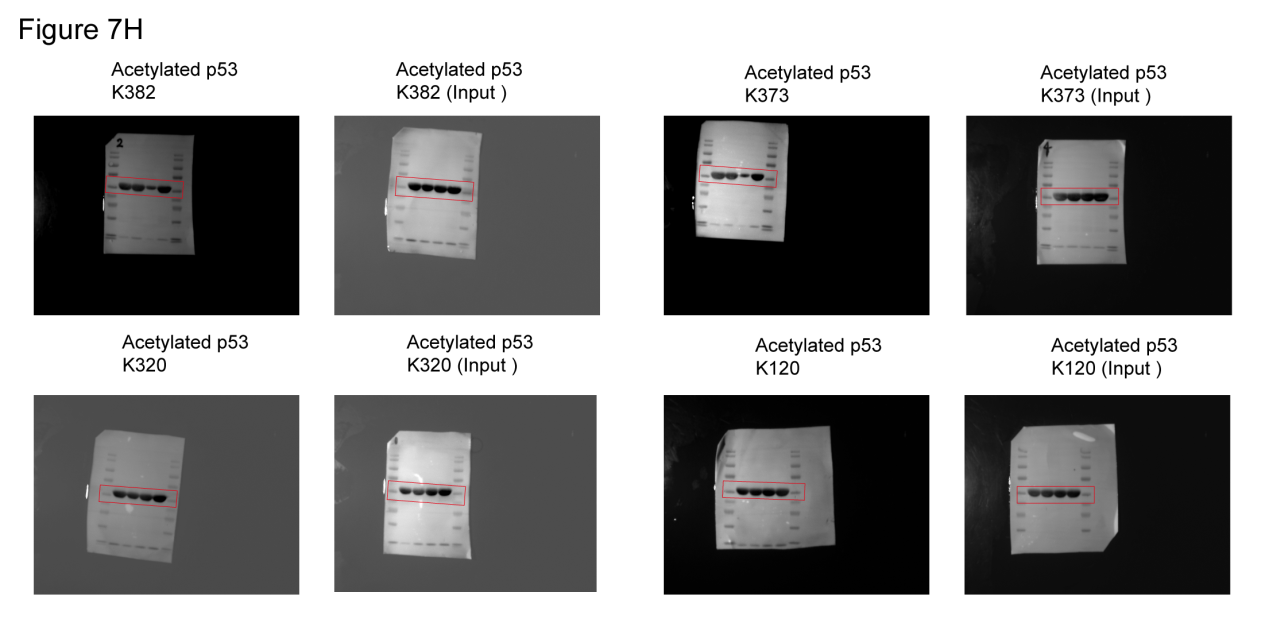

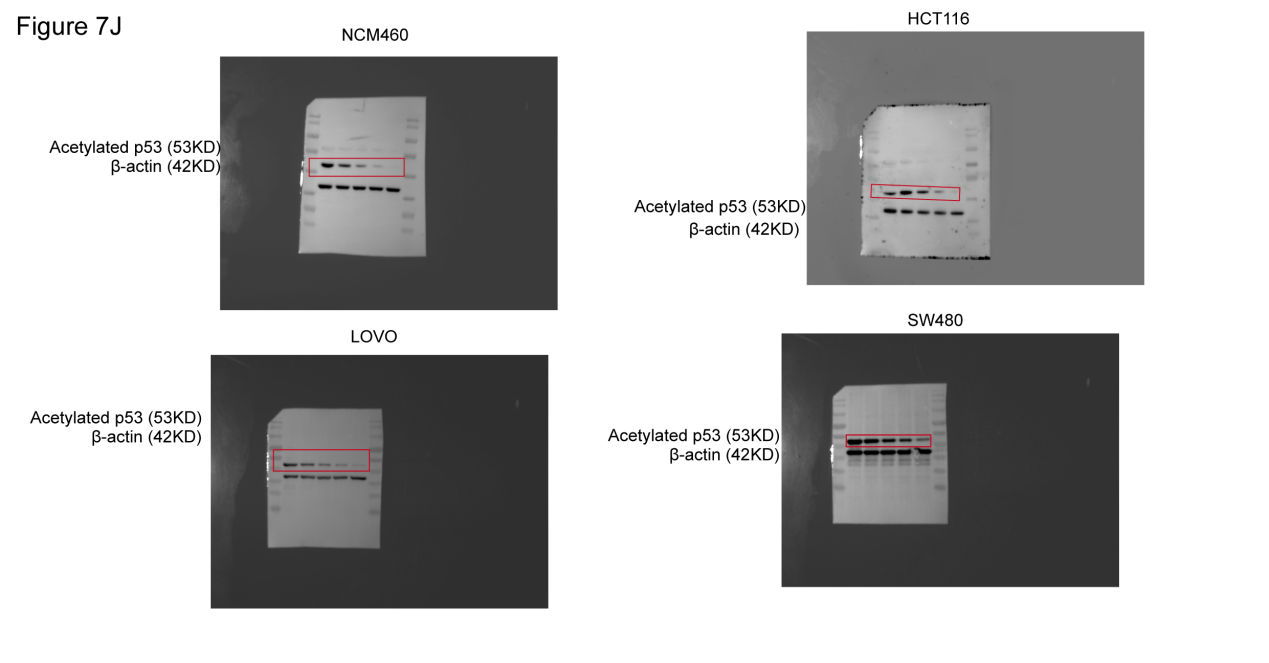

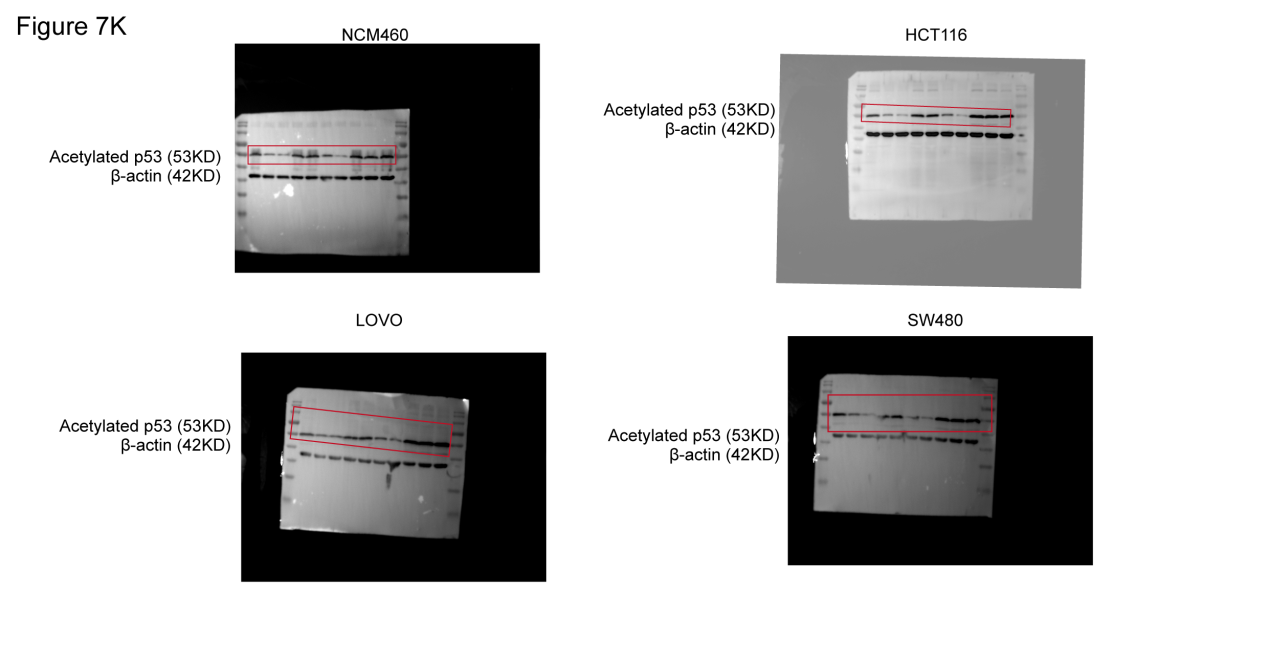

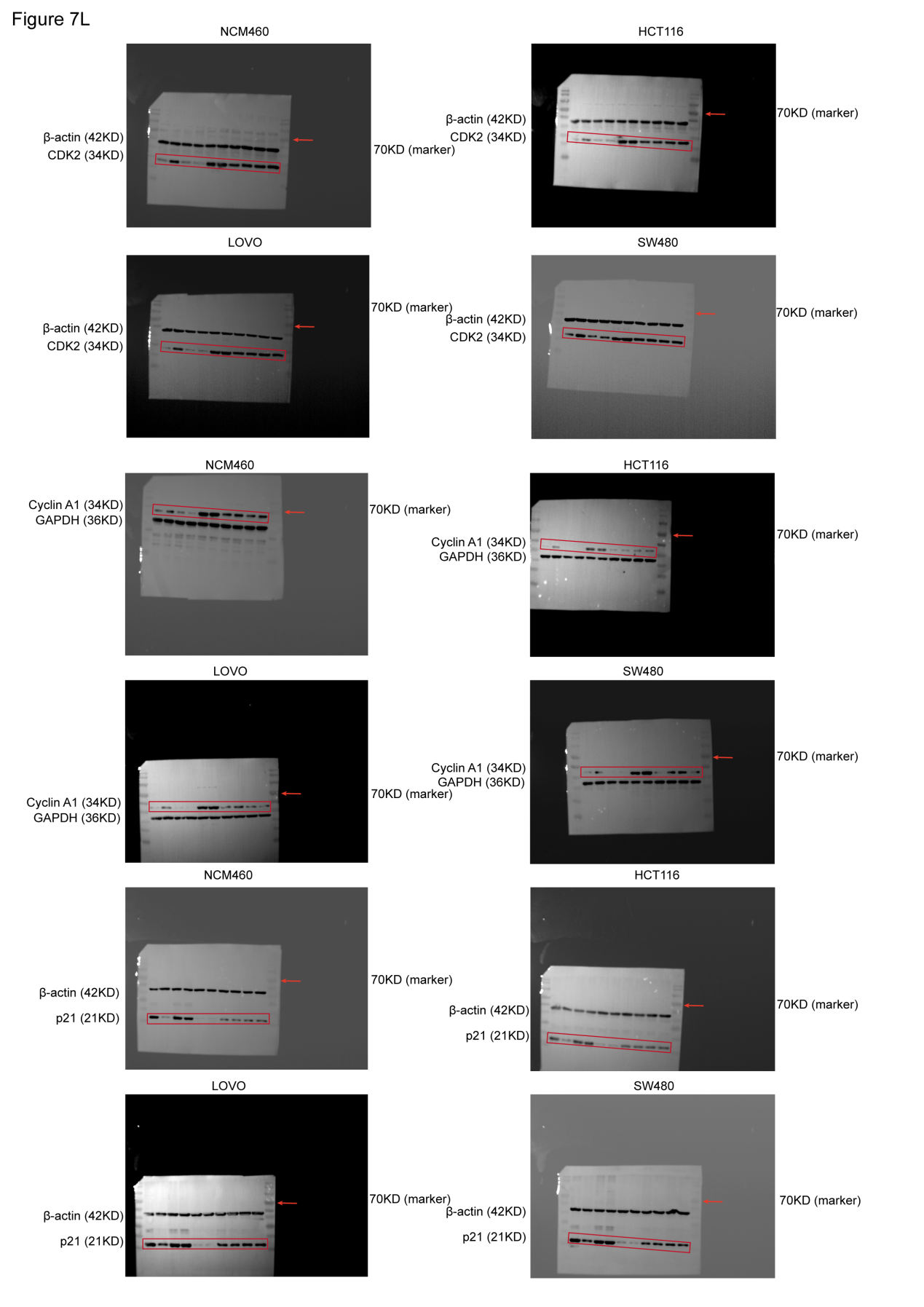

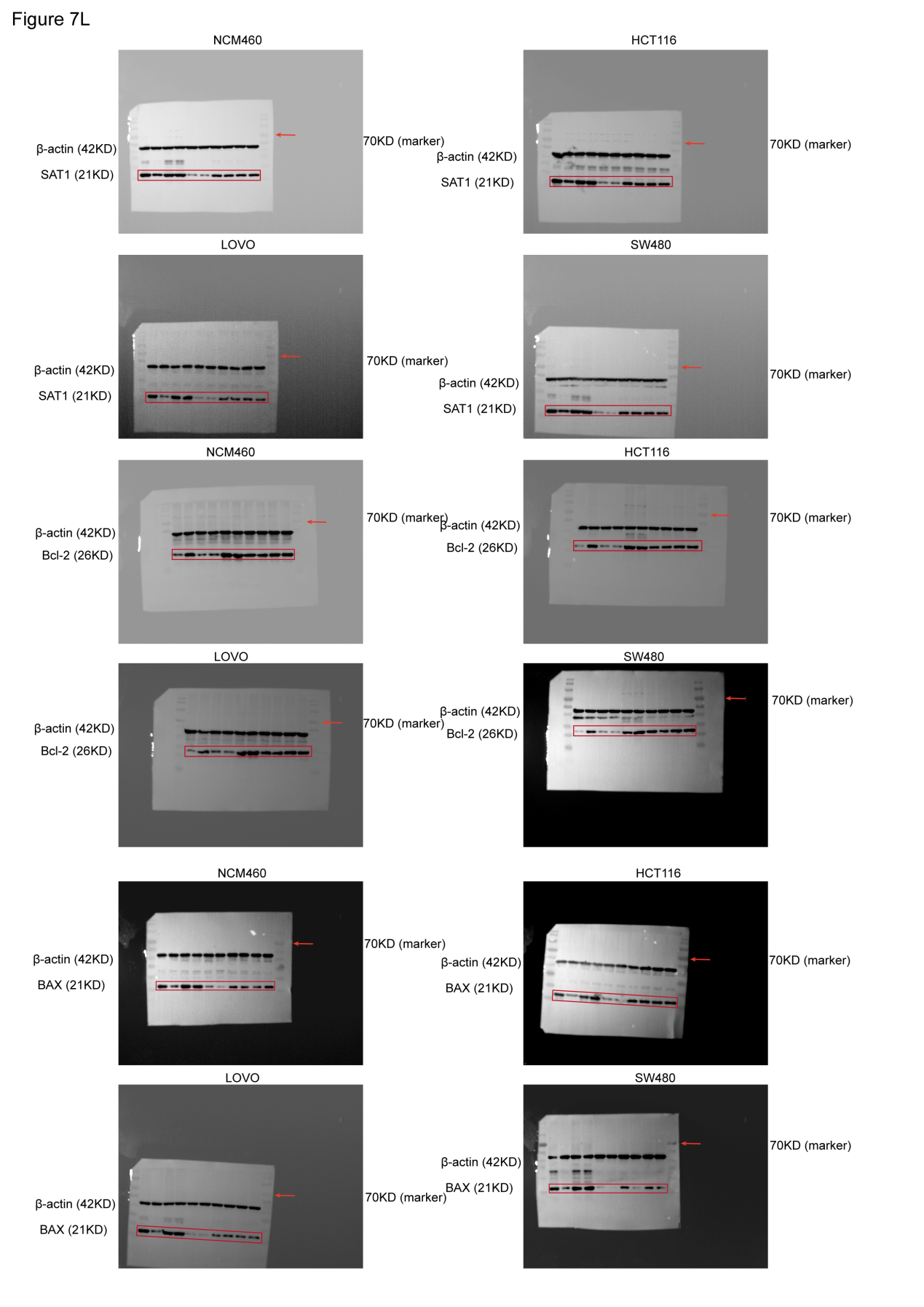

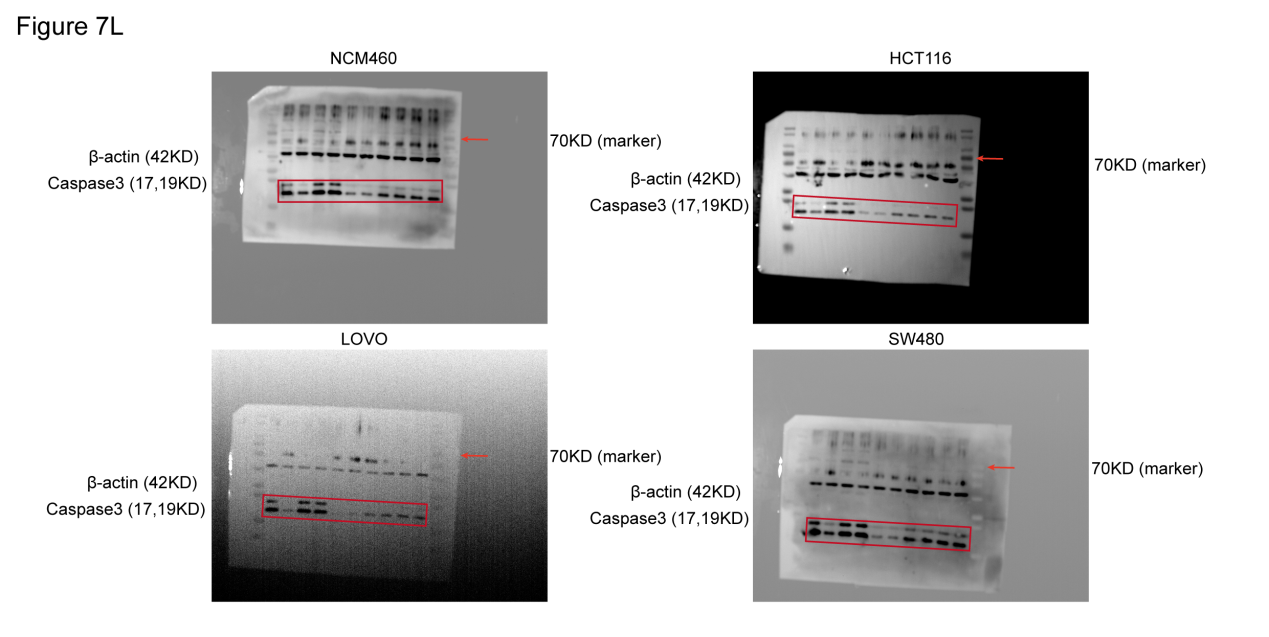


**Supplementary Figure 18**. **Original transmission electron micrographs.**

*E. coli*





*G. morbillorum*





*E. coli +* NCM460





*E. coli +* HCT116





*E. coli +* LOVO





*E. coli +* SW480





*G. morbillorum +* NCM460





*G. morbillorum +* HCT116





*G. morbillorum +* LOVO





*G. morbillorum +* SW480
